# Effects of Chlorine and Methyl Substitution on Structure, IR, and Raman Spectra of the Biomolecule 6-Chloro-3-methyluracil in Isolated and Solid States and in Base-Pair Formation

**DOI:** 10.3390/molecules31152665

**Published:** 2026-07-30

**Authors:** Mauricio Alcolea Palafox, Nataliya P. Belskaya, Nadya G. Hristova-Avakumova, Irena P. Kostova

**Affiliations:** 1 Chemical Physics Department, Chemistry Faculty, Universidad Complutense, 28040 Madrid, Spain; 2 Department of Technology for Organic Synthesis, Ural Federal University, 19 Mira Str., Yekaterinburg 620012, Russia; n.p.belskaya@urfu.ru; 3 Department of Medical Physics and Biophysics, Faculty of Medicine, Medical University of Sofia, 1431 Sofia, Bulgaria; nhristova@medfac.mu-sofia.bg; 4 Department of Chemistry, Faculty of Pharmacy, Medical University–Sofia, 2 Dunav Str., 1000 Sofia, Bulgaria; i.kostova@pharmfac.mu-sofia.bg

**Keywords:** 6-chloro-3-methyluracil, 3-methyluracil, scaling wavenumbers, DFT methods, Raman spectra, IR spectra, WC base pairs

## Abstract

The effect of the chlorine atom in the sixth position and methyl group in the third position of the uracil ring on the structural parameters, NBO charge distribution, and molecular properties of the 6-chloro-3-methyluracil (M6CU) biomolecule was analyzed in the isolated state, solid-state arrangement and within a double-stranded RNA microhelix. This effect, which was also compared to those with uracil and several uracil derivatives, leads to higher reactivity and the special properties of this pharmaceutical compound. Several correlations were established using MP2 and several DFT methods. The structural characterization of M6CU and a detailed analysis of the experimental FT-IR and FT-Raman spectra in the solid state was another aim studied in detail. For an accurate assignment, a simple approximation to a crystalline unit cell was optimized using a trimer form and the wavenumbers were subsequently corrected using several scaling procedures. The modified nucleobase M6CU forms base pairs with the complementary adenine and, therefore, it could replace uracil in the helix; based on our calculations, it leads to a noticeable helix deformation. This computational finding suggests its possible potential effect by binding to cancer-related or viral RNA helices. Molecular docking also revealed that M6CU could act as an active ligand within the 1JPW active site of the target β-catenin/Tcf4 protein complex.

## 1. Introduction

Substituted uracil derivatives continue to attract broad scientific interest because they are fundamental nucleobases and involved in key biological processes. These processes include their use as inhibitors of nucleic acid metabolism, as anticancer, antidiabetic, and antioxidant agents [1,2,3,4], and as clinical radiosensitizers of RNA/DNA in tumor cells. Among these, a notable class of radiosensitizing molecules corresponds to halogenated uracils (5- and 6-XU), where X = F, Cl, Br and I [5], which are currently employed to minimize radiation-induced damage to healthy tissues during radiotherapy [6].

In addition, among these halogenated pyrimidines [7], synthetized in the 1950s as potential antitumor agents acting through disruption of nucleic acid replication, particular attention was given to those which bear a halogen atom at the fifth position of the uracil ring. These structures make them closely resemble thymine, in which the methyl group is replaced by a halogen atom, enabling them to readily substitute thymidine in DNA in vivo. Consequently, many of these 5-XU halo-derivatives exhibit notable antitumor and antiviral properties [8,9,10,11,12,13].

However, halogenated derivatives bearing a substituent at the sixth position are scarcely investigated [14,15], leading to an underestimation of their potential clinical relevance. Both fifth- and sixth-substituted isomers are attached to a carbon atom, although within distinct environments, which results in pronounced differences in their electronic bonding and in their pharmacological properties. Halogen substitution in the sixth position appears to be more difficult than in the fifth position, particularly in terms of nucleobase binding with the furanose ring and the further phosphorylation process required for incorporation into the growing DNA strand. However, sixth-position-substituted uracil derivatives also exhibit significant potential biological activity. For example, they represent some of the most active inhibitors of thymidine phosphorylase (TP) [16]. Among these 6-halouracils, the compound containing a chlorine atom, the 6-chlorouracil (6-CU) molecule, shows antitumor activity as a ligand in complexes with several metal(II) ions [17], such as Co(II), Ni(II), Cu(II), Zn(II), Pd(II), Ru(II), Mn(II), and Rh(II).

DNA methylation is involved in several genetic alterations and contributes to the initiation of carcinogenic processes. In the case of uracil, this modification markedly affects its properties and noncovalent interactions with metal ions, as well as in the stability of its nucleic acid [18]. These damaged nucleobases have been reported to exhibit higher acidity than the normal nucleobases [19], and this increased acidity appears to facilitate DNA strand breakage [20].

Among the five possible damaged methylated uracils—1-methyluracil, 3-methyluracil (3MU), 6-methyluracil, 1,3-dimethyluracil and 5,6-dimethyluracil—3MU is of particular interest. 3MU is capable of forming Watson–Crick, wobble and sugar-edge H-bond base pairs, similar to those observed for canonical uracils [21]. It is a simple uracil derivative that has been investigated both theoretically [22] and experimentally using IR spectroscopy under matrix isolation [23] and in the solid state [24].

Owing to the antitumor activity of the 6-CU molecule when acting as a ligand in metal complexes, and the ability of the 3MU molecule to participate in noncovalent interactions with metal ions and in base-pair formation within DNA/RNA helices, the combined 6-chloro-3-methyluracil (M6CU) scaffold emerges as a promising donor–acceptor ligand for metal ion coordination [25,26]. Consequently, M6CU is now regarded as an important pharmaceutical compound (also known as an Alogliptin intermediate), and serves as a key precursor in the syntheses of pharmaceuticals, pesticides, coatings, etc.

M6CU is used in drug synthesis, for example, in the preparation of antibacterial agents that act as *Helicobacter pylori* glutamate racemase inhibitors, as well as an intermediate in the production of precursors for type 2 diabetes treatments. We found that M6CU induces concentration-dependent changes in the intensity of the analytical signal measured in free-radical-containing model systems using spectrophotometric and chemiluminescent assays. The copper(II) complex of M6CU exhibits remarkable biofunctional properties, showing significant antibacterial activity against both Gram-negative (*E. coli* and *P. aeruginosa*) and Gram-positive (*S. aureus* and *B. cereus*) bacteria [25]. A structurally related compound, 6-chloro-1-methyluracil, has been investigated for its DNA-binding ability, leading to DNA cleavage activity [27]. Another derivative, 1,3-dimethyl-6-chloromethyluracil, shows potent and selective antiviral activity against Sendai virus (SV) replication [28]. In light of these and other related findings, the present study focuses on M6CU by first examining this compound in the ground state from both structural and spectroscopic perspectives.

The molecular structures and spectroscopic properties of 5-substituted uracils have been extensively investigated [29,30,31,32,33], whereas considerably less attention has been paid to 6-substituted uracils, especially 6-CU [17]. A similar imbalance is observed with methylated uracils, where 5-methyluracil (thymine) has been widely studied [34,35,36,37] while 3MU has received much less attention [22,23]. M6CU has also been briefly characterized and remains little studied, although its crystal data [26] and copper(II) complex [19] have been reported. In this context, we now present a more comprehensive and detailed investigation of M6CU.

Therefore, M6CU appears to be an attractive uracil derivative and could become an interesting multifunctional ligand for the synthesis of bioactive coordination compounds. It represents an important pharmaceutical product that has not yet been studied from a structural and spectroscopic point of view. In particular, the impact of chlorine and methyl substituents at the sixth and third positions on its molecular properties, as well as their theoretical effect on the WC base pair and on the RNA microhelix, has not yet been analyzed.

The aims of the present work involving M6CU were the following: (i) To identify the effects of the chlorine atom and methyl moiety on the molecular properties of the uracil molecule and its noncovalent interactions. (ii) To characterize, for the first time, the molecular structure and vibrational bands of this molecule in the isolated state. (iii) To achieve an accurate assignment of the experimental IR and Raman spectra in the solid state using a trimer form. (iv) To describe the WC pair formation and the structural distortion induced when this molecule is incorporated into a double RNA:RNA microhelix. (v) To analyze the impact of this modification on the target protein of the *β-catenin/Tcf4* complex. (vi) To describe the radical-scavenging properties and antioxidant/prooxidant effects of M6CU, which have not been reported so far.

Based on this knowledge, it is expected that a combination of such an M6CU bioactive ligand with metal ions may provide synergistic effects in the experimental pharmacological activity of the obtained coordination compounds. This study is part of our ongoing research on bioactive lanthanide complexes [38,39].

## 2. Results and Discussion

**Figure 1:**
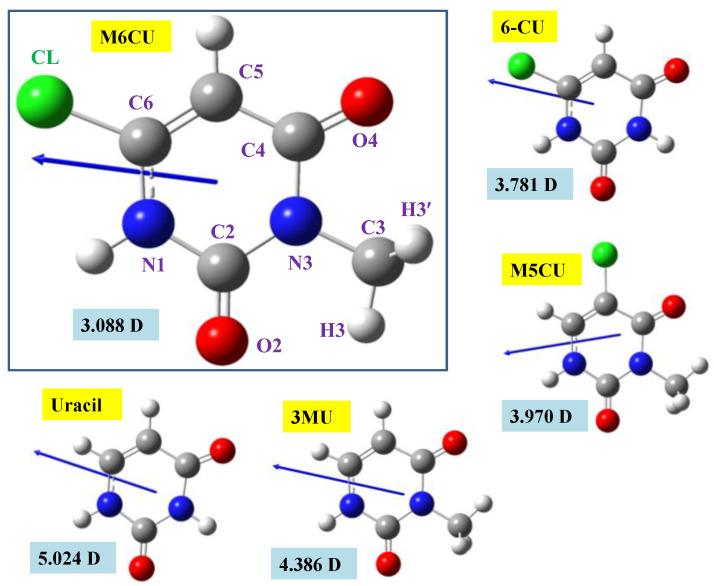
Labeling of the atoms in 6-chloro-3-methyluracil (M6CU) with its dipole moment vector in a blue arrow. The structure and values at the MP2/6-311++G(3df,pd) level in the molecules under comparison are also included.

The optimized molecular structure of M6CU with standard atomic numbering is shown in Figure 1. Calculated bond lengths and angles obtained by MP2 ab initio and by B3LYP, CAM-B3LYP and M06-2X DFT methods with the 6-31G(d,p) and 6-311++G(3df,pd) basis sets are listed in Table S1 (Supplementary Materials Section), while those obtained by B3LYP and MP2 with the 6-311++G(3df,pd) basis set are collected in Table 1. The corresponding NBO atomic charges are presented in Table 2 and Table S2.

### 2.1. NBO Atomic Charges

Atomic charges are of considerable importance in the study of molecular systems, as they can be associated with several molecular properties, such as electronic structure, dipole moments, molecular polarizability, and reactivity. Therefore, they were also selected as parameters to examine the effect of the chlorine atom and methyl group bonding to the uracil ring. The calculated values obtained using several methods and basis sets are summarized in Table 2. For comparison, the charges obtained in uracil, 6-CU and 3MU derivatives were also included. A large difference was observed between the computed values by MP2 and those obtained using DFT methods, with a notable increase in all positive and negative charges obtained by MP2, in particular those on the C2 and C4 atoms.

**Figure 2:**
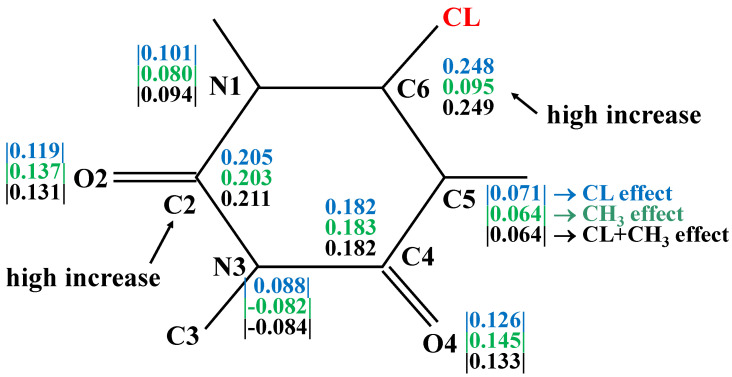
NBO atomic changes with the chlorine in the sixth position, and with the methyl group in the third position of the uracil ring. Values in blue correspond to the effect of the chlorine atom alone (comparison of 6-CU vs. uracil), values in green correspond to the effect of the methyl group alone (comparison of 3MU vs. uracil), and values in black color correspond to combined effects of both chlorine and methyl substituents in M6CU (comparison of M6CU vs. uracil).

**Figure 3:**
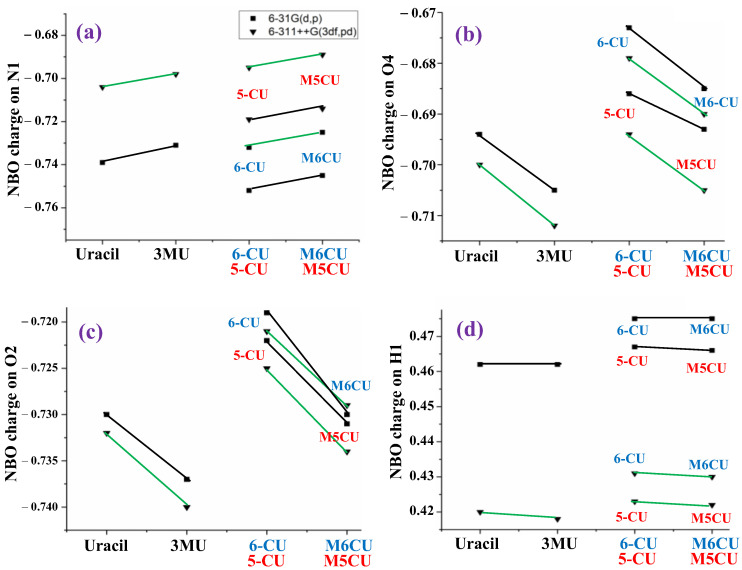
Effect of the chlorine and methyl substitution on the NBO charges of selected atoms of the uracil, 3MU, 6-CU, 5CU, M6CU and M5CU molecules at the MP2/6-31G(d,p) (in black) and MP2/6-311++G(3df,pd) levels (in green). (**a**) Relationship with the NBO charge on N1 in the molecules under study, (**b**) with the NBO charge on O4, (**c**) with the NBO charge on O2, and (**d**) with the NBO charge on the amino hydrogen H1.

Also, all charges by the M06-2X method (Table S2) were higher than those by B3LYP, and closer to MP2 values, which were taken as the reference. The effect of the methyl and chlorine substituent atoms in the atomic charges of the uracil molecule is plotted in graphical form for all atoms in Figure 2, and for the selected N1, O2, O4 and H1 atoms in Figure 3. Both methyl and chlorine substituents remarkably change the uracil charges, in most cases increasing its positive or negative values.

For the basis set increase from 6-31G(d,p) to 6-311++G(3df,pd) with the MP2 method, although it significantly changes the values, the effects observed with the methyl and chlorine substituents clearly remain, Figure 3. The trends (line slope) are the same with the 6-31G(d,p) basis set (in black) as with the 6-311++G(3df,pd) basis set (in green).

The main effects observed by MP2 are summarized below, while additional data are provided in the Supplementary Materials:

(i) In M6CU, the very low positive NBO charge of the chlorine atom of 0.054*e* (0.057*e* in 6-CU), compared to the charge of hydrogen atom 0.198*e* occupying the same position of the uracil molecule, leads to an alteration in the electronic environment at this sixth position. Therefore, the positive charge on C6 is noticeably increased in M6CU with 0.301*e* vs. 0.184*e* in uracil and, consequently, the negative charges on C5 and N1 are remarkably increased, Figure 2. This change in the NBO charges is the same as that observed in 6-CU and with the same values, indicating that the 3-methyl group has little influence on these changes.
(ii) The negative NBO charge on N1 in the uracil molecule increased upon methylation in 3MU, and this increase is made larger by the presence of the chlorine atom in 6-CU. Although both substituents increase the negative charge on N1, the combined substitution in M6CU results in only a slight decrease in its negative value compared to 6-CU. This indicates that the N1 charge is mainly affected by the chlorine atom, whereas additional methylation at the third position causes only minor changes in its value, Figure 3a.
(iii) In the uracil molecule, the negative charge on N3 is slightly larger than on N1, whereas the negative charge of the neighbor O4 atom is slightly smaller than that of O2. The chlorine substitution increases the negative charge of both nitrogens, with N3 remaining more negatively charged than N1. This feature in 6-CU is also observed in 5-CU. However, upon methylation this order is reversed, as in the case of M6CU.
(iv) The effect of the methyl substitution at the third position of the uracil ring is notably stronger than that of chlorine substitution at the O2 and O4 oxygens. A similar behavior is observed in both oxygens: methylation increases their negative charge by ca. 0.15*e*, whereas the chlorine substitution in M6CU slightly decreases it, Figure 3b,c. Chlorine substitution at the fifth or sixth position of the chlorine atom has little effect on their charge.
(v) The positive charge on H1 slightly increases upon the methylation in 3MU, Figure 3d, and this value is again increased upon chlorine substitution, particularly at the sixth position. The importance of this H1 atom arises from its role in the linkage between the sugar ring and the nucleobases to form a nucleoside. Therefore, a more positive charge on H1 indicates higher reactivity.
(vi) The charge on C2 is increased with the methylation in 3MU, 0.203*e* (Figure 2), similarly to the chlorine substitution, which leads to a slight increase in M6CU and to the largest positive value of 1.025*e* in the molecule.
(vii) The C6 atom has a very small positive charge of 0.184*e* in the uracil molecule, the lowest positive charge among all carbon atoms, similar to that observed in 3MU, 6-CU and M6CU, although its value is significantly increased (by ca. 0.12*e*) with chlorine substitution. This is because the chlorine atom withdraws negative charge from C6, changing it from a smaller positive value (0.184*e*) in uracil to 0.300*e* in 6-CU and 0.301*e* in M6CU.
(viii) The only carbon in the structure with a negative charge is C5, and its negative value is slightly increased with chlorine substitution and methylation.

### 2.2. Geometric Optimization of M6CU in the Isolated State (Monomer Form)

Due to the importance of this M6CU molecule, we consider it of interest to carry out a structural characterization using different theoretical levels. Therefore, its optimized structure was found to be planar in agreement with that calculated in uracil and 6-CU molecules [17], as well as in 3MU. The optimized values in all these molecules are included in Table 1 for comparison purposes. Unfortunately, only a few X-ray data have been reported of M6CU [26], and none concerning its torsional angles, but X-ray geometrical values are available for uracil, 3MU [41] and 6-CU [42]. Since the electron diffraction (ED) data are closer to the calculated values in an isolated state than those obtained by X-ray, and they are available for uracil [40], they are also included in the table, together with the experimental X-ray values reported for 3MU.

Comparing the values, a good agreement is observed between the calculated and experimental N1-C2, C2-N3, N3-C4 and C4-C5 bond lengths of uracil, which are only ca. 0.01 Å shorter than the experimental values, which falls within the reported experimental uncertainty, while the C5=C6 and C=O bonds appear ca. 0.005 Å longer. The largest deviation occurs for the N1-C6 bond that is 0.025 Å shorter than the experimental one. The difference in bond angles is ca. 2°. Due to the accuracy results obtained by MP2 in the isolated state of the uracil monomer, which are consistent with the gas-phase experimental data by ED, a similar level of accuracy is expected for the calculated values in M6CU and related uracil derivatives under study. However, significant discrepancies are observed between the 3MU monomer and X-ray crystallographic data, because in this case, as well as in M6CU, it is necessary to optimize at least the crystal unit cell, as described in the Section 2.3. The increase in the basis set from 6-31G(d,p) to 6-311++G(3df,pd) has little effect on the geometric parameters, improving some values compared to the corresponding X-ray ones but worsening other values. For this reason, and due to the fact that we are looking for trends in the geometric parameters (Figure 3), calculations with larger basis sets were not carried out.

Comparing the calculated N1-C2, N3-C4, C4-C5, N3-C3 and C6-CL bond lengths by B3LYP reveals that they are slightly longer than by MP2, and by contrast a shortening is observed in both C=O bonds similar to that observed in the uracil molecule. In general, the values by M06-2X are closer to MP2 than those by B3LYP, but the MP2 values in uracil and 3MU are the closest to the experimental ones (Table 1); for simplicity, therefore, they were mainly considered as a reference for the discussion of the values. The calculated geometrical values at the B3LYP-D3/6-311++G(3df,pd) level are almost the same as those obtained using B3LYP, which indicates the small effect of this D3 correction on the geometrical parameters. Because of this feature, the calculated values by this B3LYP-D3 method were not considered.

The main effects of the chlorine substitution at the sixth position of the uracil ring in the geometrical parameters and charges on the neighboring atoms in M6CU at the MP2/6-311++G(3df,pd) level were as follows:

(i) The chlorine substitution has little effect on the bond lengths and angles around the sixth position, only a small lengthening of 0.005 Å in N1-C2 and a very small shortening in C4=O and C2=O bonds which remain nearly unchanged. The bond angles are also slightly affected, although the effect on the *ipso* angle N1-C6=C5 appears opposite with 6-CU, which is slightly open (0.6°) vs. uracil, compared to that with M6CU, which is closed (0.2°) vs. uracil. In M6CU the effect observed in the neighbor C2-N1-C6 angle is a closening of 0.4° vs. uracil, while it remains almost unchanged in C4-C5=C6, as expected.
(ii) The bond angles are similarly little affected. Notably, the effect on the *ipso* angle N1–C6–C5 is opposite to that in 6-CU: it opens slightly (0.6°) relative to uracil, whereas in M6CU, it closes (0.2°) relative to uracil.
(iii) The very small effect of the chlorine atom in the C5=C6 bond (1.348 Å) is in accordance with that found in 5-ClU (1.350 Å), as well as with that observed for the bromine atom in 5-BrU and 6-BrU molecules [17,29]. Similarly, an almost negligible effect in the C4-C5 bond (1.453 Å) is found in agreement with that in 3MU and 6-CU (1.451 Å), although in 5-CU it is 0.01 Å longer. Therefore, similar behavior is expected with our M6CU molecule, as well as in the optimized 5-chloro-3-methyluracil (M5CU).

In general the trends observed with the methyl and chlorine substituents using the 6-311++G(3df,pd) basis set are very similar to those obtained with the 6-31G(d,p) basis set, Table S1.

#### 2.2.1. Differences Between 5-Chloro-3-methyluracil (M5CU) and M6CU

Comparison of geometric parameters and energies for M5CU and M6CU at the MP2/6-31G(d,p) level revealed the following features:

(i) The C2=O bond length is unaffected by the chlorine atom substitution, with almost the same value in 6-CU as in 5-CU, 1.212 Å; therefore, it has almost the same value in M6CU (1.216 Å) as in M5CU (1.217 Å). However, in the C4=O bond length there are small differences. Thus, in 6-CU its value in C2=O is slightly lower than in C4=O, 1.217 Å, but due to the influence of the neighboring chlorine atom in 5-CU it is the same in C2=O and C4=O, with the value of 1.213 Å. Consequently, small differences are expected for M6CU, with a value of 1.219 Å in C4=O, which is slightly longer than in M5CU, 1.216 Å. This feature has also been reported in other halo-uracil derivatives with a longer C4=O bond in 6-XU than in 5-XU [17,29].
(ii) The C-CL bond length in M6CU (1.708 Å) is slightly longer than that in M5CU (1.706 Å). A similar trend was observed comparing 6-CU (1.708 Å) with 5-CU (1.705 Å), with a small lengthening of 0.003 Å, as well as in the comparison between 6-bromouracil (6-BrU) and 5-bromouracil (5-BrU) [29].
(iii) The N1-C2 and N3-C4 bonds are longer in M6CU (1.389 and 1.411 Å, respectively) than in M5CU (1.380 and 1.407 Å, respectively). As a consequence of this lengthening, and as is expected in M6CU, the neighbor N1-C6 and C2-N3 bonds are shortened by a similar amount in M6CU compared to M5CU.
(iv) 6-CU is 12.83 kJ/mol more stable than 5-CU (11.3 kJ mol with the 6-31G(d,p) basis set); therefore, M6CU also exhibits higher stability by 12.84 kJ/mol than its M5CU counterpart (11.077 kJ/mol with the 6-31G(d,p) basis set). This MP2 value is in agreement with the calculations performed at the B3LYP/6-311++G(3df,pd) level, where 6-CU is 13.4 kJ/mol more stable than 5-CU [17]. Moreover, experimentally [43], 6-CU has been found to be more thermodynamically stable than 5-CU by 31.0 kJ/mol, which appears almost three times larger than the value obtained in our calculations by MP2. This feature has been reported in other halogenated uracil derivatives [17,29], and it is probably due to a different electronic distribution on the uracil ring in 5-XU (X = halogen) that in 6-XU, and where the NBO charge on C5 is −0.265*e* in 5-CU, whereas the charge on C6 has a positive value of 0.273*e* in 6-CU. Also, a weak interaction between the chlorine atom and the neighboring atoms is also expected to occur.

#### 2.2.2. Dipole Moments

Owing to the importance of the dipole moment vectors and their magnitudes, they were calculated by MP2 for the M6CU molecule as well as for the nucleobases of uracil, 3MU, 6-CU and M5CU, and included in Figure 1, while the values obtained using different DFT methods and basis sets are presented in Table S3 and only those with the 6-311++G(3df,pd) basis set in Table 3. It is noted that the value by MP2 in M6CU is significantly higher than those determined by DFT methods, and its value increases with increasing basis-set size.

Because the chlorine atom has a much larger mass than hydrogen and a very low NBO positive charge, the center of mass and the electron density distribution of the uracil molecule is distorted upon chlorine substitution. Thus, the chlorine atom leads to a decrease in the dipole moment, from 5.024 D in uracil to 3.781 D in 6-CU, and although the direction of the dipole vector remains almost unchanged, Figure 1, its center moves toward the chlorine atom. This change in the orientation and NBO charge has been observed with other halo-substituents [17], leading to a slight reduction of ca. 10% in the dipole moment magnitude with 5-XU nucleobases and to a much larger reduction of ca. 35% with 6-XU.

The non-polar methyl group in the 3-methyl-substituted uracil ring (3MU) leads to a decrease in the dipole moment to 4.386 D and a slight change in its direction. However, when this methyl group is introduced into 5-CU, the M5CU molecule, its dipole moment increases to 4.549 D with a remarkably change in its direction. However, when it is inserted into 6-CU, the M6CU molecule, remarkably decreases to the low value of 3.088 D with a slight shift in the direction of its dipole vector. The polarizability is also decreased as compared to uracil and 3MU, which reduces intermolecular interactions.

### 2.3. Geometry Optimization in the Solid State

**Figure 4:**
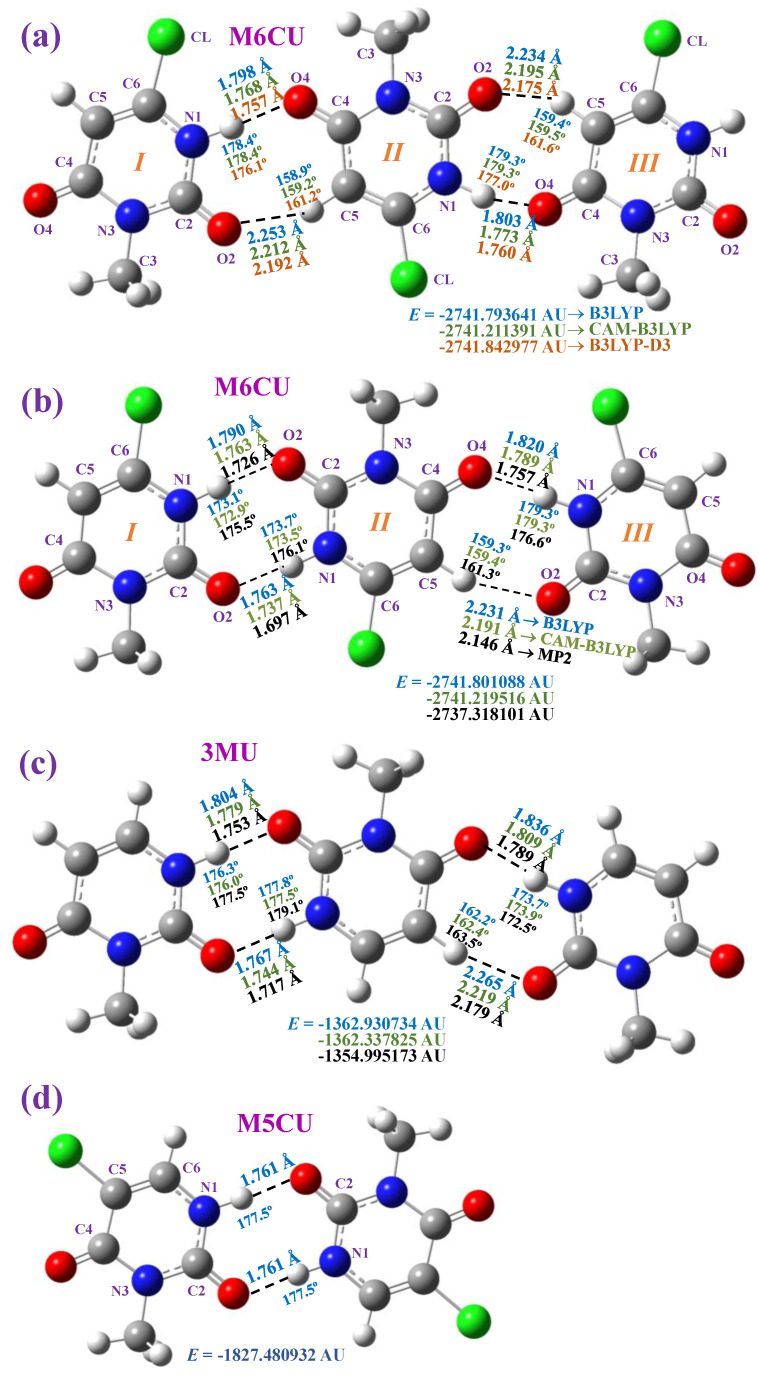
(**a**,**b**) Two optimized possible arrangements in the crystal unit cell of M6CU. (**a**) *A*-form: with two N1-H···O2 intermolecular H-bonds between *I* and *II* rings. (**b**) *B*-form: with the N1-H···O2 H-bond between *I* and *II* rings, and with C5-H···O2 between *II* and *III* rings. It is the trimer form most stable. (**c**) Optimized trimer form calculated at three levels in 3MU. The values included by the B3LYP (in blue), CAM-B3LYP (in grey) and B3LYP-D3 (in garnet) were with the 6-311++G(3df,pd) basis set, while those by MP2 (in black) were with 6-31G(d,p). (**d**) Optimized dimer form in M5CU at the B3LYP/6-31G(d,p) level.

With the aim of obtaining accurate wavenumbers (where MP2 fails remarkably [44]), the crystal unit cell reported in the solid-state sample by Gerhardt & Egert [26] was optimized by a simple approximation using a trimer form that, for convenience, was called the *A*-form, Figure 4a. This *A*-form was optimized at three DFT levels, and the geometric parameters obtained for ring *II* are presented in Table 1. Through this optimization, the calculated geometric parameters are expected to be closer to those obtained by X-ray analysis than to those calculated for a monomer form. In this trimer, the same N1-H···O4 and C5-H···O2 intermolecular H-bonds are established between the rings labeled as *I* and *II*, and between the rings *II* and *III*, and so on, forming long ribbons. It is necessary to point out that the objective with the trimer optimization is to obtain a better model in the wavenumber calculations for the interpretation of the vibrational spectra in the solid-state sample.

The optimized geometrical parameters involving these intermolecular H-bonds are included in Figure 4a by two DFT methods (including the dispersion correction D3 with B3LYP) with the 6-311++G(3df,pd) basis set, as well as the total electronic energy of this trimer system. The trimer is fully planar with the exception of the methyl hydrogens. It should be noted that the H-bond with the C5-H hydrogen is very weak as indicated by both the H5···O2 distance (around 2.23–2.25 Å by B3LYP, 2.19–2.21 Å by CAM-B3LYP, and 2.19–2.17 Å by B3LYP-D3) and the C5-H···O2 angle ca. 160°. However, this H-bond is remarkably weaker in the X-ray structure [26] with a H5···O2 distance of 2.41 Å and an angle of 156°. Another N1-H···O4 H-bond is also noticeably weaker in the X-ray structure with an average distance of 1.91 Å and an angle of 171°. Therefore, the shortest H-bond values calculated by dispersion correction D3 appear as the worst in this comparison.

These weaker values obtained by X-ray analysis could be explained by weaker CL···CL interactions that stabilize the parallel chains formed through two inversion-symmetric CL atoms, which helps to form two-dimensional layers [26]. But for the experimental two-dimension layer formation, some flexibility of the chain is necessary, i.e., a lengthening of the H-bonds and a distortion of their angles.

In general, this *A*-form can be characterized by long ribbons, but the rings are bonded by an intermolecular N-H···O H-bond and a very weak C-H···O interaction. This *A*-form exhibits a large dipole moment, which facilitates its water solution.

Another possible arrangement of the M6CU molecules in the trimer was optimized, called the *B*-form, Figure 4b. Two N1-H···O2 intermolecular H-bonds are established between the rings labeled as *I* and *II*, and another two different N1-H···O4 and C5-H···O2 intermolecular H-bonds between the rings *II* and *III*. This arrangement corresponds to the rotation of rings *II* and *III*, establishing now only one weak intermolecular C-H···O H-bond in the trimer. Consequently, this optimized trimer was calculated to be 19.5 and 21.3 kJ/mol by B3LYP and CAM-B3LYP, respectively (18.7 and 20.6 kJ/mol with the ZPE correction), more stable than that of Figure 4a, while the difference was 19.6 and 18.7 kJ/mol by CAM-B3LYP and MP2, respectively. Due to its higher stability, the optimized bond lengths and angles of ring *II* are included in Table 1, and the NBO charges in Table 2. This trimer was also optimized by the MP2 ab initio method. Other possible combinations of the intermolecular H-bonds among the three molecules of the trimer were studied, but only these two trimer forms were stable and fully agreed with X-ray diffraction results.

The energy associated with the trimer stability of these molecules was determined and included in Table 4 where, for convenience, “A” corresponds to the monomer with ring *I*, “B” to ring *II*, “C” to ring *III*, and A-C to these three monomers of the trimer. It should be noted that the energy values obtained by CAM-B3LYP are larger than by B3LYP but noticeably lower than those obtained by MP2, which provides the most reliable values. The B3LYP-D3 values are also larger than by CAM-B3LYP. As expected, the interaction energies *E*^int^ are noticeably larger in *A*-form than in *B*-form, with the deformation energies *E*^def^ greater in ring *II* (with intermolecular H-bonds on both sides of the molecule) than in ring *I*. The lowest values by all methods correspond to ring *III*, which exhibits the weakest intermolecular H-bond with ring *II*.

For comparison purposes, the optimized trimer with 3MU was optimized, Figure 4c. Because of the special characteristics of the *B*-form trimer, the arrangement of 3MU was adopted from the *B*-form, which was the most stable. However, this arrangement differs from that experimentally reported by X-ray [41], which revealed that only in the N1-H···O4 does an intermolecular H-bond appear between the molecules. Although the optimized trimer with this arrangement appears as the least stable, its stability in the crystal could be explained by considering other weak H-bonds/interactions in a 3D net.

In the *B*-form trimer of 3MU, rings *I* and *II* form slightly longer H-bonds than in M6CU, but with better orientation with a more open N-H···O angle of 177–179° by MP2. Similarly, rings *II* and *III* also appear with longer H-bonds than with M6CU and with a slightly closer N-H···O angle and more open C-H···O. As a result, the rings of this trimer appear with a slightly weaker H-bond than the corresponding M6CU of the *B*-form.

Comparing M6CU with 3MU, the chlorine atom at the sixth position of M6CU appears to stretch the trimer structure through these H-bonds, and this feature is also expected to occur with the *A*-form trimer. However, by the interaction energy values the molecules in the 3MU trimer appear to be slightly more strongly bonded (higher E^int^) than in M6CU. The opening of the C-H···O angle facilitates this interaction.

In M5CU, trimer formation was not possible due to the steric hindrance of the chlorine atoms; therefore, the dimer form was optimized, Figure 4d. The rings in this dimer appear to be significantly more strongly bonded (higher E^int^ values) than those observed for M6CU in Figure 4b, which is due to stronger intermolecular H-bonds and more open N1-H···O2 angles in M5CU than in M6CU.

### 2.4. Several Molecular Properties

Several properties such as thermodynamic parameters, rotational constants and heat capacity at constant volume (C_v_) were also computed in the compounds under study. These properties, together with the total energies, including the ZPE (zero-point vibrational energies) correction, are presented in Table 3 and Table 5.

Due to the large asymmetry of the molecules under study, the rotational constants in A, B and C differ significantly. In the monomer form, the values in the uracil molecule are remarkably higher than those in 3MU. This noticeable reduction caused by the methyl group effect becomes even greater in the presence of the chlorine atom and, therefore, in M6CU due to the combined effects of the methyl and chlorine substituents, resulting in very low rotational constant values. The calculated values in M6CU are almost identical across the different computational levels used, even at MP2. This result further confirms once more the accuracy of the DFT methods used in the present work. In the trimer form, the rotational constant values appear noticeably decreased to very small and almost-zero values.

The unscaled calculated entropies are also presented in Table 3 and Table 5. In the monomer form, the B3LYP value appears slightly larger than the M06-2X value, mainly because of its vibrational contribution, and it markedly increases with system complexity, as observed in the trimer form. This large increase in the trimer is mainly due to the increment in the vibrational contribution, which represents the major contribution to the total entropy, although the rotational and translational contributions also increase substantially.

The calculated heat capacity values at constant volume (C_v_) are similar between the DFT methods used, with the B3LYP values being slightly higher than those obtained with M06-2X and CAM-B3LYP methods and decreasing slightly as the basis set increases. The C_v_ value increases with increasing molecular size of the system or its complexity; therefore, 3MU and 6-CU have larger values than the uracil molecule, and 6MU has the largest one. The value in the trimer system is more than three times that of the corresponding monomers.

### 2.5. HOMO and LUMO Molecular Orbitals

The calculated HOMO (Highest Occupied Molecular Orbital) and LUMO (Lowest Unoccupied Molecular Orbital) energies for the studied molecules are presented in Table 6 using DFT methods and the 6-311++G(3df,pd) basis set, while Table S4 lists the values at other levels. The HOMO behaves as an electron donor, while the LUMO acts as an electron acceptor. The different computational methods yielded almost identical HOMO energies, and a similar feature was observed for the LUMO.

The HOMO–LUMO energy gap (E_g_) between these orbitals helps to identify and analyze the chemical reactivity and kinetic stability of the studied molecules and it is an essential and characteristic parameter in quantum chemistry [45], e.g., its value can explain the possible charge transfer within the molecule, which affects its chemical and biological activity. A reduction in the E_g_ value facilitates intramolecular charge transfer in the molecule (it becomes more polarizable), requiring less excitation energy, which is generally associated with increased reactivity, as well as, for example, nonlinear optical (NLO) activity.

The E_g_ value slightly decreases with increasing molecular complexity or system size. Therefore, its calculated value in the monomer form of 6-CU (5.59 eV) is slightly lower than that of uracil (5.63 eV) and that in M6CU (5.61 eV). However, the methyl group produces a small increase, as observed with 3MU and M6CU, which indicates that the methyl group has only a minor effect on the molecular reactivity. The lowest E_g_ value in 6-CU and M6CU indicates greater intramolecular charge transfer and higher reactivity than in 3MU and uracil molecules. The decrease becomes significantly greater for the trimer calculated by B3LYP (4.73 eV in A-form and 5.01 eV in B-form) vs. 5.61 eV in the monomer of M6CU. The decrease in the E_g_ value is also more pronounced with increasing basis-set size.

Using the calculated values of the HOMO and LUMO, the global chemical reactivity descriptors can be easily determined in a first approximation utilizing the following well-known general equations according to Koopmans’ theorem approach:

IP ≈ −E_HOMO_

EA ≈ −E_LUMO_

χ = −(E_HOMO_ + E_LUMO_)/2

η = (E_LUMO_ − E_HOMO_)/2

S = 1/η (or by 1/(2η) by other authors)

The ionization potential (IP) reaches its highest value for 6-CU (7.50 eV), followed by M6CU. The lowest IP value corresponds to 3MU, as expected. This value decreases with increasing system complexity, dropping from 7.35 eV in the monomer to 6.97 eV in the M6CU trimer of the B-form by B3LYP.

The electron affinity (EA) values are significantly lower than the corresponding IP values, and in contrast to IP, its value increases with increasing structural complexity. For example, the EA value obtained by B3LYP is 1.74 eV for the monomer and 1.96 eV for the trimer in the B-form.

The electronegativity (χ) values are very small in accordance with neutral molecules and slightly increase as the complexity decreases. The value is 4.46 eV in the trimer and 4.54 eV in the monomer. The chemical hardness (η) values, as well as the global softness (S), reveal the resistance of a system to a change in its number of electrons. Their values are low in the studied compounds and similarly to χ, their values slightly increase as the system complexity decreases. Due to these low values, the molecules under study can be classified as soft species, characterized by small energy gaps and easily modified electron densities.

### 2.6. Vibrational Wavenumbers

The detailed spectroscopic characterization of a compound (in particular, the M6CU drug) is useful for confirming its identity during synthesis by simple comparison of the observed IR/Raman spectra with that predicted or for detecting the formation of a completely new molecule. Therefore, this spectroscopic characterization is another aim of the present manuscript together with the effect of the chlorine atom and methyl group in the IR and Raman spectra by comparison with the spectra of uracil, 6-CU, and 3MU. Because of its importance, wavenumbers were calculated in the present study utilizing the simple harmonic oscillator model and, as a result, the computed values were always larger than the experimental ones. Therefore, due to deficiencies in the quantum-chemical method used, further corrections to the calculated wavenumbers, i.e., scaling factors, are required. For their accuracy, the B3LYP, CAM-B3LYP and M06-2X DFT methods were mainly used, as well as the B3LYP-D3 DFT method. However, with this last method the scaled wavenumbers for M6CU were very similar to those obtained using B3LYP and, for this reason, their results were omitted in the figures and tables of the present manuscript. The MP2 wavenumbers were not included either because their calculated values were always reported as worse than those by B3LYP [44].

#### 2.6.1. Procedures for Improving Wavenumbers: Scaling

Despite the great advances currently in hardware and theoretical calculation methods for molecules, especially in the use of DFT methods, a large overestimation of their calculated vibrational wavenumbers can be expected [44,46,47]. This systematic overestimation is due to several factors that are not usually included in theoretical approaches, but it can be considerably reduced by using a procedure to scale the computed wavenumbers. Several of these procedures have been described in our previous study [44,46,47].

The procedure chosen for scaling depends on both the organic molecule size to be studied and the required accuracy for its wavenumbers. Among the several possible approaches, the simplest one for scaling wavenumbers is to use an overall scale factor [48], but in several vibrations this procedure introduces a large error in their scaled values and, therefore, hinders a correct and reliable assignment of experimental bands. Thus, to reduce this error and obtain a more reliable assignment, four accurate scaling procedures are recommended to be used [44] for an uracil derivative such as M6CU. These procedures are the following: the linear scaling equation (LSE), the two linear scaling equations (TLSEs), the polynomic scaling equation (PSE), and the specific scale factors for each mode (SCFEM).

The equations and specific factors used in these procedures were determined from calculations performed at the same theoretical level with simpler and parent molecules that in the present work correspond to the uracil molecule [44]. For simplicity, only the equations used in the TLSE and PSE procedures are shown, which were the following [49]:

With TLSE for the 0–1500 cm^−1^ range, the equations were the following:

ν^scal^ = 3.3 + 0.9813 ν^cal^ (r = 0.9990) at B3LYP/6-31G(d,p) level

ν^scal^ = 5.7 + 0.9670·ν^cal^ (r = 0.9990) at M06-2X/6-31G(d,p) level

and for the 1500–4000 cm^−1^ range:

ν^scal^ = 34.7 + 0.9429 ν^cal^ (r = 0.9998) at B3LYP/6-31G(d,p) level

ν^scal^ = −51.4 + 0.9633·ν^cal^ (r = 0.9996) at M06-2X/6-31G(d,p) level

With PSE, the equations were [47]:

ν^scal^ = 3.2 + 0.9904·ν^cal^ − 1.12 × 10^−5^ (ν^cal^)^2^ by B3LYP/6-31G(d,p)

ν^scal^ = 17.3 + 0.9506·ν^cal^ − 0.23 × 10^−5^ (ν^cal^)^2^ by M06-2X/6-31G(d,p)

For the trimer with the 6-311++G(3df,pd) basis set, the equations were:

ν^scal^ = −6.8 + 1.0076·ν^cal^ − 1.38 × 10^−5^ (ν^cal^)^2^ by B3LYP/6-311++G(3df,pd)

ν^scal^ = −10.6 + 0.9904·ν^cal^ − 0.955 × 10^−5^ (ν^cal^)^2^ by CAM-B3LYP/6-311++G(3df,pd)

ν^scal^ = −6.1 + 1.0061·ν^cal^ − 1.341 × 10^−5^ (ν^cal^)^2^ by B3LYP-D3/6-311++G(3df,pd)

where ν^scal^ corresponds to the scaled wavenumbers and ·ν^cal^ to the calculated ones.

#### 2.6.2. Computed Wavenumbers of M6CU

##### In the Gas Phase (Isolated State)

The wavenumbers for this isolated state (monomer form) of M6CU were first obtained at two theoretical levels, and their values are included in Table 7. The first five columns correspond to the calculated values at the B3LYP/6-311++G(3df,pd) level, while the values at the B3LYP/6-31G(d,p) level are included in Table S5 (Supplementary Materials Section). The first column lists the computed wavenumbers, while their relative infrared intensities (A, %) are shown in the second column, and the relative Raman intensities (S, %) in the third column. Raman depolarization ratios, which indicate if a vibration is symmetric (polarized, P) or unpolarized (U), are collected in the fourth–fifth columns, respectively, while the force constants (f) of each vibration are listed, for simplicity, only in the sixth column of Table S5. The results determined at the M06-2X/6-31G(d,p) level are shown in columns 6–8. The scaled wavenumbers by the B3LYP and M06-2X methods were determined using the TLSE (two linear scaling equation) procedure, columns 9–10. This procedure was firstly selected for its slightly better accuracy than the PSE [44]. The last column corresponds to the characterization performed at the B3LYP/6-311++G(3df,pd) level for each computed wavenumber, including the %PED (Potential Electron Distribution) contribution of the different normal modes to each vibration. Contributions lower than 10% were not included. Similar characterization was performed at the B3LYP/6-31G(d,p) level, Table S5. The main discrepancy between both levels appears in the %PED. In this characterization, the notation used for the uracil ring normal modes was done following Ref. [46], and the corresponding ring number was included in the previous column.

The predicted scaled Raman spectrum in gas phase (monomer form) obtained by the B3LYP and M06-2X methods is plotted in Figure S1a, while the corresponding IR spectrum is shown in Figure S2a. Unfortunately, the experimental spectra in the gas phase or in the argon matrix have not been reported yet. Therefore, these theoretical spectra can only be compared to the experimental spectra in the solid state, which is also included in these figures. For a clearer comparison and assignment of the bands, the whole spectrum was divided in three ranges. For the Raman spectrum these correspond to 3600–2800, 1800–1000 and 1000–0 cm^−1^, Figures S3–S5, respectively.

Many bands in these scaled Raman spectra calculated using the B3LYP method with the 6-31G(d,p) and 6-311++G(3df,pd) basis set differ from those observed experimentally, i.e., new bands are predicted theoretically in the isolated state that have no correspondence in the experimental spectrum, and vice versa. The wavenumber position in many bands also differs from the experimental ones and the increase in the basis set to the large 6-311++G(3df,pd) does not lead to an improvement. This feature was also observed in the IR spectra, Figures S6–S8.

**Figure 5:**
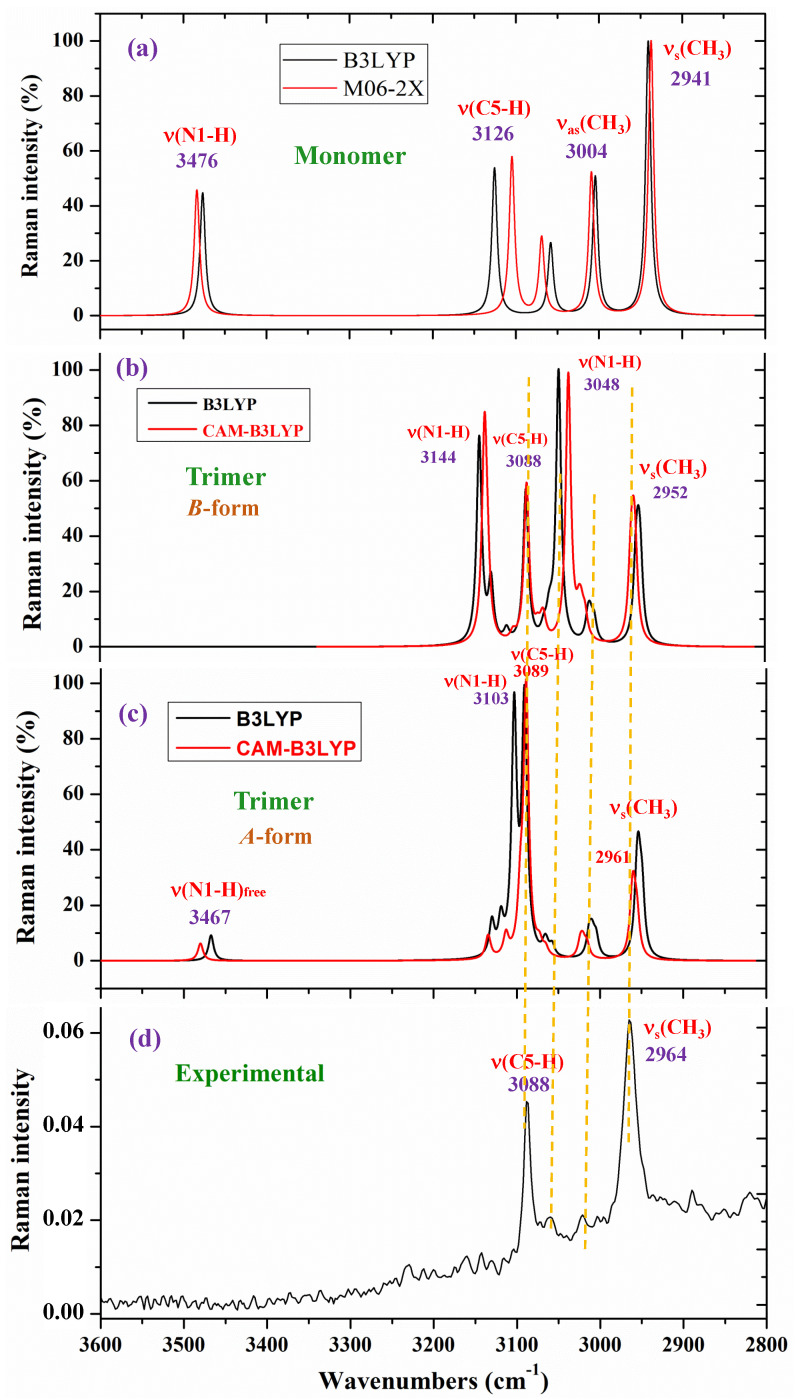
Comparison of the theoretical scaled Raman spectra by two DFT methods in the 3600–2800 cm^−1^ range: (**a**) for the monomer form and (**b**,**c**) for two trimer forms of M6CU using the polynomic scaling procedure (PSE) versus (**d**) the experimental one. The most intense bands with the main assignment are also included. The scaled wavenumbers in violet color correspond to B3LYP, while those in red color correspond to CAM-B3LYP.

**Figure 6:**
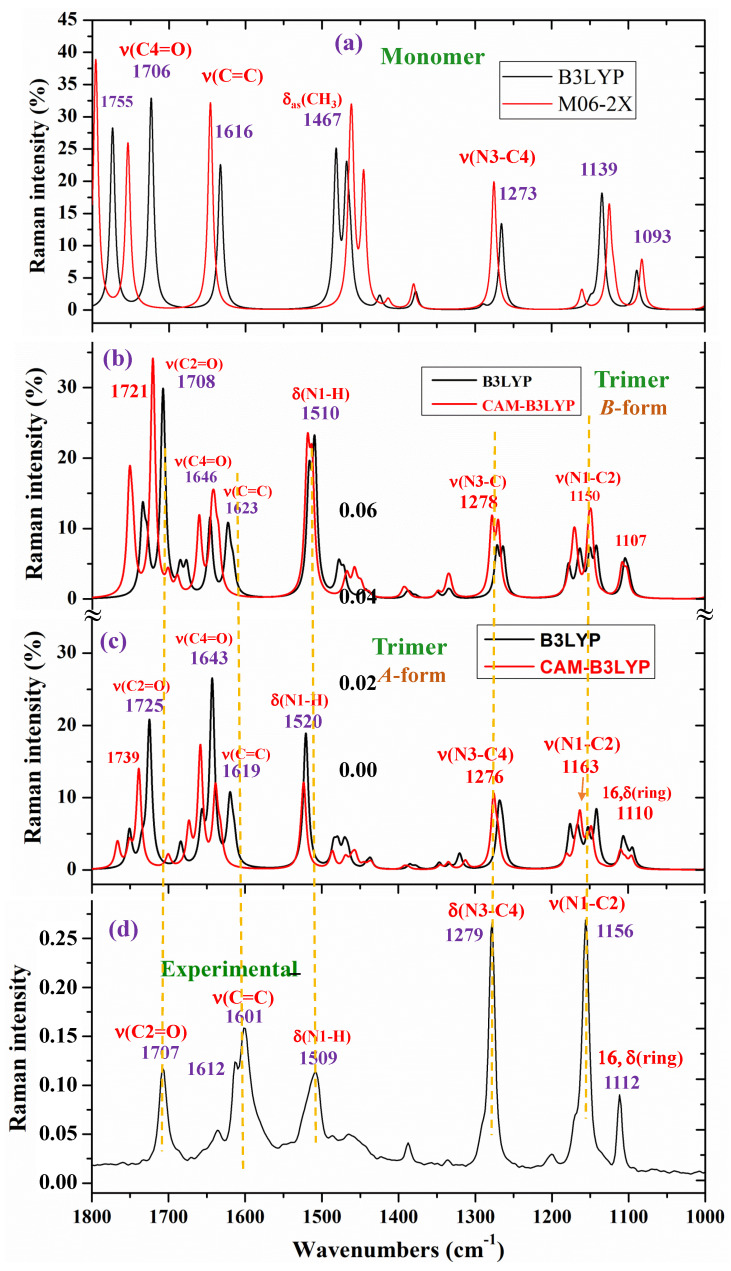
Comparison of the theoretical scaled Raman spectra in the 1800–1000 cm^−1^ range corresponding to: (**a**) the monomer form and (**b**,**c**) the trimer forms of M6CU using the polynomic scaling procedure (PSE) versus (**d**) the experimental one. The most intense bands with the main assignment are also included. The scaled wavenumbers in violet color correspond to B3LYP, while those in red color correspond to CAM-B3LYP.

**Figure 7:**
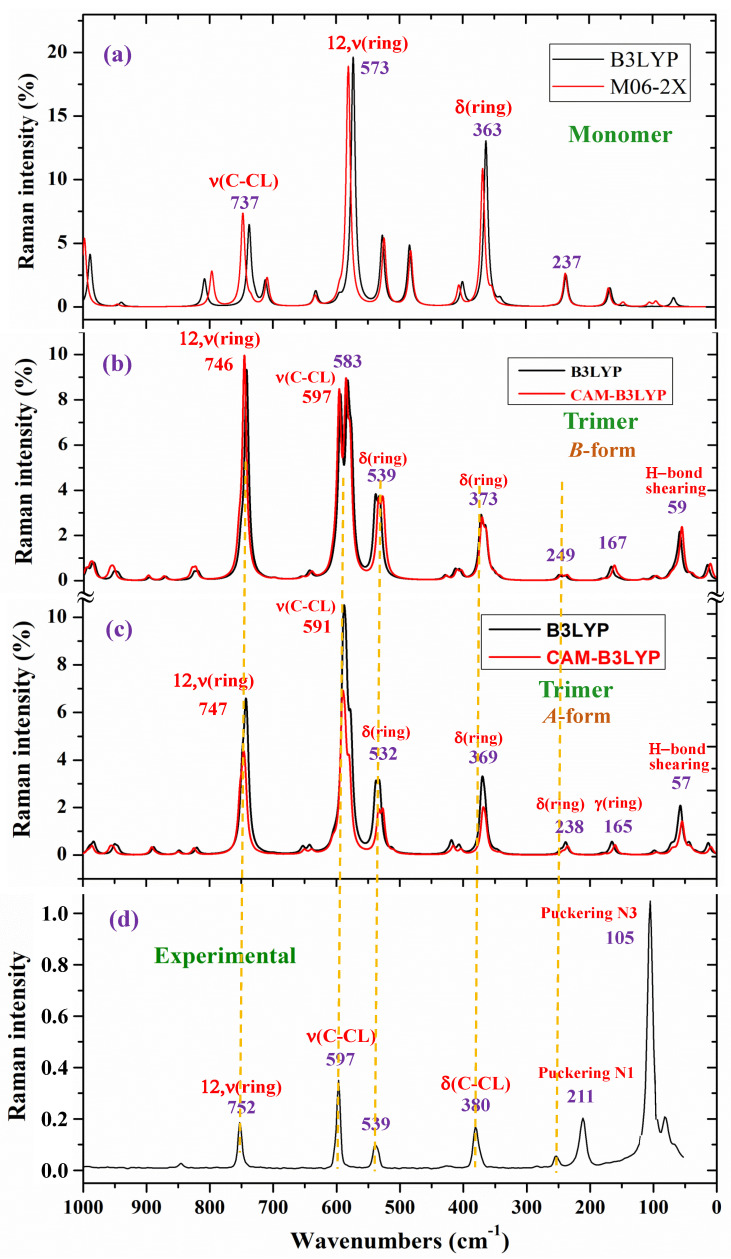
Comparison of the theoretical scaled Raman spectra in the 1000–0 cm^−1^ range corresponding to: (**a**) the monomer form and (**b**,**c**) the trimer forms of M6CU using the polynomic scaling procedure (PSE) versus (**d**) the experimental one. The most intense bands with the main assignment are also included. The scaled wavenumbers in violet color correspond to B3LYP, while those in red color correspond to CAM-B3LYP.

To overcome this limitation with the Raman spectra, one of the possibilities was to calculate and scale the spectra using the M06-2X DFT method and its further comparison to those in the monomer form with B3LYP and to the experimental ones, Figure 5, Figure 6 and Figure 7. However, this comparison revealed a worsening by M06-2X related to B3LYP. Although both scaled spectra appear similar, with different wavenumber shifts, a large discrepancy is observed in the modes involved in intermolecular H-bonds in the solid-state crystal as compared to the experimental ones.

**Figure 8:**
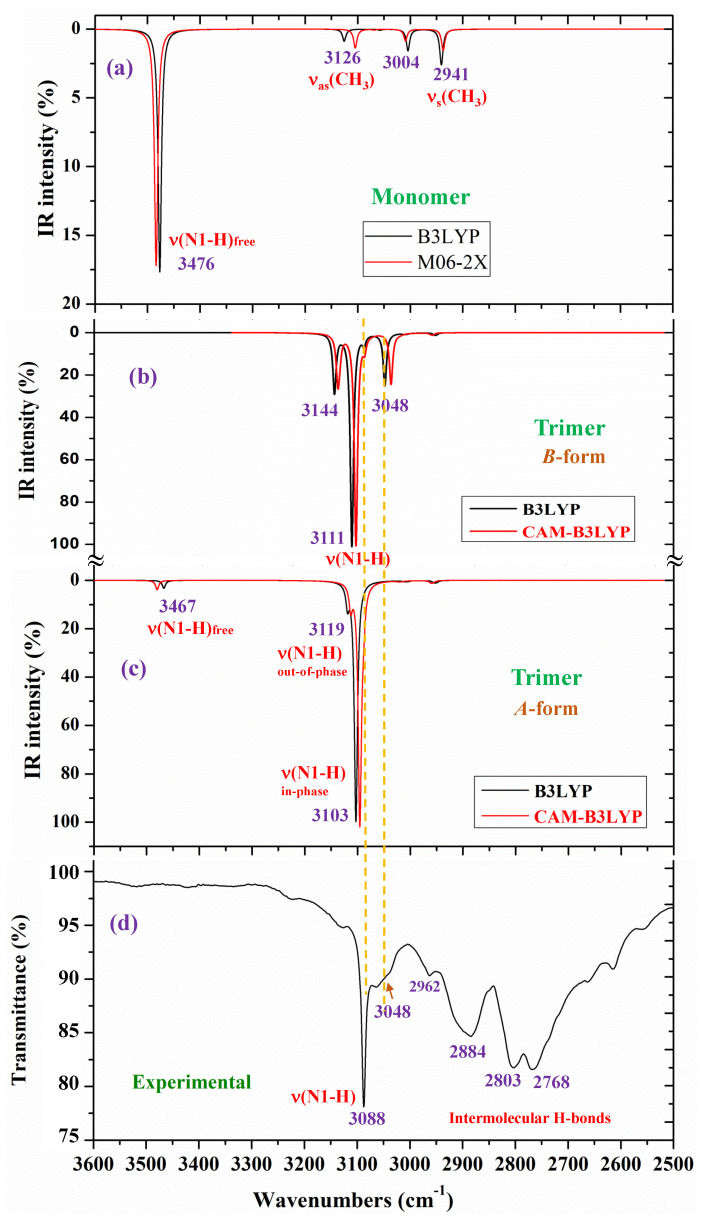
Comparison of the theoretical scaled IR spectra in the 3600–2500 cm^−1^ range corresponding to: (**a**) the monomer form and (**b**,**c**) the trimer forms of M6CU using the polynomic scaling procedure (PSE) versus (**d**) the experimental one. The most intense bands with the main assignment are also included.

All these disappointments were also observed in the IR spectra. For example, the scaled wavenumber at 3476 cm^−1^ calculated by B3LYP in the IR spectrum of the monomer and corresponding to the ν(N1-H) stretching (Figure 8a) has no counterpart in the experimental spectrum (Figure 8d), nor in its Raman spectrum, Figure 5d. Similar discrepancies are observed in other bands. The M06-2X method also failed to improve the agreement between the monomer spectra and the experimental spectra in the solid state.

These results indicate that the predicted vibrational spectra in the gas phase (monomer form), with all their bands assigned, can only serve as a reference for future experimental IR/Raman spectra in the gas phase or in an argon matrix spectrum of M6CU. The crystal packing forces present in the solid-state sample have considerable importance in their spectra, as in the present M6CU molecule and, therefore, the theoretical model was improved with a trimer form for achieved a better interpretation of the experimental bands in the solid state, as described in the next sections.

##### In the Solid State (Trimer Form)

For an accurate assignment of the experimental spectra in the solid-state sample it is necessary to reproduce their present intermolecular H-bonds, and this can be achieved by improving the theoretical model. The simplest way to do this is by the simulation of the crystal unit cell in a trimer form, as described before, with the corresponding calculation of the IR and Raman spectra, Figure S1 and Figure S2, respectively. Therefore, the scaled frequencies of these theoretical spectra can now be unequivocally compared with experimental ones.

The vibrational spectra of the two possible trimer forms were calculated with the B3LYP, CAM-B3LYP and B3LYP-D3 methods using the 6-311++G(3df,pd) basis set and subsequently compared to the experimental IR and Raman ones. Because the Raman spectrum obtained by B3LYP-D3 (Figure S9) was very close to that obtained using B3LYP, as well as the IR spectrum (Figure S10), the results obtained by B3LYP-D3 were omitted in the discussion of the results obtained.

The first step in this study was to determine which of the two possible trimer forms is the one expected in the solid state of our sample. Although the *A*-form is that reported by X-ray, it is calculated to be less stable than the *B*-form, and it is necessary to confirm this feature. For this purpose, a first comparison of the Raman bands in the red region of the *A*-form (Figure 5c) and the *B*-form trimer (Figure 5b) was carried out with those of the experimental Raman spectrum in Figure 5d. To facilitate comparison, orange vertical dashed lines were drawn in all spectra figures.

Therefore, it was noted that three bands with strong and very strong intensities were determined by B3LYP and four bands by CAM-B3LYP, while only two Raman bands were clearly identified in the experimental spectrum at 3088 and 2964 cm^−1^. However, in the *A*-form trimer (Figure 5c) two Raman bands are clearly distinguishable in the spectra and with wavenumbers very close to the experimental values. Because of this large discrepancy between the *B*-form and the experimental spectrum in the N1-H and C-H stretching wavenumbers, as well as in a few other bands, this *B*-form was considered slightly less representative, and the *A*-trimer form was considered as that mainly predominated in the solid state of the sample. The detailed assignment and analysis of the spectral bands was carried out focused on the spectra of this *A*-form trimer. The use of B3LYP without and with dispersion corrections (D3) using the 6-311++G(3df,pd) basis set yielded close values, and for this reason, the spectra using this B3LYP-D3 method were not considered. This feature was also found by other authors [50].

However, although most of the experimental bands correspond well to this *A*-form trimer, a few of them are reproduced more accurately with the intermolecular H-bond arrangement of the *B*-form, which indicates that a very small number of the H-bonds may correspond to those of the *B*-form. The predicted wavenumbers using the B3LYP method are generally very close to those obtained by CAM-B3LYP and in very good agreement with the experimental spectra, especially with the Raman spectrum. Comparing the accuracy of both methods vs. the experimental results, it was noted that in general CAM-B3LYP tends to predict wavenumbers closer to experimental values in the red region, while B3LYP performs better in the blue region of the spectrum.

The largest discrepancy between theory and experiment appears in the 3600–2500 cm^−1^ range of the IR spectrum where three broad absorptions at 2884, 2803 and 2768 cm^−1^ (Figure 8d) have no correspondence with the predicted theoretical spectra. Based on their broad intensity and wavenumber positions they can be assigned to intermolecular H-bonds in the solid-state crystal that were not simulated in our simplified trimer model. In the 3100–2600 cm^−1^ range of the experimental Raman spectrum of Figure S11, very weak lines indicated by arrows were observed and, if they are not due to background noise, they may correspond to intermolecular H-bond stretching modes. A similar feature was observed in the experimental IR spectrum of 6-CU [17], although they were assigned to overtones and combination bands.

#### 2.6.3. Description of the Different Normal Modes in the Trimer Form of M6CU

Three wavenumbers appear for each vibration in the optimized trimer systems, which were collected only for the *A*-form in Table 8 and Table 9 using both the B3LYP and CAM-B3LYP methods. The results presented in Tables S7 and S8 correspond to the *B*-form using the CAM-B3LYP method, which were similar to the *A*-form. For simplicity, only the scaled values were included in these Tables and Figures. Among these three wavenumbers, special attention was given to the modes corresponding to the central molecule of the trimer, i.e., ring *II*.

This is because ring *II* is involved in intermolecular H-bonds on both sides with rings *I* and *III*; therefore, their vibrational modes correspond more closely to those found experimentally in the solid-state crystal. Since the molecules of the *A*-form trimer exhibit relatively weak intermolecular H-bonds, many vibrational modes are not coupled between the molecules of the trimer, which facilitates the assignment.

Therefore, the wavenumbers of lesser interest corresponding to rings *I* and *III* were easily identified and marked with the symbol (*) in Table 8 and Table 9, and they were not considered in the discussion. Among these three wavenumbers, the one with the highest IR intensity is shown in bold, whereas the one with the highest Raman intensity is shown in *italics*.

The IR and Raman intensities of these wavenumbers are included in the second–third columns of Table 8 for B3LYP and in the fifth–sixth columns for the CAM-B3LYP method. The next two columns list the experimental wavenumbers corresponding to the IR and Raman spectral bands of the M6CU sample in the solid state, along with their respective intensities. The final two columns show the most relevant theoretical characterization of these calculated vibrations, including the normal mode numbers defined for the uracil ring [46].

In this characterization, the % PED values in parentheses correspond to the scaled wavenumber of ring *II* (without the * asterisk symbol) or to the wavenumber with the largest contribution from ring *II*. Contributions below 10% were omitted. Owing to the similarity of the scaled wavenumbers from the B3LYP and CAM-B3LYP methods and for simplicity, this characterization mainly corresponds to calculations performed at the B3LYP/6-311++G(3df,pd) level in both Table 8 and Table 9.

The assignments of all pyrimidine ring modes in M6CU fall within the expected ranges reported in the literature for uracil and related derivatives [17,29,46,49]. Although Table 8 and Table 9, and Tables S5–S8, as well as the Raman and IR spectra plotted in Figure 5, Figure 6, Figure 7, Figure 8, Figure 9 and Figure 10 and in Figures S1–S8, may be self-explanatory, a brief discussion of several normal modes is described in detail as follows:

*C=O Vibrational Modes*

The carbonyl stretching motions in M6CU are significantly coupled with N-H bending modes, in agreement with those observed in the 6-chlorouracil molecule (6-CU) [17] and in other uracil derivatives [29,46]. Therefore, the ν(C2=O) stretching (mode **no. 26** of the uracil molecule [46]) appears to be strongly coupled with the ν(C4=O) and the in-plane bending δ(N1-H) modes. In the monomeric form it was calculated at 1787 cm^−1^ (scaled at 1750 cm^−1^) by B3LYP/6-311++G(3df,pd), very close to that of 3MU at 1783 cm^−1^ and to 6-CU at 1805 cm^−1^ (scaled at 1760 cm^−1^), and to uracil at 1801 cm^−1^ (scaled at 1763 cm^−1^), i.e., the wavenumber of mode **26** in the uracil ring appears little affected by the different substituents [29,46], with a small increase of ca. 4 cm^−1^ when the chlorine atom is inserted (6-CU), but a decrease of ca. 15 cm^−1^ when the methyl group is inserted into 3MU. The weak coupling between the C2=O and C4=O stretching modes in uracil molecule is slightly enhanced with chlorine atom insertion, and noticeably increased up to 20% PED with the methyl group is inserted in the third position.

**Figure 9:**
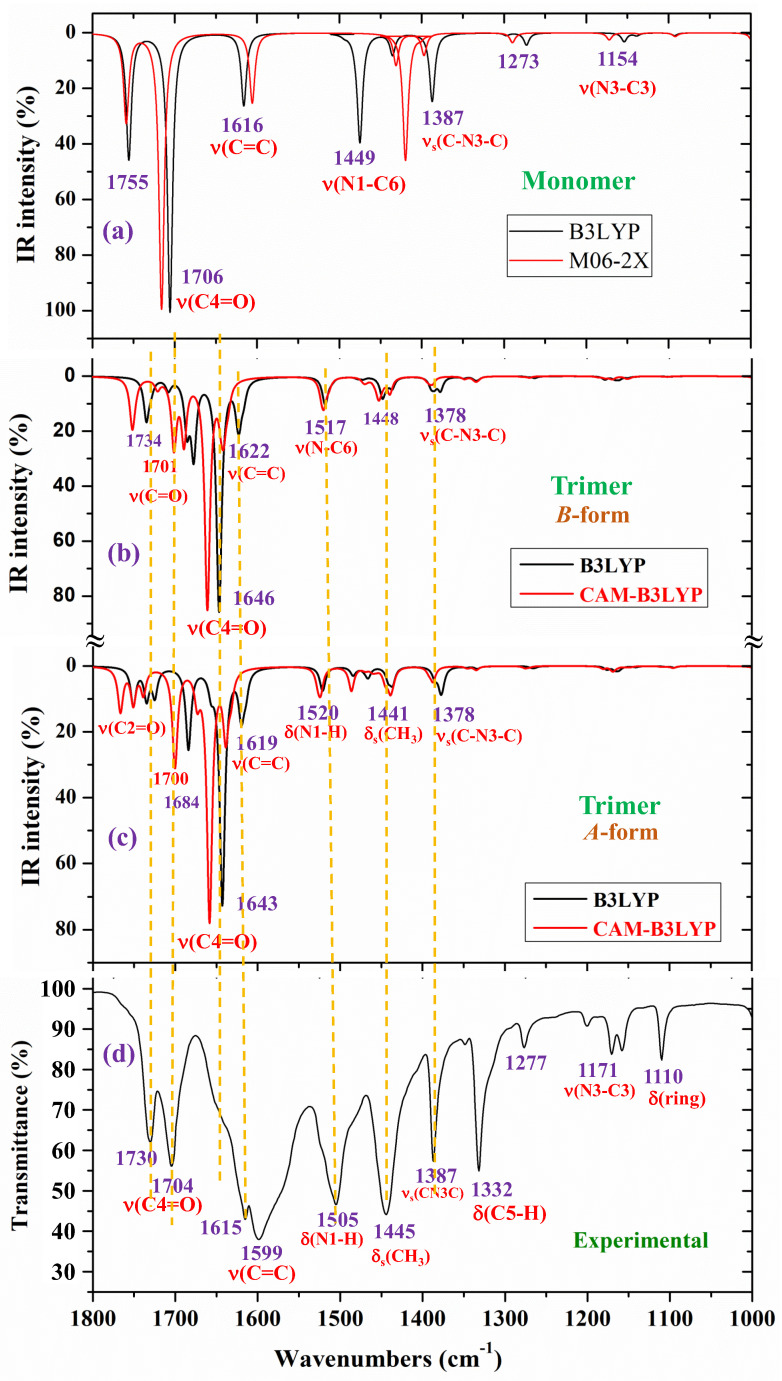
Comparison of the theoretical scaled IR spectra in the 1800–1000 cm^−1^ range corresponding to: (**a**) the monomer form and (**b**,**c**) to the trimer forms of M6CU using the polynomic scaling procedure (PSE) versus (**d**) the experimental one. The most intense bands with the main assignment are also included.

However, in the solid state, the different intermolecular H-bonds that can be established involving the C=O bonds are noticeably affected depending on the substituent, as observed by the wavenumber shift of this ν(C2=O) mode. Therefore, in M6CU it was assigned to the IR band at 1730 cm^−1^ (Figure 9d), close to that predicted by B3LYP at 1735 cm^−1^. For a better visual correspondence between the scaled and experimental bands, vertical dashed lines in orange were drawn.

The ν(C4=O) stretching (**no. 25**) appears to be strongly coupled to the ν(C2=O) stretching and δ(N1-H) bending modes, similarly to that found with the ν(C2=O) mode and to that found in 3MU. In the monomer form, its IR intensity was predicted to be remarkably higher than that of ν(C2=O), being the strongest in the spectrum. Its wavenumber appears little affected when the chlorine atom is inserted into the uracil molecule (calculated at 1768 cm^−1^ vs. at 1766 cm^−1^ in 6-CU), while it is noticeably red-shifted (ca. 35 cm^−1^) when the methyl group is inserted at 1732 cm^−1^ in 3MU and at 1731 cm^−1^ in M6CU.

In a similar way, in the trimer form its IR intensity was predicted to be higher than that of ν(C2=O), making it the second strongest band in the spectrum, and its wavenumber is noticeably shifted depending on the intermolecular H-bonds. The strong IR band at 1704 cm^−1^ was assigned to this mode, but our predictions with the scaled values correspond to 1643 cm^−1^ by B3LYP and 1660 cm^−1^ by CAM-B3LYP, and to a similar value by B3LYP-D3, Figure S12. This large shift may indicate that the intermolecular H-bonds in the solid state through the C4=O bonds are not as strong as those obtained in our simplified trimer model. In the Raman spectrum, this vibration was predicted at a lower wavenumber and with low intensity, which makes its identification difficult.

**Figure 10:**
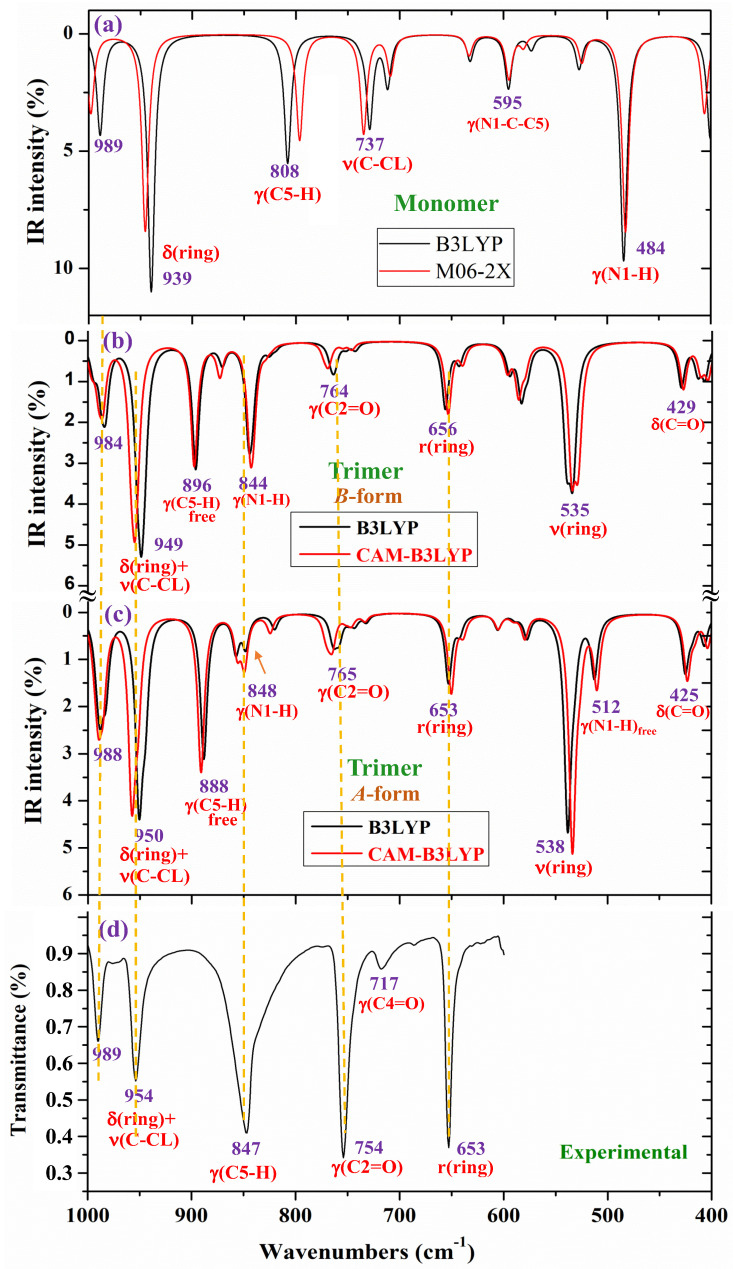
Comparison of the theoretical scaled IR spectra in the 1000–400 cm^−1^ range corresponding to: (**a**) the monomer form and (**b**,**c**) the trimer forms of M6CU using the polynomic scaling procedure (PSE) versus (**d**) the experimental one. The most intense bands with the main assignment are also included.

The in-plane bending δ(C=O) vibrational modes appear to be distributed among several modes, such as in the numbers **3**, **5**, **6** and **7**. The greatest contribution is observed in mode **3** and it was calculated with very weak and almost negligible Raman activity, along with a weak IR intensity. It was scaled at 425 (IR, Figure 10) and 407 (Raman) cm^−1^ by B3LYP (Table 9). This assignment is in accordance with the experimental data for 5-chlorouracil (5-CU), which shows bands at 446 cm^−1^ (IR) and 457 cm^−1^ (R), and for 6-CU at 451 cm^−1^, which exhibits a strong IR band and at 443 cm^−1^ a very weak Raman line [17]. In the uracil molecule, mode 3 was scaled at 383 cm^−1^.

The out-of-plane γ(C2=O) mode (**no. 11**) appears at a higher wavenumber and stronger IR and Raman intensities than the γ(C4=O) mode (**no. 10**) of uracil derivatives [29]. Therefore, γ(C2=O) appears at a higher wavenumber in M6CU, scaled in the trimer form at 765 cm^−1^ and in good agreement with the experimental IR band at 754 cm^−1^. This assignment also agrees with that found experimentally in the gas phase of the uracil molecule at 757 cm^−1^ [51,52], with that in 5-CU at 743 (IR) and 740 cm^−1^ (Raman), and in 6-CU at 758 cm^−1^ as IR band with medium intensity and at 723.3 cm^−1^ as a very weak Raman line [17].

In the monomer form of M6CU, γ(C4=O) mode (**no. 10**) was scaled at 716 cm^−1^ in accordance with the values found at 721 cm^−1^ in the uracil molecule and at 722 cm^−1^ in 6-ClU [17]. This is because in both M6CU and uracil molecules this mode appears very strongly coupled with the γ(C5-H) mode, which avoids its wavenumber change. However, a remarkable increase of 40–50 cm^−1^ is observed with the 5-substitution of the uracil molecule; therefore, in 5-ClU it was shifted at 775 cm^−1^ [17] due to its strong coupling with the γ(NCC=C) mode. In the trimer form of M6CU, this mode **10** was predicted at 719 cm^−1^ and assigned to the very weak experimental IR band at 717 cm^−1^ and to the extremely weak Raman line at 720 cm^−1^ in accordance with that reported in related molecules [17].

*N-H Vibrational Modes*

In uracil and its substituted derivatives, the N-H and C-H stretching modes are normally found in the 3500–2900 cm^−1^ range, but due to the N-H bond being shorter than the C-H bond, the N-H stretching modes are observed on the highest wavenumber side. These N-H and C-H modes are calculated as essentially pure modes (100% PED), and ring substitution produces only a small shift of ca. 1% or less [29]. Thus, the calculated and experimental wavenumbers in M6CU should be similar to those in the uracil molecule. Therefore, in the isolated state of M6CU, the ν(N1-H) stretching (**no. 30)** wavenumber was calculated at the B3LYP/6-311++G(3df,pd) level as pure mode and scaled at 3473 cm^−1^. This value is close to that scaled at the same level at 3478 cm^−1^ in 6-CU [17], and to that scaled at 3479 cm^−1^ in the uracil molecule and to its experimental one in the gas phase value at 3484 cm^−1^ as a strong IR band [51,52].

In the solid state (trimer form), the N1-H group participates in intermolecular H-bonds. Therefore, the stretch of these H-bonds leads to a noticeable shift of ν(N1-H) to a lower wavenumber appearing scaled by B3LYP at 3103 cm^−1^ and with the strongest IR and Raman intensity, in good agreement with the experimental IR of medium intensity at 3088 cm^−1^. The CAM-B3LYP method better predicts this very strong IR band at 3096 cm^−1^, but it is characterized as ν(C5-H) and strongly coupled with ν(N1-H) mode. The ν(N1-H)_free_ stretching mode in free molecules (without intermolecular H-bonds) was predicted with very weak IR intensity at 3467 cm^−1^ (by B3LYP) and at 3480 cm^−1^ (by CAM-B3LYP), values close to that found in the monomer form. Because of this very weak IR intensity or due to the lack of free N1-H groups in the solid state, it was not clearly detected in the experimental IR spectrum. However, in the experimental IR spectrum of 6-CU in the KBr matrix a very weak band was observed at 3439 cm^−1^ and assigned to this ν(N1-H) mode.

There are several vibrations (modes **16**, **19, 21** and **24**) to which the in-plane bending δ(N1-H) contributes significantly. However, the main contribution corresponds to mode no. **23**, which appears scaled in the isolated state at 1302 cm^−1^ and strongly coupled to the δ(C5-H) and δ(ring) modes. Because of the differences in this coupling, its wavenumber changes depending on the substitution. Thus, it was scaled at 1449 cm^−1^ in 6-CU and 1472 cm^−1^ in uracil.

In the trimer form, this δ(N1-H) bending vibration is also distributed among many modes, 16, 19, 21, 24 and 26, where it contributes significantly and extensively overlaps with other modes. In particular, mode **no. 23** involves contributions from the ν(N1-C6) and ν(C2=O) vibrations. In crystals with intermolecular H-bonds [29], this mode appears generally as a broad band and is shifted ca. 50 cm^−1^ to higher wavenumbers, consistent with our assignment to the experimentally observed very strong IR band at 1505 cm^−1^ and Raman line at 1509 cm^−1^. It is also in agreement with our scaled frequencies at 1520 cm^−1^ by B3LYP and 1523 cm^−1^ by CAM-B3LYP.

Out-of-plane bending γ(N1-H) corresponds to mode **no. 8**, and in the isolated state it was scaled at 486 cm^−1^ with a PED of 64% and strongly coupled with the γ(ring) mode. A similar wavenumber at 484 cm^−1^ was scaled in 6-ClU [17]. In the trimer form, with intermolecular H-bonds through the N1-H group, it shifts to a noticeably higher wavenumber, at 848 cm^−1^, in excellent accordance with the experimental strong IR band at 847 cm^−1^ (IR) or at 846 cm^−1^ (Raman). It also appears strongly coupled with the γ(C5-H) mode. In 6-CU, it was assigned to the experimental Raman line at 757.8 cm^−1^, while in 5-CU it was detected at 830 cm^−1^.

*C-CL Modes*

The C-Cl vibrations are strongly coupled with ring modes, making them difficult to identify. In the isolated state of M6CU, a large contribution from the stretching ν(C-Cl) mode was observed at the scaled value of 756 cm^−1^ and with a smaller contribution in the scaled value at 573 cm^−1^. In the trimer form it was mainly characterized at 589 cm^−1^ in good agreement with the very strong experimental Raman band at 597 cm^−1^. In the tetramer form of 6-CU [17], this mode was identified at 381 cm^−1^, which agrees well with the experimental Raman band at 384.6 cm^−1^. In halo-uracil derivatives [29], the wavenumber was found to decrease as the r(C-halogen) bond length increased.

The in-plane δ(C-Cl) bending vibration was predicted in the isolated state of M6CU at 363 cm^−1^, which is close to the scaled value in the trimer form at 371 cm^−1^ and shows good agreement with the experimentally observed strong Raman band at 380 cm^−1^. Another large contribution was predicted at 246 cm^−1^ and also in agreement with the weak Raman line at 254 cm^−1^. This vibration appears at a similar wavenumber to that found at 280 cm^−1^ in the tetramer form of 6-CU and corresponding to the experimental Raman line at 267.6 cm^−1^ [17].

The γ(C-Cl) out-of-plane bending mode was not clearly identified in M6CU, possibly because it is included in the puckering vibration predicted at 181 cm^−1^. In the tetramer form of 6-CU, this mode was scaled at 180 cm^−1^ and well related to the weak Raman line observed at 186.5 cm^−1^. In 5-CU, this mode was assigned to the Raman line at 125 cm^−1^.

*Ring Vibrations*

The stretching ν(C-C) and ν(C-N) normal modes of the uracil ring correspond to numbers **12**, **14**, **17**, **18**, **21** and **24** [46]. These modes were theoretically found to be strongly coupled to the N-H, C-H and C=O modes. The stretching vibration corresponding to the C5=C6 bond was identified in mode no. **24** and calculated in the isolated state of the M6CU molecule with strong IR and Raman intensities at 1626 cm^−1^, in agreement with the scaled reported value in 6-CU at 1619 cm^−1^ and at 1640 cm^−1^ in the uracil molecule.

This mode always appears strongly coupled with other normal ring modes. In the trimer form of M6CU, it was scaled at 1619 cm^−1^ in excellent accordance with the very strong experimental IR band at 1615 cm^−1^ and with the Raman line of medium intensity at 1612 cm^−1^. In the tetramer form of 6-CU, it was predicted at 1608 cm^−1^ and corresponding to the experimental bands at 1599 cm^−1^ (IR) and 1630 cm^−1^ (Raman), consistent with our calculated values.

The broad experimental band observed in the IR spectrum of M6CU at 1387 cm^−1^, as well as in 6-CU at 1414 cm^−1^, and assigned to the ν(C=C) mode 24, differs from our calculations. The methyl group causes a noticeable red-shift of ~40 cm^−1^ in this strong band. It may be explained by the strong coupling with N-H and C=O modes that are involved in strong intermolecular H-bonds in the solid state, where the strength of these H-bonds varies across different crystal regions. The three broad IR absorptions at 2884, 2803 and 2768 cm^−1^ with strong intensity observed in the experimental spectrum of M6CU support this feature. In the gas phase experimental spectrum of uracil molecules, this mode was assigned to the IR band observed at 1641 cm^−1^ [46,51,52].

Mode **21** is mainly characterized as ν(C2-N3-C4) in the uracil molecule at 1381 cm^−1^. This wavenumber appears close to that scaled at 1385 cm^−1^ in the ring *II* of the M6CU trimer form, although the most intense wavenumber is at 1378 cm^−1^ corresponding to ring *I*. This wavenumber is in excellent agreement with the experimental IR band with strong intensity observed at 1387 cm^−1^. The absence of the methyl group on the N3 nitrogen atom, as in 6-CU, increases its wavenumber; thus, the experimental bands at 1414 cm^−1^ (IR) and at 1406.6 cm^−1^ (Raman) were assigned to this mode [17].

Mode **18** is also characterized as C-N and in the trimer form of M6CU it appears in the scaled wavenumber at 1176 cm^−1^ with the main contribution from the N3-CH_3_ bond, showing weak IR and Raman intensities. This agrees well with the weak experimental IR and Raman bands at 1171 cm^−1^.

Mode **6** appears scaled in the trimer form of M6CU at 653 cm^−1^, matching the same wavenumber of the very strong experimental IR band, and of the extremely weak Raman line at 655 cm^−1^. In 6-CU, the weak experimental IR band at 620 cm^−1^ and the Raman line at 617.2 cm^−1^ could be related to this mode.

Mode **5** appears mainly characterized as δ_as_(C-N1-C) + δ_as_(N3-C4-C5) and in the trimer form of M6CU strongly coupled with the ν(C-Cl) stretching mode. In this trimer form, it was scaled at 589 cm^−1^ and related to the experimental Raman line at 597 cm^−1^. In the experimental gas phase of the uracil molecule, the IR band at 512 cm^−1^ corresponds to this mode [51,52].

Three out-of-plane vibrations, including torsional and puckering modes (nos. **4**, **2** and **1**) were scaled in the trimer form at 606, 181 and 99 cm^−1^, respectively, and the two latter modes were well related to the experimental Raman lines at 211 and 105 cm^−1^, respectively.

*Lattice Modes*

Nine lattice vibrational modes below 200 cm^−1^ and corresponding to the three molecules of the trimer form of M6CU were also identified and are included in Table 9. The notation used follows that reported for related molecules [17]. The presence of these nine distinct modes in M6CU indicates a high conformational flexibility in its molecular structure, which plays an essential role in its biological functions.

This considerable number and shape of low-frequency modes is consistent with the generally high flexibility of nucleobases and their derivatives. These modes may be key to local molecular recognition as they appear highly susceptible to changes in the local molecular environment, such as in its hydration and interaction (H-bonds) with other biomolecules.

*C5-H Modes*

In the isolated state of M6CU, the *v*(C5-H) stretching (**no. 28**) appears as a pure mode in the scaled value at 3131 cm^−1^, which agrees well with the scaled value at 3135 cm^−1^ in 6-CU. In the uracil molecule, this mode coupled to some extent with the ν(C6-H) mode, which slightly shifted its wavenumber to the scaled value at 3121 cm^−1^ [46]. In the trimer form, B3LYP predicts it at 3091 cm^−1^ with almost null IR intensity but with very strong Raman intensity. However, CAM-B3LYP scales it at 3096 cm^−1^ (IR) and 3089 cm^−1^ (Raman), but with the highest IR and Raman intensities and characterized it as ν(C5-H) strongly coupled with ν(N1-H).

These highest intensities and the close proximity of its wavenumber with that of the ν(N1-H) mode can lead to mistakes in the assignment of the experimental bands depending on the theoretical method used. Thus, through a detailed analysis of the bands and comparison with those reported for related uracil derivatives, the experimental IR band at 3088 cm^−1^ in M6CU was mainly assigned to N1-H stretching, while the Raman line at 3088 cm^−1^ was characterized as a C5-H stretching mode strongly coupled with the N1-H mode.

The in-plane bending δ(C5-H) mode appears strongly coupled with several modes, especially δ(N1-H), and distributed among modes nos. **16**–**18** and **23**. In the isolated state, the main contribution occurs in mode no. **23**. In the trimer form, the main contribution was found in mode **19**, scaled at 1347 cm^−1^ and corresponding to the experimental IR band at 1332 cm^−1^ and to the extremely weak Raman line at 1336 cm^−1^.

The out-of-plane γ(C5-H) mode (**no. 13**) was scaled at 826 cm^−1^ in the isolated state of M6CU and also strongly coupled with other ring modes, which agrees well with the scaled values found in 6-CU at 818 cm^−1^ and with uracil molecule at 810 cm^−1^. In the trimer form it was scaled with the highest intensity in ring *I* (where the C5-H group is free) at 888 cm^−1^ (Figure 10c), while its IR intensity is almost null in ring *II* at 858 cm^−1^. Due to this difference, and because the plotted scaled spectra correspond to all wavenumbers and not only those of ring *II* that are present in the long ribbons of the solid-state sample, the predicted bands at 888 cm^−1^ (*A*-form) and at 896 cm^−1^ (*B*-form) do not have correspondence with the experimental spectrum, as expected.

### 2.7. Effect of M6CU in Base-Pair Formation

Halogenation of uracil alters many of its properties such as its dipole moment, polarizability, acidity, etc. This modification can induce conformational changes in DNA/RNA helices by disrupting the Watson–Crick base pairing between the strands and weakening the strength of the stacking interactions between the base planes, that is, thereby affecting the helical parameters. Because many halogenated uracil derivatives have been used as potent antitumor and antiviral agents, we consider it of interest to study the effect of the chlorine atom (and also methyl group) in the structure and stability of the base pair.

It should be noted that nucleosides derived from modified nucleobases, which are chemically modified at a single position relative to their canonical counterparts (for example, a chlorine atom instead of a hydrogen atom), exhibit high antiviral activity. However, they are highly toxic for clinical use. This high antiviral activity is due to their ability to mimic natural physiological nucleosides, making them indistinguishable from human nucleic acid polymerases. However, this toxicity of modified nucleosides can be significantly reduced when modifications occur at multiple sites [53]. This is due to the fact that nucleosides substituted at two or more positions on the pyrimidine ring are not recognized as substrates by human DNA polymerases, but viral nucleic acid polymerases readily incorporate them, even with di- or tri-substituted nucleosides, or non-canonical nucleosides [53]. This feature can be explained by a lower selectivity of viral polymerases than human DNA polymerases, which have a much more complex structure. Our M6CU*-modified nucleoside bearing two substituents, a chlorine atom and a methyl group, is thus expected to be relatively less toxic as a potential antiviral agent, since it would not be recognized by human polymerases.

#### 2.7.1. Effect in the Base-Pair Formation with Nucleobases

**Figure 11:**
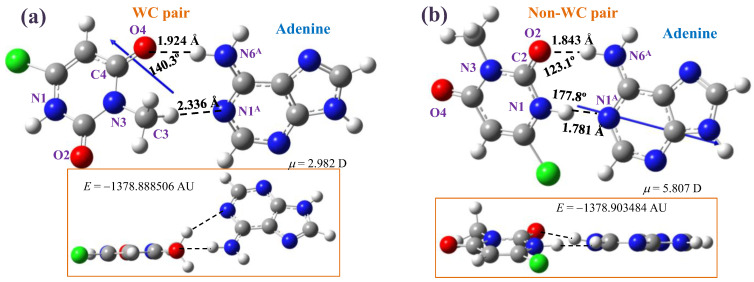
Two views of the effect of the chlorine atom and the methyl group of M6CU calculated at the MP2/6-311++G(3df,pd) level: (**a**) in the WC and (**b**) non-WC pairs with adenine. The blue arrow corresponds to the dipole moment vector.

M6CU has two possible base-pair formations with adenine, Figure 11: (i) the standard WC pair through the intermolecular H-bond C4=O^M6CU*^···H6^adenine^ and, (ii) the non-WC pair through both the C2=O^M6CU*^···H6^adenine^ and N1-H^M6CU*^···N1^adenine^ H-bonds. The WC pair with M6CU is weak because it is mainly formed through the C4=O bond, due to the possibility of another intermolecular H-bond involving the methyl hydrogen of M6CU* C3-H···N1^adenosine^ being too weak to qualify as an H-bond and thus it should be considered as a weak contact. Unlike of the planar WC through uracil, this is also non-planar (Figure 11a). These features indicate that although this WC with M6CU is significantly weaker than the canonical one, it is also possible and stable and could be established if this molecule is inserted into the primer of the cancer-related or viral RNA growth.

However, the non-WC pair with adenine (Figure 11b) is remarkably more stable, with a stability similar to that calculated for the canonical WC pair formed through the N3-H and C4=O groups. It also has a higher dipole moment value and different direction vector than the WC pair. In this new pair, the M6CU (pyrimidine) ring is remarkably more deformed at 5.451 kJ/mol than the adenine ring, at 2.195 kJ/mol, as in the canonical pairs, and the ${∆E}_{A\text{-}C}^{CP}$ value is also in agreement with that determined in the canonical U:A pair, and it is also nearly planar. All of these features indicate that this M6CU molecule could also interfere with RNA growth.

**Figure 12:**
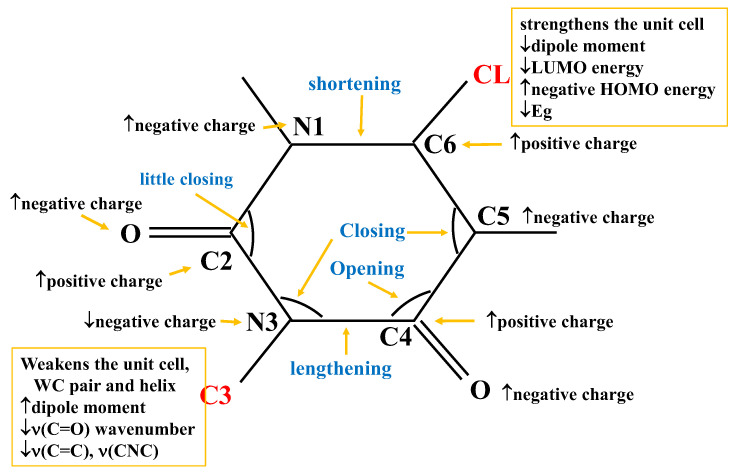
Summary of the chlorine and methyl substitution effects on the uracil ring as compared to the uracil molecule.

A summary of the chlorine and methyl substitution effects on the geometrical parameters, intermolecular H-bonds, NBO charges, and vibrational wavenumbers is shown in schematic form in Figure 12, which facilitates understanding of the manuscript.

#### 2.7.2. Effect in the Optimized RNA:RNA Microhelices

M6CU, as a modified halogenated nucleobase, could be expected to behave similarly to other halogenated uracil derivatives by forming nucleosides upon attachment to the furanose ring and subsequently undergoing phosphorylation by viral nucleic acid polymerases. To elucidate this property, and to theoretically assess the possible potential of M6CU as an anticancer or antiviral drug through its direct effect on cancer/viral RNA helices, a simplified microsystem of the RNA:RNA double-stranded helix (microhelix) was fully optimized at the M06-2X/6–31G(d,p) level, as well as at the M06-2X-D3/6-31G(d,p) level including dispersion correction D3. The main aim was to evaluate the extent of helix deformation induced by M6CU.

Because of the large computing time and memory required, the optimized helix considered has only three nucleotide base pairs, but it was sufficient to assess the geometric effect on the helix of M6CU. This simple model with three nucleotide base pairs is used today for accurate DFT methods by many authors. The double-stranded 5′-UUU-3′ microhelix, with only pyrimidines in one strand and purines in another was selected as it is the simplest one. This short notation used for microhelices, which includes only the nucleosides of one strand, was described in detail in one of our previous works [54]. Among the helix types, the B-type helix with the furanose ring in C_2_′-*endo* orientation [55] was the mainly type optimized as it is the most common in DNA/RNA double helices and the most significantly stable according to our calculations. This helix noticeably differs from A-type (C_3_′-*endo*), which is mainly observed in RNA and hybrid helices [56], and it is also optimized.

**Figure 13:**
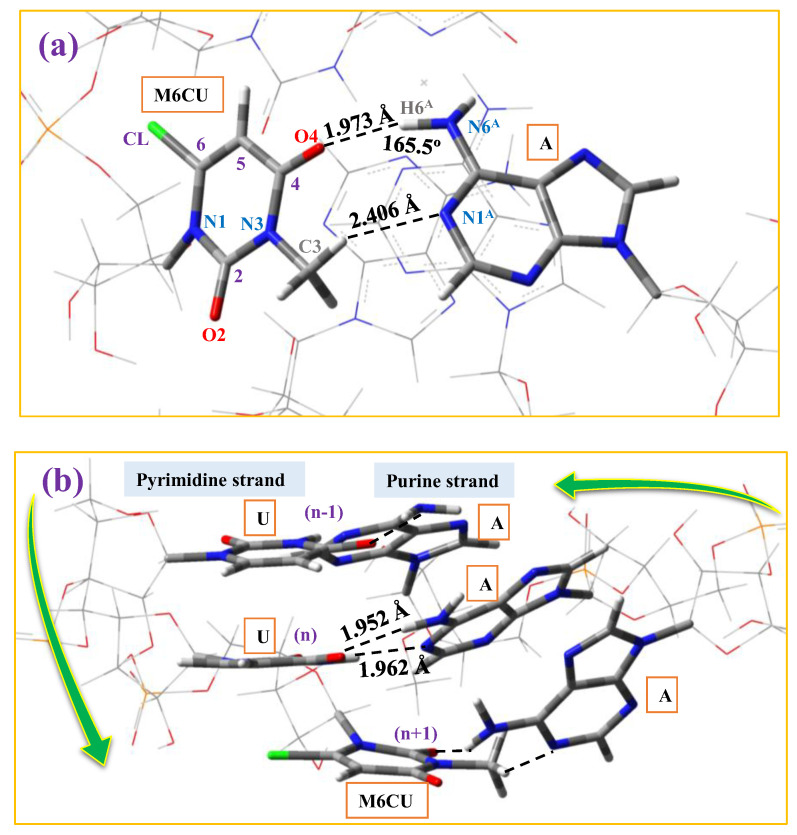
Two views of the M6CU effect inserted into the optimized double-stranded 5′-UUU-3′ microhelix at the M06-2X/6-31G(d,p) level in the B-type. The values of the intermolecular H-bonds in dashed blue lines are included. The oxygen atoms are in red, the nitrogens in blue and the chlorine atom in green. (**a**) View with the M6CU pair in plane n+1. (**b**) View with the helix deformation in the different base pair planes.

The nucleoside (M6CU*) constructed via the N1 atom of the modified M6CU nucleobase was first placed in the *n + 1* plane of the 5′-UUU-3′ microhelix, Figure 13, yielding a microhelix called 5′-U U M6CU*-3′. The effect of M6CU* in the upper *n*-1 and *n* planes could thus be analyzed in comparison with the canonical uridine 5′-UUU-3′ helix. Two views of the optimized microhelix obtained in the B-form are shown in Figure 13, while those in the A-form are plotted in Figure S13. The base pair between uridine with the complementary adenosine nucleoside was established through the two intermolecular H-bonds N3-H^uridine^···N1^adenosine^ and C4=O^uridine^···H6^adenosine^. By contrast, only one H-bond C4=O^M6CU*^···H6^adenosine^ was established with the M6CU* nucleoside, because another interaction involving the methyl hydrogen of M6CU* C3-H···N1^adenosine^ is too weak to qualify as an H-bond and should be considered as a weak contact. In the helix, the C4=O···H6 H-bond is weaker and C3-H···N1 slightly stronger than that determined in the WC pair of Figure 11a.

The methyl group of M6CU* also leads to a remarkable deformation of the base pair though steric effects on adenosine, with a substantial lengthening of the N3···N1^A^ distance and a large out-of-planarity of the base pair, as observed in Figure 13b. This H-bonding is only possible due to the high flexibility of both M6CU* and the helix. However, to induce this deformation and out-of-planarity of the base pair in plane *n* + 1, a small deformation of the base pair of uridine with the complementary adenosine in plane *n* is required as also shown in Figure 13b. This non-planarity of the pair in the helix is slightly lower than that observed in the WC pair of Figure 11a, which can be explained by the stacking forces present in the helix.

The weakening of the H-bond in the plane *n* + 1 by the M6CU* nucleoside also affects those of plane *n*, where N3-H···N1^A^ and C4=O···H6^A^ have values of 1.962 and 1.952 Å, respectively, in the B-type vs. 1.953 and 1.842 Å in the canonical microhelix, respectively. The distance between the phosphorous atoms (P_1_···P_2_) in the pyrimidine strand of 7.335 Å is significantly longer than that calculated in the canonical helix of 7.158 Å, while the distance P_3_···P_4_ in the purine strand of 7.217 Å is shorter than the corresponding value in the canonical helix, 7.315 Å. The helix diameter *d* is remarkably shorter (14.569 Å) than the canonical one of 16.573 Å. The propeller twist angle θp and the helical twist ω in the purine strand differ also significantly from the canonical ones, −19.0° and 32.6°, respectively, as well as all helical parameters.

**Figure 14:**
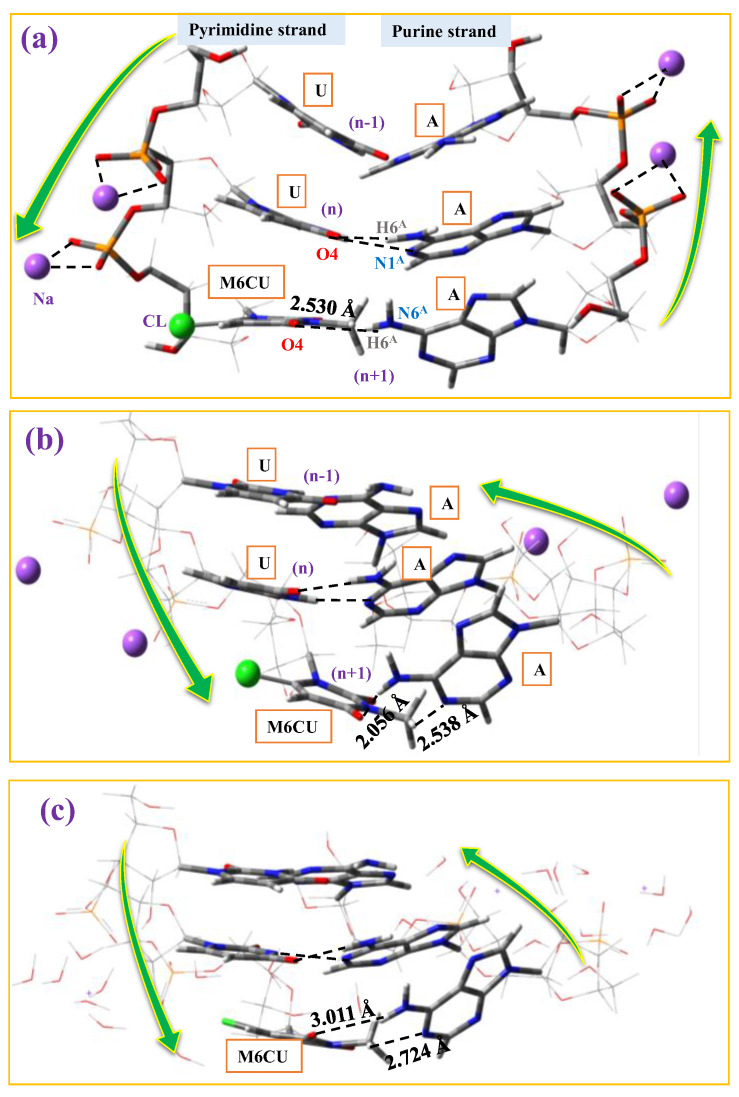
Effect of M6CU when it is inserted into the optimized double-stranded 5′-UUU-3′ microhelix at the M06-2X/6-31G(d,p) level of (**a**) A-type with four sodium cations, (**b**) B-type with four sodium cations, and (**c**) B-type with four sodium cations and twenty water molecules. The values of the intermolecular H-bonds in dashed blue lines are included. The oxygen atoms are in red, the nitrogens in blue and the chlorine atom in green.

The effect with the addition of the sodium cations on the geometric and helical parameters of the A- and B-types was also determined, Figure 14a,b, respectively. These four sodium cations in the microhelix were equidistantly distributed around the phosphate groups. Although these Na cations significantly modify a few geometrical values, as observed in previous publications, the large helix deformation produced by the M6CU* nucleoside remains in both A- and B-types. The effect of the M6CU* nucleoside with the addition of twenty explicit water molecules around the microhelix backbone was also determined for the B-form, Figure 14c. This cluster of water molecules did not significantly modify the helix deformation. According to these results, a noticeable helix deformation is observed when the M6CU* nucleoside is inserted in the plane *n* + 1.

Due to this large effect of the M6CU* nucleoside in the plane *n* + 1, another possibility was to study if this effect is increased or reduced when it was placed in the plane *n* of the helix. In this case, as the calculation progressed the observed effect was that the double helix broke apart and the two strands separated.

Although the methyl group is mainly responsible for deforming and disrupting the RNA helix formation, the effect of the chlorine atom is not negligible. Thus, both its position at the sixth carbon atom of the uracil ring (close to the phosphate moiety) and its small positive charge, which is attracted by the phosphate oxygens, lead to additional helix deformation.

In summary, although our calculations are limited to a type of nucleotide combination on each strand, and due to longer microhelices than three base pairs are currently difficult to perform due to the huge computational time and memory requirements, it is predicted here that when M6CU replaces the uracil nucleobase in the helix, it could hinder/prevent RNA helix formation. Therefore, based on our theoretically optimized microhelix, this M6CU modified nucleobase could exhibit similar behavior to that of other halogenated pyrimidine derivatives [9,10,11,12,13,14,15,16], which possess anticancer/antiviral activity by binding to cancer/viral-derived RNA helices.

### 2.8. Molecular Docking Analysis

Because of the importance of M6CU, we consider it of interest to explore the effect of M6CU in a target protein. Therefore, the optimized structure of M6CU, obtained using the B3LYP DFT method but with cc-pVTZ basis set, was employed for molecular docking calculations [24]. These were performed using AutoDock Tools-1.5.4 interfaced with the MGL Tools-1.5.4 package [57]. By its importance, the target protein of the *β-catenin/Tcf4* complex (PDB ID: 1JPW) was selected for this docking study due to its relevance [58] in the treatment of Multiple Myeloma (MM). MM is an invariably fatal form of cancer characterized by clonal proliferation of malignant plasma cells in the bone marrow [59,60].

The three-dimensional (3D) coordinates of the selected protein file were downloaded from the Research Collaboratory in Structural Bioinformatics (RCSB) protein database [61], with a resolution of 2.5 Å. The protein preparation was carried out through the following three standard steps: (a) first, all water molecules were removed; (b) next, H atoms were inserted to the crystal structure, as well as the Coulomb charges; (c) finally, the previous docked ligand was removed from the protein.

The AutoGrid 4.2 [62,63] program was utilized to generate active site-centered affinity grids with a 120 × 120 × 120 grid size and a spacing of 1.0 Å. In addition, the rigid protein and flexible ligand dockings were carried out with the AutoDock 4.2 program and the Lamarckian genetic algorithm. A total of 100 runs were used to ensure reliable results. To confirm the accurateness of the docking process, a re-docking procedure was also employed [57].

As a result, the best predicted conformation binding energy was −16.3 kJ/mol [24], which appears somewhat low but it could be enough for binding [41]. This is because the methyl group reduces the stretching from the O2 and O4 oxygens, as it was found in the WC base pairs, which were stable. On the other hand, M6CU exhibits an ADA (where A = acceptor, D = donor) H-bond site, with the N1-H group acting as the donor and the neighbor O2 and Cl atoms as (strong and weak, respectively) acceptors. This feature facilitates the binding of M6CU to the amino acids of the protein chain. Thus, in the active site of the 1JPW target protein, the four amino acids were those that appear bonded by the O–H···O or N-H···O hydrogen bonds with the ligand. Halogen bonds (or Cl···O contacts [64]) with amino acids residues were not observed.

**Figure 15:**
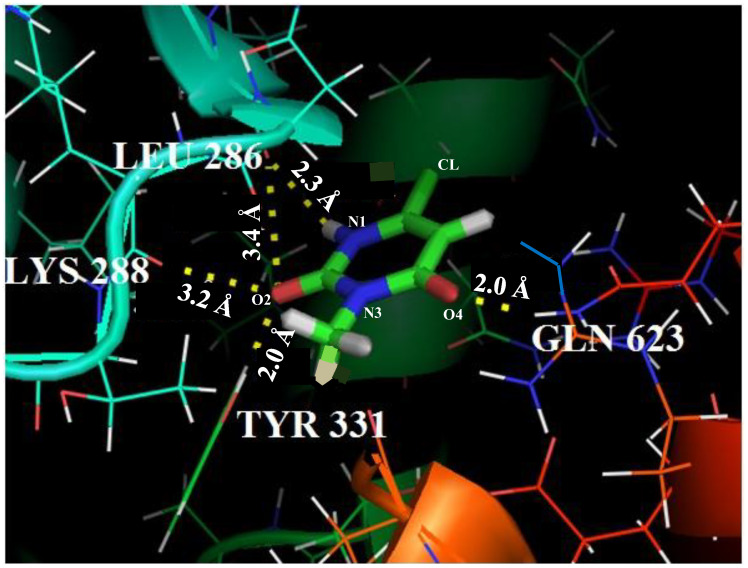
Protein–ligand hydrogen bonding binding projection by molecular docking of M6CU with 1JPW target protein. (i) GLN-623:1HE2, with a bond distance of 2.0 Å to O4 of M6CU (O4···H-N). (ii) TYR-331:OH, with a bond distance of 2.0 Å to O2 of M6CU (O2···H-N). (iii) LYS-288:C, with a bond distance of 3.2 Å to O2 of M6CU (O2···H-O). (iv) LEU-286, with a bond distance of 3.4 Å to O2 of M6CU (O2···H-O). (v) LEU-286:CA, with a bond distance of 2.3 Å to N1 of M6CU (N1-H···O). These results suggest that our M6CU molecule could serve as an active ligand capable of binding to the active site 1JPW of the *β-catenin/Tcf4* complex protein.

The amino acids bonded to M6CU were tyrosine (TYR-331), glutamine (GLN-623), leucine (LEU-286) and lysine (LYS-288), and five hydrogen bonds were formed. The final protein–ligand interaction complex is plotted in Figure 15, and the bonded residues involved as determined by the molecular docking calculations were the following.

### 2.9. Radical-Scavenging Assays

**Figure 16:**
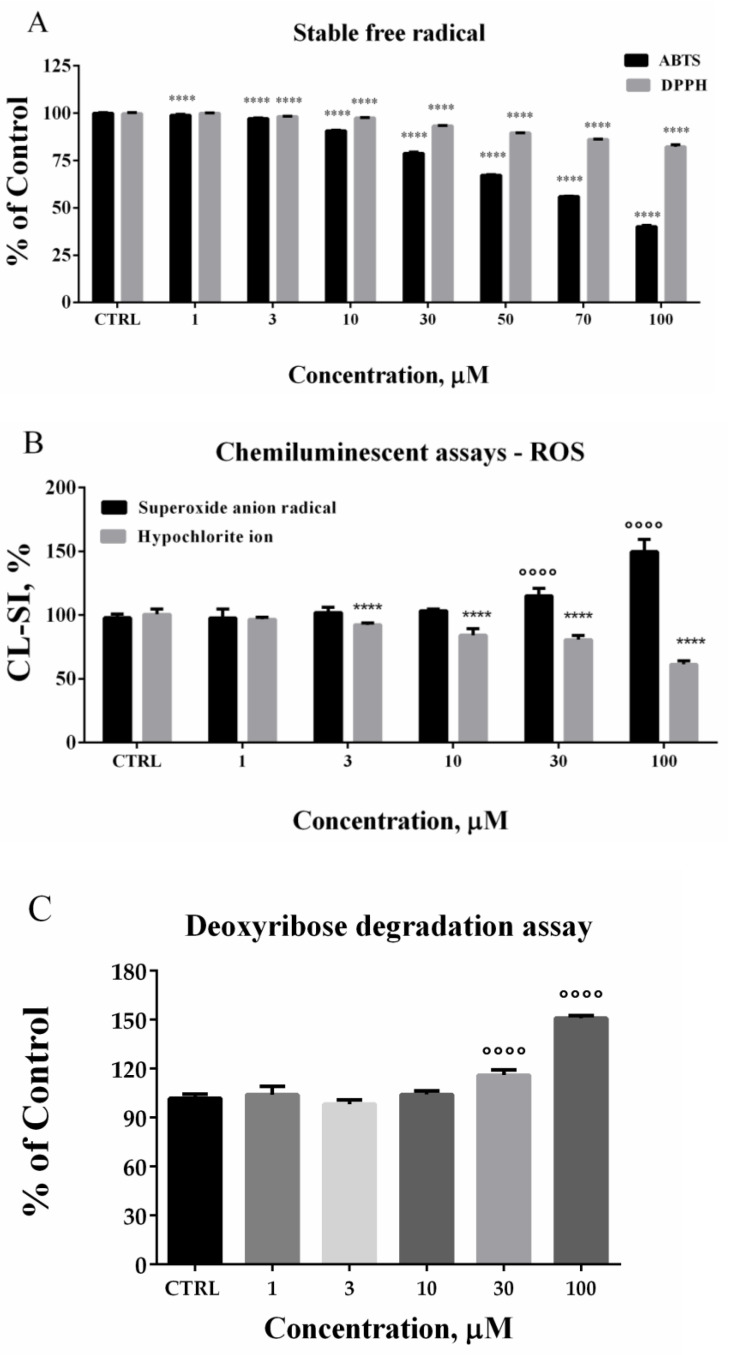
Radical-scavenging activity and anti/prooxidant effect of M6CU: (**A**) RSA in spectrophotometric model systems containing the stable free radicals ABTS and DPPH. (**B**) Scavenging activity in chemiluminescent model systems containing superoxide anion radical and hypochlorite ions. (**C**) Deoxyribose oxidative degradation. Data are shown as the mean ± SD from three independent measurements. Differences were evaluated by one-way analysis of variance (ANOVA) followed by Dunnett’s post hoc test. Significant changes, respectively, such as increases and decreases vs. control, were indicated by ° and * with *p* values as follows: ****/°°°° *p* < 0.0001.

We also consider it of interest to explore the possible antioxidant activity of this M6CU compound. Therefore, the initial assessment of the radical-scavenging properties and antioxidant/prooxidant effects began with evaluating the potency of the compound to decrease the concentrations of stable free radicals—ABTS and DPPH. The M6CU molecule demonstrated the capacity to decrease the concentration of both, although the magnitude of the observed effect differed between the two assays, Figure 16A. In both systems, the effect was concentration-dependent, and the radical-scavenging activity increased progressively with the applied compound concentration. In the system containing the ABTS radical, a minimal but significant effect was already evident even at the lowest tested concentration of 1 µM. The concentration-effect dependence showed a strongly pronounced linear relationship (R^2^ = 0.9943). At the maximal tested concentration, the percentage of control values reached 40%, corresponding to 60% radical-scavenging activity. Using the analytical equation of the concentration-effect relationship, the concentration required to decrease the determined parameter by 50% IC50 was estimated at 80.42 µM. At M6CU concentrations equivalent to those used in the other radical-containing model systems, samples containing DPPH exhibited a lower percentage of reduction than the corresponding control. At the highest tested concentration, only a decrease of less than 20% compared to the control samples was observed.

The effect of M6CU on the chemiluminescent model systems was examined over a concentration range up to 100 µM, Figure 16B. The compound exerted a distinct modulatory effect on the chemiluminescent signal, depending on the ROS (reactive oxygen species) involved, stimulation of the chemiluminescent response in the presence of the superoxide anion radical and a decrease in hypochlorite ion samples. In the presence of O_2_^●−^, no statistically significant effect on the chemiluminescent response was detected at the lower concentration (up to and including 10 µM). Upon reaching 30 µM, a slight but statistically significant increase in the chemiluminescent response was observed, corresponding to an approximately 15% higher CL-index than the controls. At the maximal tested concentration (100 µM), the chemiluminescent response was significantly elevated, exceeding the control values by approximately 50%. This pronounced increase in signal intensity suggests a potential for propagating the oxidative damage to third molecules under these experimental conditions. In the hypochlorite-containing system, no statistically significant effect on the chemiluminescent response was observed at concentrations below or equal to 1 µM. Further, an increase in the concentration led to concentration-dependent suppression of both the chemiluminescent response and the corresponding CL-index. At the lowest concentration (3 µM), where a statistically significant decrease was achieved, the CL-index decreased to 92%. Maximal reduction in the parameter and scavenging activity was observed at the highest tested concentration (100 µM)—the parameter decreased to 61.48%.

In the system of ferrous iron-induced oxidative damage of deoxyribose at either of the tested concentrations, the measured absorbance values were lower compared to the control samples, Figure 16C. This indicates a lack of antioxidant effect under the experimental conditions. At the lower tested concentrations, including 10 µM, no statistically significant effect was observed, and the parameter calculated in the system was indistinguishable from the control. At 30 µM, a slight increase in the oxidative damage to deoxyribose was noted. The observed effect was modest—around a 16% increase compared to the control—but statistically significant. At the highest tested concentration, deoxyribose oxidative degradation increased to approximately 50% above the control values, indicating a strong prooxidant effect.

The mechanism proposed by several authors of deoxyribose oxidative degradation in this system involves autooxidation of Fe(II) by molecular oxygen with subsequent superoxide anion radical formation {1} in combination with direct degradation of the deoxyribose molecule as a result of its interaction with the generated Fe (III) and the other products (4), (5a) and (5b) [65,66].

Fe(II) + O_2_ ↔ Fe(III) + O_2_^●−^ (1)

2O_2_^●−^ + 2H^+^ → H_2_O_2_ + O_2_ (2)

Fe(II) + H_2_O_2_ → Fe(III) + OH^−^ + ^●^OH (3)

^●^OH + 2-Deoxyribose → 2-Dr^●^ Decomposition → MDA (4)

Fe(III) + 2-Deoxyribose → Fe(III) − 2-Dr → Fe(II) + 2-Dr^●^ (5a)

2-Dr^●^ → 2-Dr^●^ Decomposition → MDA (5b)

The importance of the superoxide anion radical in the proposed mechanism for the oxidative damage of deoxyribose and the observed increase in the chemiluminescent signal in the system containing this ROS could provide an explanation for the observed prooxidant effect at concentrations of 30 µM and 100 µM.

## 3. Computational Methods

The calculations were performed using the second-order Møller–Plesset MP2 [67] ab initio method and the Density Functional Theory (DFT) method [68]. These quantum chemical methods are included in the GAUSSIAN-16 program package (C. 01) [69] at the Computing Center of the Universidad Complutense in Madrid. This package was run under the UNIX system with standard parameters. DFT methods were mainly used because they are considered the most appropriate methods currently available today because they represent a balance between the desired chemical accuracy in the results and the large computation time and computing power required to obtain them. In addition, these methods yielded accurate results in spectroscopic studies [70,71] and in drug design [72,73].

Among the DFT methods, the B3LYP with the Becke’s three-parameter exchange functional (B3) [74] in combination with the non-local correlation functionals of Lee, Yang and Parr (LYP) was mainly used [75]. This is because it is among the most widely used DFT methods that provides excellent calculated wavenumbers for the IR and Raman spectra [47]. The CAM-B3LYP method was also utilized [76] because it provides accurate wavenumbers for noncovalent weak interactions, such as those that occur in trimer form. The dispersion correction (D3) [77,78] was also used with the B3LYP method in the trimer form. Another DFT method, the Minnesota functional M06-2X [79,80,81], was also selected because it is considered one of the best meta-generalized gradient functionals for analyzing dispersion-bound systems [82,83], and it is especially appropriate to reproduce the RNA:RNA and DNA:DNA double-stranded microhelices [54]. The dispersion correction (D3) was also used with this M06-2X method.

Several basis sets were employed for geometry optimization and wavenumber computation, ranging from 6-31G(d,p) to 6-311++G(3df,pd). The optimized geometry of M6CU and all molecules under study was determined by minimizing energy with regard to all structural parameters, and without imposing molecular symmetry constraints. For this optimization process, the Berny algorithm (included in the GAUSSIAN-16 software package) was considered the most appropriate under the “TIGHT” convergence criteria. The calculated harmonic wavenumbers always correspond to those obtained at the same level of theory in the previous optimization process, and they were analyzed to confirm the stationary points of all optimized structures, in addition to their spectroscopic values. Only positive harmonic wavenumbers (true energy minimum) were finally obtained. The calculated Natural Bond Orbital (NBO) atomic charges [84,85] represent one of the most useful tools to relate properties.

Raman scattering activities (*S*_i_) were calculated using Gaussian-16 and they were converted into relative Raman intensities (*I*_i_) utilizing the well-known relationship derived from basic Raman scattering theory [86].

$$I_{i}=\frac{f{\left(\upsilon_{0}-\upsilon_{i}\right)}^{4}S_{i}}{\upsilon_{i}[1-\exp(-hc\upsilon_{i}/kT)]}$$

where *υ*_o_ corresponds to the excitation wavenumber (cm^−1^) and *υ*_i_ to the theoretical vibrational wavenumber of the *i*th normal mode. The constants *h*, *c* and *k* correspond to the Planck constant, light speed and Boltzmann constant, respectively. *T* indicates the Kelvin temperature (298.15 K). The parameter *f* corresponds to a common scaling factor that has been chosen to be suitable for all band intensities, and *S_i_* corresponds to the theoretical Raman scattering activity of normal mode *i* (Å^4^ amu^−1^).

### Calculation of the Interaction Energies (E^int^) in the Trimer Form of M6CU

E^int^ between the M6CU molecules in the two trimer forms of M6CU was calculated using both the B3LYP/6-311++G(3df,pd) and CAM-B3LYP/6-311++G(3df,pd) levels. These represent the possible theoretical forms for the solid state. For comparison purposes, the values for the 3MU and M5CU molecules were also determined. Thus, the effect of the chlorine atom on the stability of the crystal through a trimer form was determined.

All of these E^int^ energies were corrected for basis-set superposition error (BSSE) according to the Boys & Bernardi procedure [87,88]. Therefore, the total corrected interaction energy by the counterpoise (CP) procedure, ${∆E}_{A\text{-}C}^{CP}$, in the trimer (A-C) was calculated as follows:

(6) $${∆E}_{A\text{-}C}^{CP}=E^{int}(A\text{-}C)+E^{def}(A\text{-}C)$$

where (A-C) correspond to the three molecules (monomers) of the trimer, labeled as A, B, and C, and where E^int^(A-C) between them can be calculated as follows:

(7) $$E^{int}(A\text{-}C)\;=\;E_{A\text{-}C}^{A\text{-}C}(A\text{-}C)-E_{A}^{A\text{-}C}(A\text{-}C)-E_{B}^{A\text{-}C}(A\text{-}C)-E_{c}^{A\text{-}C}(A\text{-}C)$$

$E_{A\text{-}C}^{A\text{-}C}(A\text{-}C)$ corresponds to the electronic energy of the whole trimer, and $E_{A}^{A\text{-}C}$(A-C) to the electronic energy of monomer (A) in this trimer, and so on.

The total deformation energy E^def^(A-C) can be calculated as the sum of the deformation energies of each molecule in the trimer as follows:

(8) $$E^{def}\;(A\text{-}C)\;=\;E_{A}^{def}(A\text{-}C)+E_{B}^{def}(A\text{-}C)+E_{C}^{def}(A\text{-}C)$$

where $E_{A}^{def}$ represents the deformation energy of monomer (A) within the trimer (A-C), and so on, and this can be calculated in molecule (A) as follows:

(9) $$E_{A}^{def}(A\text{-}C)=E_{A}^{A}(A\text{-}C)-E_{A}^{A}(A)$$

The parentheses specify whether the calculations have been carried out at the optimized geometry of monomer (A) or trimer (A-C). Superscripts indicate that the computations have been done with the basis set of monomer (A), and the subscript points out monomer (A).

In the WC base pair (A-C), E^def^(A-C) corresponds to the following equation:

(10) $$E^{def}(A\text{-}C)\;=\;E_{A}^{def}(A\text{-}C)+E_{C}^{def}(A\text{-}C)$$

where $E_{A}^{def}$ or $E_{C}^{def}$ indicate each molecule of the pair A ≡ M6CU and C ≡ adenine, which were similarly determined in ec. (9).

## 4. Experimental Section

### 4.1. Experimental Details

The spectrally pure sample of M6CU (m.p. > 300 °C, solid) was obtained from Sigma Chemical Co, St. Louis, MO, USA and used as such without any further purification. The FT-Raman spectrum of solid M6CU was recorded with a Dilor Labram spectrometer (Horiba-Jobin-Yvon, model LabRam, Paris, France) using the 784.8 nm excitation line from a near-infrared diode laser. The Labram integrated system is coupled through an Olympus LMPlanFL (Tokyo, Japan) 50× objective to the optical microscope. The spectra were collected in the backscattering geometry with a resolution of 1 cm^−1^. The detection of Raman signal was carried out with a Peltier-cooled CCD camera (Münster, Germany). A laser power of 35 mW was used in our measurements.

The solid-state FT-IR spectrum was recorded in the 600–3600 cm^−1^ range using a KBr pellet via FT-IR 113V Bruker spectrometer (Ettlingen, Germany). For spectrum acquisition, 100 scans were collected at 1 cm^−1^ resolution.

The radical-scavenging activity (RSA) and antioxidant potential of M6CU were evaluated using spectrophotometric and chemiluminescent chemical model systems. RSA has been measured against stable free radicals as well as reactive oxygen species (ROS). Assessment using the 2,2′-azino-bis (3-ethylbenzothiazoline-6-sulfonic) acid radical cation (ABTS^●+^) and the 2,2-diphenyl-1-picrylhydrazyl radical (DPPH^●^) will provide insight into the general reactivity of the compound in systems, suggesting different molecular mechanisms of radical elimination, i.e., hydrogen-atom transfer or single-electron transfer. Being accessible, rapid and operationally simple, both methods are widely used for early-stage screening of radical-scavenging potential [89]. The second group of data, using a chemiluminescent approach with biologically relevant ROS, will enable evaluation of the compounds’ potency to reduce oxidative stress in its initial phase. Chemiluminescent methods are extensively utilized in analytical investigations because of their extremely low detection limits (picomolar concentrations) [90,91]. Additionally, the potency of the compound to reduce deoxyribose oxidative degradation under conditions of Fe(II)-induced oxidative damage has been determined. Taken together, the data obtained from these complementary approaches will provide a comprehensive characterization of the antioxidant and radical-scavenging profile of M6CU and its potential biological relevance.

### 4.2. Materials

As a first step to evaluate the potential interference with the spectrophotometric assays, the absorbance spectrum of M6CU was recorded. Negligible intrinsic absorbance compared to the signal of the untreated control was detected at the wavelength used to determine the effect using the DPPH test (λ = 517 nm—0.0003), TBA-RS methods (λ = 532 nm—0.0002) and ABTS assay (λ = 734 nm—0.0014). The presented data indicate an absence of spectral overlap with the analytical wavelength. For all spectrophotometric systems used to evaluate scavenging activity, two groups of reaction mixtures have been prepared: samples containing the radical and M6CU (at different concentrations), and controls where the tested substance has been omitted. For both chemiluminescent assays, each analytical run included a blank sample, a control solution (without the investigated compound), and sample solutions containing different concentrations of M6CU. In accordance with the requirements of the MultiUse software (version 5.4), each analytical run began with the measurement of a blank sample in the absence of the enhancer. The signal obtained from this initial blank measurement was automatically used by the software for background correction by subtracting it from the signal of the subsequent control and sample measurements acquired within the same analytical run. The measurements of ABTS, DPPH radicals and deoxyribose degradation were performed using a Genesys 50 spectrophotometer (Thermo Scientific, Madison, WI, USA). Chemiluminescent experiments were performed using an LKB 1251 chemiluminometer (Bioorbit, Turku, Finland) connected to an IBM-PC computer. Data were analyzed using MultiUse software, version 1.08 (Bioorbit). The effects of the tested compound were investigated in all assay systems over concentration ranges extending up to 100 µM.

### 4.3. Methods

#### 4.3.1. Spectrophotometric Assays

ABTS test [92]: A stock solution of the stable ABTS radical cation was prepared by mixing 14 mM ABTS with potassium persulfate (final concentration: 2.45 mM). The obtained mixture was allowed to stay for 16 h to ensure complete oxidation of ABTS and stable absorbance. This solution was diluted with phosphate-buffered saline (PBS) to obtain a control sample with an absorbance of 0.70 ± 0.01. All samples and controls were incubated for 60 min at room temperature, and the final absorbance was measured at 734 nm.

DPPH test [93]: A purple DPPH radical working solution with an absorbance of 1 ± 0.01 was prepared using ethanol as a solvent. After mixing the DPPH solution with various concentrations of M6CU, the reaction mixtures were incubated for 1 h at room temperature in the dark. Absorbance was determined at 517 nm.

Iron-induced deoxyribose degradation test [66]: The thiobarbituric acid reactive-substance test was used. Deoxyribose (0.5 mM) oxidative damage was induced by adding FeCl_2_ (0.1 mM). All measurement compositions were incubated at 37 °C for 30 min. After this incubation, 0.5 mL of 2.8% trichloroacetic acid and 0.5 mL of 0.5% thiobarbituric acid were added. A second incubation at 100 °C for 20 min was performed. Absorbance was measured at 532 nm.

#### 4.3.2. Chemiluminescent Assays

Luminol-dependent chemiluminescence in a system of KO_2_-generated superoxide anion radical (O_2_^●−^): We prepared 1 mL samples of 50 mM phosphate buffer, pH 7.4, containing 0.1 mM luminol and the tested compound at different concentrations. In the control samples, M6CU was omitted. The CL response was measured immediately after adding 20 μL of KO_2_ solution (dissolved in DMSO). The chemiluminescence was recorded for 1 min at 50 ms intervals.

Luminol-dependent CL in a system of NaOCl-generated hypochlorite: We prepared 1 mL samples of PB, pH 7.4, containing 0.1 mM luminol, 0.06 mM NaOCl and M6CU at varying concentrations or buffer (for control). The chemiluminescence was recorded for 1 min every 50 ms after the addition of NaOCl.

The CL ratio in the presence/absence of the tested compound, expressed as a percentage, was defined as the chemiluminescent scavenging index (CL-SI). It was used to estimate and compare the scavenging properties.

Statistical analysis: Data were analyzed with GraphPad Prism 8 (v8, GraphPad Software, La Jolla, CA, USA). Values obtained from three independent measurements have been presented as the mean ± SD. Differences were evaluated by one-way analysis of variance (ANOVA) followed by Dunnett’s post hoc test. Statistical significance was set at *p* < 0.05 (* *p* < 0.05, ** *p* < 0.01, *** *p* < 0.001, **** *p* < 0.0001).

## 5. Summary and Conclusions

The role of the chlorine atom and methyl group of M6CU in its structure, NBO charge distribution, vibrational spectra, molecular properties and noncovalent interactions were analyzed for the first time in this work using both quantum chemical calculations and experimental vibrational spectra. The key findings of this study were as follows:

(i) Methyl substitution increases the dipole moment and slightly alters its direction. Chlorine substitution increases the negative charge of both nitrogens and slightly decreases the negative charge on both oxygens in M6CU, whereas methylation increases the negative charge on these oxygens. Several correlations were identified by comparing M6CU with M5CU, 3MU, 5-CU, 6-CU, and uracil molecules.
(ii) A structural characterization and a detailed analysis of the IR and Raman spectra of M6CU were carried out in the gas phase and in the solid state (using a trimer model) using the MP2 and several DFT quantum chemical methods. An accurate characterization and assignment of the experimental bands were performed using three DFT methods and two scaling equation procedures to correct the computed wavenumbers.
(iii) The chlorine atom and methyl group of M6CU lead to different possible crystal unit-cell arrangements for the solid-state sample than 3MU and M5CU. In M6CU, two possible arrangements using a trimer form were optimized, and their interaction energies were calculated. The B-form appears to be the most stable; however, the IR and Raman spectra of the A-form showed the best agreement with the experimental IR and Raman spectra. All values reported in this manuscript are the most accurate to date.
(iv) M6CU exhibits higher intramolecular charge transfer and reactivity than 3MU, 6-CU and uracil molecules due to its smaller HOMO–LUMO energy gap. M6CU also has Ea, χ, η, and S values that are remarkably lower than the other molecules studied, consistent with the behavior of a soft molecule.
(v) In the monomer form, the methyl group in the third position causes a red-shift in the ν(C2=O) stretching wavenumber of ca. 15 cm^−1^ and in the ν(C4=O) mode by ca. 35 cm^−1^, with an increase in their coupling of up to 20% PED between both ν(C=O) modes. The chlorine atom has little effect on this coupling and the shift of the ν(C=O) wavenumbers.
(vi) The methyl group induced a significant red shift of ~40 cm^−1^ in the strong experimental IR band corresponding to the ν(C=C) stretching, mode 24, and of ~25 cm^−1^ in the band corresponding to the ν(CNC) stretching, mode 21.
(vii) The distortions observed in the helical parameters, backbone stability, intermolecular H-bonds between the two strands, and in the base-stacking interactions were determined upon insertion of M6CU into a B-type RNA:RNA double microhelix with a 5′-UUU-3′ sequence.
(viii) M6CU forms stable WC base pairs with adenine, showing similar behavior to that of other uracil derivatives, although slightly weaker, and it can also form a stable WC pair with adenosine in a microhelix. Therefore, a M6CU-modified nucleobase could exhibit similar behavior to other uracil derivatives and replace uracil in the helix and, based on our calculations, lead to a noticeable helix deformation.
(ix) Molecular docking calculations revealed that M6CU could act as a ligand capable of binding to several amino acids within the active site (1JPW) of the target *β-catenin/Tcf4* protein complex.
(x) The antioxidant activity of this compound reveals its ability to reduce the absorbance in samples containing the stable chemical radicals (ABTS and DPPH) and the CL-signal in the presence of the hypochlorite ions. At concentrations below 30 µM, M6CU does not cause oxidative damage to the biologically relevant component of DNA, i.e., deoxyribose. The observed increase in oxidative degradation above this concentration threshold is in accordance with data from the chemiluminescent system containing the superoxide anion radical and the molecular mechanism underlying the oxidative injury.

These theoretical results characterize the uracil derivative M6CU and, as a first approximation, show the possible potential of this compound for other applications. Moreover, our group is interested to compare these data with the antioxidant activity of new derivatives and metal complexes of this compound.

## Figures and Tables

**Table 1:** Geometrical parameters, bond lengths (in A) and angles (in degrees) of M6CU at different theoretical methods with the 6-311++G(3df,pd) basis set.

Parameters	M6CU								Uracil	6-CU	3MU	M5CU	Uracil	3MU
	Monomer		Trimer A-Form			Trimer B-Form			Monomer				Experimental	
	B3LYP	MP2	B3LYP	CAM- B3LYP	B3LYP-D3	B3LYP	CAM- B3LYP	MP2	MP2				ED ^a^	X-Ray ^b^
Bond lengths														
N1-C2	1.394	1.389	1.382	1.373	1.380	1.374	1.365	1.368	1.384	1.389	1.383	1.380	1.395 (6)	1.359
C2-N3	1.381	1.379	1.386	1.380	1.386	1.378	1.372	1.375	1.379	1.376	1.382	1.385	1.391 (6)	1.398
N3-C4	1.418	1.411	1.405	1.394	1.404	1.410	1.399	1.402	1.402	1.402	1.411	1.407	1.415 (6)	1.404
C4-C5	1.454	1.451	1.440	1.438	1.439	1.440	1.438	1.437	1.453	1.452	1.451	1.463	1.462 (8)	1.419
C5=C6	1.341	1.347	1.346	1.338	1.346	1.346	1.338	1.352	1.348	1.348	1.346	1.348	1.343 (24)	1.320
N1-C6	1.364	1.363	1.361	1.358	1.360	1.363	1.360	1.361	1.370	1.368	1.366	1.366	1.396	1.355
N1-H	1.009	1.012	1.031	1.031	1.033	1.034	1.034	1.040	1.010	1.011	1.010	1.010		
C2=O	1.211	1.216	1.217	1.213	1.218	1.227	1.222	1.232	1.214	1.212	1.217	1.217	1.212 (3)	1.235
N3-C3	1.465	1.460	1.465	1.458	1.465	1.466	1.459	1.461	-	-	1.459	1.461		1.462
C4=O	1.214	1.219	1.229	1.224	1.230	1.227	1.222	1.232	1.218	1.217	1.220	1.216	1.211 (3)	1.245
C5-H	1.077	1.079	1.080	1.080	1.080	1.080	1.080	1.084	1.079	1.079	1.079	-		
C6-CL	1.724	1.708	1.722	1.712	1.722	1.719	1.709	1.704	-	1.708	-	-	-	
Bond angles														
N-C2-N	115.0	114.7	115.7	115.9	115.8	116.4	116.6	116.3	112.8	112.9	114.6	114.4	114.6 (20)	114.2
C-N3-C4	124.8	125.5	124.2	124.1	124.1	123.8	123.7	124.4	128.4	128.2	125.7	126.3	126.0 (14)	125.4
N-C4-C	115.4	115.1	116.2	116.4	116.3	116.2	116.3	116.0	113.5	114.0	114.6	113.9	115.5 (18)	116.0
C4-C5=C	119.7	119.6	119.0	118.7	118.9	119.0	118.8	118.8	119.7	119.1	120.3	120.6	119.7 (21)	117.0
N1-C6=C5	121.7	121.7	122.5	122.6	122.5	122.3	122.4	122.3	121.9	122.5	121.0	120.6		126.3
C2-N1-C6	123.4	123.3	122.4	122.3	122.4	122.3	122.1	122.2	123.7	123.3	123.8	124.1		121.0
C2-N1-H	115.6	115.7	116.2	116.3	116.5	115.9	116.0	116.4	115.1	116.1	114.7	114.9	115.7	
N1-C2=O	121.0	121.6	121.9	122.0	122.0	121.7	121.8	122.3	123.1	122.5	122.1	122.4		124.6
C2-N3-C3	116.2	115.6	116.2	116.2	116.0	116.6	116.6	116.1	-	-	115.4	115.3		115.6
N3-C4=O	120.9	121.0	119.8	119.8	119.8	119.6	119.6	119.6	120.4	120.5	121.0	121.8	120.2	120.6
C5=C6-CL	123.3	123.3	122.3	122.4	122.3	122.6	122.7	122.8	-	123.0	123.0 ^a^	-		
C6-N1-H	121.1	120.9	121.4	121.4	121.1	121.8	121.9	121.4	121.2	120.6	121.5	120.9		

^a^ Ref. [40]. ^b^ Ref. [41].

**Table 2:** NBO total atomic charges on the atoms of M6CU with different DFT methods and with the 6-311++G(3df,pd) basis set.

Atom	M6CU							Uracil	6-CU	3MU
	Monomer		Trimer A-Form		Trimer B-Form			Monomer		
	B3LYP	MP2	B3LYP	CAM- B3LYP	B3LYP	CAM- B3LYP	MP2	MP2		
N1	−0.624	−0.714	−0.642	−0.658	−0.639	−0.654	−0.743	−0.704	−0.719	−0.698
C2	0.836	1.025	0.842	0.861	0.851	0.871	1.043	1.010	1.015	1.020
N3	−0.510	−0.625	−0.497	−0.504	−0.490	−0.497	−0.605	−0.737	−0.736	−0.626
C4	0.670	0.836	0.682	0.706	0.682	0.706	0.858	0.819	0.824	0.830
C5	−0.375	−0.421	−0.398	−0.414	−0.389	−0.405	−0.457	−0.412	−0.424	−0.408
C6	0.210	0.301	0.228	0.239	0.226	0.237	0.326	0.184	0.300	0.184
H1	0.426	0.430	0.467	0.471	0.468	0.473	0.492	0.420	0.431	0.418
O2	−0.627	−0.729	−0.656	−0.669	−0.684	−0.696	−0.793	−0.732	−0.721	−0.740
C3	−0.359	−0.261	−0.359	−0.368	−0.359	−0.369	−0.262	-	-	−0.261
O4	−0.604	−0.705	−0.670	−0.681	−0.661	−0.672	−0.772	−0.700	−0.694	−0.712
H5	0.248	0.238	0.285	0.290	0.284	0.289	0.286	0.228	0.240	0.226
CL	0.063	0.054	0.075	0.076	0.085	0.084	0.073	0.198 ^a^	0.057	-

^a^ With hydrogen atom.

**Table 3:** Theoretical computed total energies (A.U.), Gibbs energies (A.U.), rotational constants (GHz), entropies (J mol^−1^ K^−1^), dipole moments (Debyes), and constant volume heat capacity (C_v_, J mol^−1^ K^−1^) in the monomer form of M6CU, uracil, 6-CU and 3MU molecules with different methods and with the 6-311++G(3df,pd) basis set.

Parameters	M6CU		Uracil	6-CU	3MU
	B3LYP	MP2	MP2		
Total energy + ZPE	−913.81614	−912.422092	−414.096764	−873.128640	−453.31243
Gibbs energy	−913.85129			−873.161428	
Rotational constants: A	1.999	1.998	3.906	2.029	2.388
B	0.856	0.864	2.023	1.149	1.996
C	0.602	0.605	1.333	0.734	1.095
Entropy: Total	398.02	398.99 ^a^	333.00 ^a^	362.27	369.66 ^a^
Translational	172.05	172.05 ^a^	167.61 ^a^	170.74	169.08 ^a^
Rotational	126.02	126.11 ^a^	116.48 ^a^	123.82	119.41 ^a^
Vibrational	99.91	100.83 ^a^	48.91 ^a^	67.72	81.17 ^a^
Dipole moment	2.506	3.088	5.024	3.781	4.386
Heat capacity, C_v_	136.73	137.19 ^a^	98.41 ^a^	114.11	369.66 ^a^

^a^ With the 6-31G(d,p) basis set.

**Table 4:** The interaction energies E^int^(A–B), deformation energies E^def^, and total CP corrected interaction energy, ${∆E}_{A\text{-}C}^{CP}$ in kJ/mol, were mainly calculated in the trimer forms of M6CU, 3MU and M5CU, using the B3LYP and CAM-B3LYP DFT methods and the MP2 ab initio method. In all trimers by DFT methods the 6-311++G(3df,pd) basis set was utilized, where “A” corresponds to monomer with ring *I*, “B” to ring *II*, “C” to ring *III*, and A-C to these three monomers of the trimer. In the non-WC pair M6CU:adenine, “A” corresponds to M6CU, “B” to adenine, and A-C to the whole pair.

System	Figure	Method	$E_{A}^{def}$(A-C)	$E_{B}^{def}$(A-C)	$E_{c}^{def}$(A-C)	E^def^(A-C)	E^int^(A-C)	${∆E}_{A\text{-}C}^{CP}$
M6CU	Figure 4a (A-form)	B3LYP	1.972	3.531	1.239	6.742	−88.695	−81.952
		CAM-B3LYP	2.106	3.810	1.350	7.265	−100.793	−93.528
		B3LYP-D3	2.245	4.038	1.426	7.708	−119.476	−111.768
	Figure 4b (B-form)	B3LYP	3.319	4.899	1.696	9.914	−111.418	−101.504
		CAM-B3LYP	3.563	5.290	1.814	10.667	−125.528	−114.860
		MP2	3.972	5.776	1.869	11.618	−147.684	−136.067
3MU	Figure 4c	B3LYP	3.298	4.855	1.688	9.840	−121.274	−111.434
		CAM-B3LYP	3.502	5.083	1.806	10.392	−134.118	−123.727
		MP2	3.710	5.099	1.833	10.641	−147.775	−137.134
M5CU ^a^	Figure 4d	B3LYP	3.707	3.707	-	7.414	−76.843	−69.429
M6CU:Adenine	11	MP2	5.451	2.195	-	7.645	−82.835	−75.189

^a^ In dimer form.

**Table 5:** Theoretically computed total energies (A.U.), Gibbs energies (A.U.), rotational constants (GHz), entropies (cal mol^−1^ K^−1^), constant-volume heat capacity (C_v_, J mol^−1^ K^−1^) and dipole moments (Debyes) for the trimer form of M6CU calculated using different methods and the 6-311++G(3df,pd) basis set.

**Parameters**	**Trimer A-Form**		**Trimer B-Form**		
	**B3LYP**	**CAM-B3LYP**	**B3LYP**	**CAM-B3LYP**	**MP2**
Total energy + ZPE	−2741.477035	−2740.890161	−2741.484168	−2740.898003	−2737.318101
Gibbs energy	−2741.549019	−2740.961360	−2741.554345	−2740.967558	
Rotational constants: A	0.289	0.292	0.289	0.292	0.292
B	0.038	0.038	0.040	0.040	0.041
C	0.033	0.034	0.035	0.035	0.036
Entropy: Total	915.96	905.42	896.55	887.55	
Translational	185.73	185.73	185.73	185.73	
Rotational	159.08	158.91	158.66	158.49	
Vibrational	571.16	560.78	552.16	543.29	
Dipole moment	7.839	7.818	3.833	3.876	4.328
Heat capacity, Cv	449.24	443.09	446.14	439.99	

**Table 6:** Global chemical descriptors (eV) calculated in the monomer and trimer forms of M6CU with different DFT methods and the 6-311++G(3df,pd) basis set, and in uracil, 6-CU and 3MU molecules.

Descriptor	Monomer	Trimer, A-Form		Trimer, B-Form		Uracil	6-CU	3MU
	B3LYP	B3LYP	CAM- B3LYP	B3LYP	CAM- B3LYP	B3LYP		
HOMO	−7.35	−6.83	−8.30	−6.97	−8.44	−7.32	−7.50	−7.16
LUMO	−1.74	−2.10	−0.76	−1.96	−0.63	−1.69	−1.91	−1.50
IP	7.35	6.83	8.30	6.97	8.44	7.32	7.50	7.16
EA	1.74	2.10	0.76	1.96	0.63	1.69	1.91	1.50
E_g_	5.61	4.73	7.54	5.01	7.81	5.63	5.59	5.66
χ	4.54	4.46	4.53	4.46	4.53	4.50	4.70	4.33
η	2.80	2.37	3.77	2.50	3.90	2.82	2.79	2.83
S	0.36	0.42	0.27	0.40	0.26	0.36	0.36	0.35

**Table 7:** Calculated harmonic wavenumbers (ν^cal^, cm^−1^), relative infrared intensities (A, %), relative Raman intensities (S, %), and Raman depolarization rations for plane (P) and unpolarized (U) incident light and force constants (f, mDyne/Å) obtained in M6CU at two different DFT levels in the gas phase (isolated state) of M6CU, together with the scaled values (ν^scal^, cm^−1^) and characterization according to the notation of Ref. [46].

Calculated								Scaled		Experimental		Ring n^o^	Characterization ^c^ (PED %)
B3LYP ^a^					M062X ^b^			B3LYP	M062X	IR	Raman		
ν^cal^	A	S	P	U	ν^cal^	A	S	ν^scal^	ν^scal^	ν^exp^	ν^exp^		
3634	13	36	0.17	0.29	3670	17	53	3473	3484			30	ν(N1-H) (99%)
3259	1	38	0.24	0.38	3276	1	60	3131	3104		3088 vw	28	ν(C5-H) (100%)
3182	0	15	0.72	0.84	3239	0	29	3060	3069	3088 m			ν_as_(C-H) in CH_3_ (100%)
3127	1	37	0.75	0.86	3177	1	53	3009	3009	3048 br	3021 vvw		ν_as_(C-H) in CH_3_ (100%)
3065	2	100	0.04	0.08	3103	2	100	2952	2938	2962 vw	2964 vw		ν_s_(C-H) in CH_3_ (100%)
1787	39	25	0.10	0.19	1879	32	28	1750	1759	1730 s	1707 w	26	ν(C2=O) (62%) + ν(C4O) (16%) + δ(N1H) (10%) + δ_s_(CH)CH_3_ (10%)
1731	100	23	0.28	0.44	1835	100	19	1696	1716	1704 s	1612 w	25	ν(C4=O) (61%) + ν(C2=O) (20%) + δ(C5-H) (13%)
1658	23	18	0.08	0.16	1720	25	23	1626	1605	1615, 1599 vs	1601 m	24	ν(C=C) (42%) + δ(N1-H) (15%) + δ(C5-H) (12%) + ν(C4=O) (10%)
1508	6	7	0.49	0.66	1527	44	9	1481	1420	1505 vs	1509 m		δ_as_(C-H) in CH_3_ (93%)
1497	1	3	0.75	0.86	1524	3	15	1470	1417		1464 vvw		δ_as_(C-H) in CH_3_ (92%)
1488	18	3	0.55	0.71	1508	1	14	1462	1401	1445 vs		21	ν(N1-C6) (44%) + δ(N1H) (29%) + ν(ring) (15%) + δ_s_(CH) CH_3_ (12%)
1458	10	2	0.74	0.85	1474	12	1	1433	1431				δ_s_(C-H) in CH_3_ (85%)
1392	21	0	0.70	0.82	1439	8	3	1369	1397	1387 s	1387 w	21	ν(C2-N-C4) (70%) + ν(N-C3) (20%) + δ_s_(C-H) in CH_3_ (10%)
1323	1	0	0.74	0.85	1328	4	13	1302	1290	1332 s	1336 vvw	23	δ(N1-H) (40%) + δ(C5-H) (38%) + δ(ring) (15%)
1280	2	5	0.35	0.52	1321	0	1	1261	1283	1277 vw	1279 vs	19	ν(N3C4) (32%) + δ_as_(CH)CH_3_ (31%) + δ(N1H) (13%) + δ(C5H) (13%)
1168	5	4	0.32	0.48	1206	3	2	1152	1172	1171 w	1171 sh	18	ν(N3-C3) (40%) + ν(C2-N1) (30%) + δ(C5-H) (24%)
1157	0	0	0.75	0.86	1168	0	9	1141	1135	1158 w	1156 vs		γ_as_(C-H) in CH_3_ (91%)
1150	1	6	0.27	0.43	1162	0	2	1133	1129	1110 w	1112 s	17	v(ring) (83%) + δ(C5-H) (10%)
1104	0	2	0.14	0.25	1123	1	5	1089	1092		1093 vvw	16	δ(ring) (47%) + δ(N1-H) (23%) + δ(C5-H) (21%)
993	4	1	0.62	0.76	1026	3	3	980	998	989 m	991 vvw		r(C-H) in CH_3_ (40%) + 16, δ(ring) (37%)
959	8	0	0.62	0.77	972	8	0	947	946	954 m		14	ν(ring) (91%)
836	4	1	0.75	0.86	818	5	2	826	797	847 s	846 w	13	γ(C5-H) (55%) + γ(C4-C5) (27%) + γ(C4=O) (10%)
765	2	0	0.75	0.86	767	0	4	756	747	754 vs	752 s		ν(C-Cl) (35%) + ν(ring) (25%) + γ(C-H) in CH_3_ (20%)
744	0	4	0.06	0.11	754	4	0	735	735		720 vvw	11	γ(C2=O) (59%) + γ(N1-C-N-C4) (22%) + 10, γ(C4=O) (21%)
725	1	0	0.75	0.86	727	2	1	716	709	717 vw		10	γ(C4=O) (38%) + γ(C5-H) (36%) + γ(ring) (15%)
644	1	0	0.72	0.83	649	1	1	636	633	653 vs	655 vvw	6	r(ring) (85%) + r(C-H) in CH_3_ (12%)
609	2	0	0.75	0.86	609	2	0	602	595		597 vs		γ(N1-C-C5) (41%) + 8, γ(N1-H) (30%) + γ(ring) (27%)
580	1	9	0.09	0.16	595	0	10	573	581			12	ν(ring) (65%) + ν(C-Cl) (11%)
536	1	2	0.39	0.56	536	1	3	529	524		539 m	7	δ_as_(ring) (83%)
492	66	0	0.75	0.86	493	9	3	486	482			8	γ(N1-H) (64%) + γ(ring) (23%)

^a^ With the 6-311++G(3df,pd) basis set. ^b^ With the 6-31G(d,p) basis set. ^c^ Characterization obtained with B3LYP. PED less than 10% was excluded. Abbreviations: ν, stretching; δ, in-plane bending; γ, out-of-plane bending; r, rocking.

**Table 8:** Scaled wavenumbers (ν^scal^, cm^−1^) in the 4000–500 cm^−1^ range determined with the PSE procedure at the B3LYP/6-311++G(3df,pd) and CAM-B3LYP/6-311++G(3df,pd) levels in the solid state (trimer form) of M6CU in the *A*-form, together with the relative IR intensities (A, %), and Raman relative intensities (S, %). Among the three wavenumbers, the one with the highest IR intensity is shown in bold, and the one with the highest Raman intensity in *italics*. Comparison with the experimental wavenumbers (ν^exp^) in the solid state with the IR and Raman intensities. The main characterization with the ring mode number [46] and the %PED in parenthesis.

B3LYP			CAM-B3LYP			Experimental		Ring n^o^	Assignment
ν^scal^	A	S	ν^scal^	A	S	IR	Raman		
3467 *, 3119, ***3103*** 3130, ***3091***, 3090 3067, 3065, *3058* *3013*, 3009, 3005 **2956**, *2953*, 2949 1751 *, **1735**, *1725* 1684 *, 1656, ***1643*** 1621 *, ***1619***, 1614 1522, ***1520***, 1484 1479, 1478, 1471 1470, 1469 *, 1466 * 1441, 1437 *, 1436 * 1385, 1378 *, 1376 * 1347, 1333 *, 1320 * 1269, 1266 *, 1263 * 1176, 1166 *, 1161 * 1152, 1141 * 1140 *1107*, 1102 *, 1094 * 991, 988 *, 983 * 951 *, 950, 945 * 888, 880, 858 * 848, 820 * 765, 763 *, 758 * 749*, 743* *, 741 * 732 *, 719, 699 * 653, 644 *, 642 * 606, 604 *, **601** * 589, 585 *, **577** * 538, 534 *, *532* *	100 0 0 0 1 12 72 8 5 0 3 4 5 1 0 1 0 0 0 2 3 3 1 0 0 0 1 0 1 4	100 78 5 9 24 16 20 4 13 1 1 1 0 2 4 4 4 0 3 0 0 0 0 0 2 0 0 0 4 1	3480 *, 3134, ***3113*** **3096**, *3089*, 3088 3074, 3072, 3065 *3024*, 3020, 3016 *2961*, 2960, **2956** 1767 *, 1751, *1739* 1700 *, 1673, ***1658*** 1639 *, *1638*, 1632 1526, ***1523***, 1486 1470, 1468, 1461 *1457*, 1456 1445, **1439**, *1435* 1392, **1387** 1346, ***1335***, 1313 *1276*, 1275, 1270 1181, 1170, *1168* *1163*, 1154, 1148 1133 *1110*, 1104, **1096** 993, **990**, 985 **958**, 956, 952 891, 885, 856 **849**, 825 772, 768, **765** 752, *747*, 744 733, 721, 699 **650**, 641, 639 607, 605, 603 *591*, 587, **580** 534, 529, 527	10 100 0 0 1 13 77 15 7 1 0 7 2 1 0 1 0 0 1 2 3 3 1 1 0 0 2 0 1 5	9 100 3 6 16 10 12 6 8 1 1 1 0 1 5 2 4 0 2 0 0 0 0 0 2 0 0 0 3 1	3088 m 3048 br 2962 vw 1730 s 1704 s 1615, 1599vs 1505 vs 1445 vs 1387 s 1332 s 1277 vw 1171 w 1158 w 1110 w 989 m 954 m 847 s 754 vs 717 vw 653 vs	3088 vw 3021 vvw 2964 vw 1707 w 1636 vw 1612,1601 m 1509 m 1464 vvw 1387 w 1336 vvw 1279 vs 1171 sh 1156 vs 1112 s 1093 vvw 991 vvw 846 w 752 s 720 vvw 655 vvw 597 vs 539 m	30 28 26 25 24 23 21 19 18 17 16 14 13 8 11 12 10 6 4 5 7	ν(N1-H) (100%) ν(C5-H) (75%) + ν(N1-H) (25%) ν_as_(C-H) in CH_3_ (100%) ν_as_(C-H) in CH_3_ (100%) ν_s_(C-H) in CH_3_ (100%) ν(C2=O) (57%) + ν(C4=O) (29%) + δ(N1-H) (10%) ν(C4=O) (55%) + ν(C2=O) (20%) + δ(C5-H) (16%) ν(C=C)(50%)+δ(N1H)(20%)+δ(C5H)(12%)+ν(C4O)(11%) δ(N1-H) (48%) + ν(N1-C6) (29%) + ν(C2=O) (13%) δ_as_(C-H) in CH_3_ (87%) + δ(N1-H) (10%) δ_as_(C-H) in CH_3_ (100%) δ_s_(C-H) in CH_3_ (57%) + ν_as_(C-N3-C) (28%) ν(CN3C)(34%)+δ_s_(CH_3_)(30%)+ν(NC3)(18%)+δ(N1H)(17%) δ(C5-H) (40%) + δ(N1-H) (29%) + ν(C-N1-C) (21%) ν(N3C4)(51%)+γ_s_(CH_3_)(23%)+δ(N1H)(11%)+δ(C5H)(10%) ν(N3C3)(28%)+δ(C5H)(27%)+ν_as_(NC6C)(17%)+ν(C4C5)(10%) v(N1-C2) (38%) + ν(C4-C5) (26%) + ν(N3-C3) (21%) γ_as_(C-H) in CH_3_ (99%) δ(ring) (54%) + δ(C5-H) (22%) + δ(N1-H) (20%) r(C-H) in CH_3_ (60%) + 16, δ(ring) (32%) ν_as_(ring) mainly in N1-C6-C (70%) + ν(C-Cl) (25%) γ(C5-H) (58%) + γ(N1-H) (37%) γ(N1-H) (68%) + γ(C5-H) (27%) γ(ring) mainly in C2=O (96%) ν(ring) (65%)+ ν(C-Cl) (18%) + γ(C-H) in CH_3_ (16%) γ(C4=O)(38%)+γ(C2=O)(24%)+γ(C5-H)(23%)+γ(N1-H)(15%) r(ring) (82%) + r(C-H) in CH_3_ (11%) γ(N-C5-C) (75%) + γ(ring) (19%) ν(C-Cl) (49%) + δ_as_(ring) (46%) δ(ring) (99%)

* Values mainly corresponding to rings *I* and *III*.

**Table 9:** Scaled wavenumbers (ν^scal^, cm^−1^) in the far-IR region (< 500 cm^−1^) determined with the PSE procedure at the B3LYP/6-311++G(3df,pd) and CAM-B3LYP/6-311++G(3df,pd) levels in the trimer of the *A*-form of M6CU, together with the relative IR intensities (A, %), and Raman relative intensities (S, %). Among the three wavenumbers, the one with the highest IR intensity is shown in bold, and the one with the highest Raman intensity in *italics*. Comparison with the experimental wavenumbers (ν^exp^) in the solid state with the IR and Raman intensities. The main characterization with the ring mode number and the %PED in parenthesis.

B3LYP			CAM-B3LYP			experimental	Ring n^o^	Assignment
ν^scal^	A	S	ν^scal^	A	S	Raman		
513 *, 425, 419 *,407 * 371, 368 *, 365 * 353, 347 *, 344 * 266, 262 *, 260 * 246, 240 *, *238* * 181, 169, *165* 155, 150 *, 144 * 99, 95 * 98, 72 *, 57 69 *, *61*, 44 * 57, 42, 33 15 33, 13 12, 3	1 0 1 0 0 0 0 0 0 0 0 0	0 1 0 0 0 0 0 0 0 0 1 0	510, 423, 416, 404 *369*, 367, 364 **350**, 343, 340 261, 257, 254 243, 237, 235 177, 165, 160 152, 147, 142 100, 96 94, 92, *71* 67, 59, *55* 53, 43, 39 31 30, 12 9, 8	1 0 1 0 0 0 0 0 0 0 0 0 0 0	0 1 0 0 0 0 0 0 0 1 0 0 0 0	380 s 254 w 211 s 105 vs 82 s	3 5 1 2 1	δ(OCNCO) mainly C4=O (82%) + δ(C-H) in CH_3_ (14%) δ(C-Cl) (80%) + δ(ring) (15%) δ(C4=O) (40%) + δ(N3-C3) (32%) + δ(C2=O) (15%) τ(ring) on C2-N3-C4 axis (86%) δ(ring) (51%) + δ(C-Cl) (45%) Puckering N1 (100%) Puckering on O2, O4 (94%) puckering on N3 (73%) + γ(CH_3_) (25%) r(ring) (85%) + r(C-H) in CH_3_ (12%) Γ(CH_3_) (100%) H-bond shearing Cogwheel Butterfly Torsion (twist)

* Values mainly corresponding to rings *I* and *III*.

## Data Availability

The original contributions presented in this study are included in the article/Supplementary Materials. Further inquiries can be directed to the corresponding authors.

## Supplementary Materials

The following supporting information can be downloaded at: https://www.mdpi.com/article/10.3390/molecules31152665/s1, Figure S1: Comparison of the theoretical scaled Raman spectra in the 3600-0 cm^−1^ range by the PSE procedure with the corresponding experimental spectrum in the solid state. (a) In the isolated state (monomer form) by B3LYP and M06-2X methods with the 6-31G(d,p) basis set. (b-c) In the trimer form by B3LYP and CAM-B3LYP methods with the 6-311++G(3df,pd) basis set. (d) Experimental Raman spectrum. Figure S2: Comparison of the theoretical scaled IR spectra in the 3600-400 cm^−1^ range by the PSE procedure with the corresponding experimental one. (a) In the isolated state (monomer form) by B3LYP and M06-2X methods with the 6-31G(d,p) basis set. (b-c) In the trimer form by B3LYP and CAM-B3LYP methods with the 6-311++G(3df,pd) basis set. (d) Experimental spectrum in KBr pellets. Figure S3: Comparison of the theoretical scaled Raman spectra in the 3600-2800 cm^−1^ range by the PSE procedure with the corresponding experimental one. (a) In the isolated state at the B3LYP/6-31G(d,p) level. (b) In the isolated state at the B3LYP/6-311++G(3df,pd) level. (c) Experimental spectrum in the solid state. Figure S4: Comparison of the theoretical scaled Raman spectra in the 1800-1000 cm^−1^ range by the PSE procedure with the corresponding experimental one. (a) In the isolated state at the B3LYP/6-31G(d,p) level. (b) In the isolated state at the B3LYP/6-311++G(3df,pd) level. (c) Experimental spectrum in the solid state. Figure S5: Comparison of the theoretical scaled Raman spectra in the 1800-1000 cm^−1^ range by the PSE procedure with the corresponding experimental one. (a) In the isolated state at the B3LYP/6-31G(d,p) level. (b) In the isolated state at the B3LYP/6-311++G(3df,pd) level. (c) Experimental spectrum in the solid state. Figure S6: Comparison of the theoretical scaled IR spectra in the 3600-2500 cm^−1^ range by the PSE procedure with the corresponding experimental one. (a) In the isolated state (monomer form) by B3LYP and M06-2X methods with the 6-31G(d,p) basis set. (b) In the isolated state at the B3LYP/6-31G(d,p) level. (c) In the isolated state at the B3LYP/6-311++G(3df,pd) level. (d) Experimental spectrum in KBr pellets. Figure S7: Comparison of the theoretical scaled IR spectra in the 1800-1000 cm^−1^ range by the PSE procedure with the corresponding experimental one. (a) In the isolated state (monomer form) by B3LYP and M06-2X methods with the 6-31G(d,p) basis set. (b) In the isolated state at the B3LYP/6-31G(d,p) level. (c) In the isolated state at the B3LYP/6-311++G(3df,pd) level. (d) Experimental spectrum in KBr pellets. Figure S8: Comparison of the theoretical scaled IR spectra in the 1000-400 cm^−1^ range by the PSE procedure with the corresponding experimental one. (a) In the isolated state (monomer form) by B3LYP and M06-2X methods with the 6-31G(d,p) basis set. (b) In the isolated state at the B3LYP/6-31G(d,p) level. (c) In the isolated state at the B3LYP/6-311++G(3df,pd) level. (d) Experimental spectrum in KBr pellets. Figure S9: Comparison of the theoretical scaled Raman spectra in the 3600-0 cm^−1^ range by the PSE procedure with the corresponding experimental spectrum in the solid state: (a) In the *A*-trimer form by B3LYP and CAM-B3LYP methods with the 6-311++G(3df,pd) basis set. (b) In the *A*-trimer form at the B3LYP-D3/6-311++G(3df,pd) level. (c) Experimental Raman spectrum. Figure S10: Comparison of the theoretical scaled IR spectra in the 3600-400 cm^−1^ range by the PSE procedure with the corresponding experimental spectrum in KBr pellets: (a) In the *A*-trimer form by B3LYP and CAM-B3LYP methods with the 6-311++G(3df,pd) basis set. (b) In the *A*-trimer form at the B3LYP-D3/6-311++G(3df,pd) level. (c) Experimental IR spectrum. Figure S11: Experimental Raman spectrum in the 3100-2600 cm^−1^ range of M6CU. Figure S12: Comparison of the theoretical scaled IR spectra in the 1800-1000 cm^−1^ range by the PSE procedure with the corresponding experimental spectrum in KBr pellets: (a) In the *A*-trimer form by B3LYP and CAM-B3LYP methods with the 6-311++G(3df,pd) basis set. (b) In the *A*-trimer form at the B3LYP-D3/6-311++G(3df,pd) level. (c) Experimental IR spectrum. Figure S13: Two views of the M6CU effect inserted into the optimized double strand 5′-UUU-3′ microhelix at the M06-2X/6-31G(d,p) level in the A-type. The values of the intermolecular H-bonds are included. (a) With M6CU values in plane n+1. (b) With the deformation in all planes. Table S1: Geometrical parameters, bond lengths (in A) and angles (in degrees) of M6CU at different theoretical methods and basis set. Table S2: NBO total atomic charges on the atoms of M6CU with different DFT methods and basis set. Table S3: Theoretical computed total energies (A.U.), Gibbs energies (A.U.), rotational constants (GHz), entropies (J mol^−1^ K^−1^), dipole moments (Debyes), and constant volume heat capacity (C_v_, J mol^−1^ K^−1^) in the monomer form of M6CU, uracil, 6-CU and 3MU molecules with different methods and basis set. Table S4: Global chemical descriptors (eV) calculated in the monomer and trimer forms of M6CU at different theoretical levels, and in uracil, 6-CU and 3MU molecules with the MP2 method. Table S5: Calculated harmonic wavenumbers (ν^cal^, cm^−1^) in the isolated state (monomer form), relative infrared intensities (A, %), relative Raman intensities (S, %), Raman depolarization rations for plane (P) and unpolarized (U) incident light and force constants (f, mDyne/Å) obtained in M6CU with two different DFT methods and the 6-31G(d,p) basis set, together with the scaled values (ν^scal^, cm^−1^) and its characterization according to notation of ref. [46]. Table S6: Calculated harmonic wavenumbers (ν^cal^, cm^−1^) in low modes of the isolated state (monomer form), relative infrared intensities (A, %), Raman activities (S, %), Raman depolarization rations for plane (P) and unpolarized (U) incident light and force constants (f, mDyne/Å) obtained in M6CU with two different DFT methods and the 6-31G(d,p) and 6-311++G(3df,pd) basis set, together with the scaled values (ν^scal^, cm^−1^) and its characterization according to notation of ref. [46]. Table S7: Scaled wavenumbers (ν^scal^, cm^−1^) in the 4000-500 cm^−1^ range determined with the PSE procedure at the CAM-B3LYP/6-311++G(3df,pd) levels in the solid state (trimer form) of M6CU in *A*- and *B*-forms, together with the relative IR intensities (A, %), and Raman relative intensities (S, %). Comparison with the experimental wavenumbers (ν^exp^) in the solid state with the IR and Raman intensities. The main characterization with the ring mode number and the %PED in parenthesis. Table S8: Scaled wavenumbers (ν^scal^, cm^−1^) in the far-IR region (< 500 cm^−1^) determined with the PSE procedure at the B3LYP/6-311++G(3df,pd) and CAM-B3LYP/6-311++G(3df,pd) levels in the trimer form of *A*- and *B*-forms in M6CU, together with the relative IR intensities (A, %), and Raman relative intensities (S, %). Comparison with the experimental wavenumbers (ν^exp^) in the solid state with the IR and Raman intensities. The main characterization with the ring mode number and the %PED in parenthesis.
