# Supplementary material for: Effects of Chlorine and Methyl Substitution on Structure, IR, and Raman Spectra of the Biomolecule 6-Chloro-3-methyluracil in Isolated and Solid States and in Base-Pair Formation
Source: Molecules. 2026 Jul 30;31(15):2665. doi: 10.3390/molecules31152665 (PMC13468333; doi:10.3390/molecules31152665)
Supplement: Supplementary file 1 [file molecules-31-02665-s001.zip › molecules-4431995-supplementary.pdf]

SUPPLEMENTARY MATERIAL

Molecules

The effect of the chlorine and methyl group on the structure and IR and Raman spectra of the biomolecule 6-chloro-3-methyluracil in the isolated and solid states, and in the base pair formation

M. Alcolea Palafox[a], Nataliya P. Belskaya[b], N. Hristova-Avakumova[c], Irena P. Kostova[d]

[a] Chemical Physics department. Chemistry Faculty. Universidad Complutense. Madrid- 28040. Spain. (e-mail: alcolea@ucm.es)

[b] Department of Technology for Organic Synthesis, Ural Federal University, 19 Mira Str., Yekaterinburg 620012, Russia; n.p.belskaya@urfu.ru

[c] Department of Medical Physics and Biophysics, Faculty of Medicine, Medical University of Sofia, Sofia, Bulgaria; nhristova@medfac.mu-sofia.bg

[d] Department of Chemistry, Faculty of Pharmacy, Medical University – Sofia, 2 Dunav Str., 1000 Sofia, Bulgaria. i.kostova@pharmfac.mu-sofia.bg

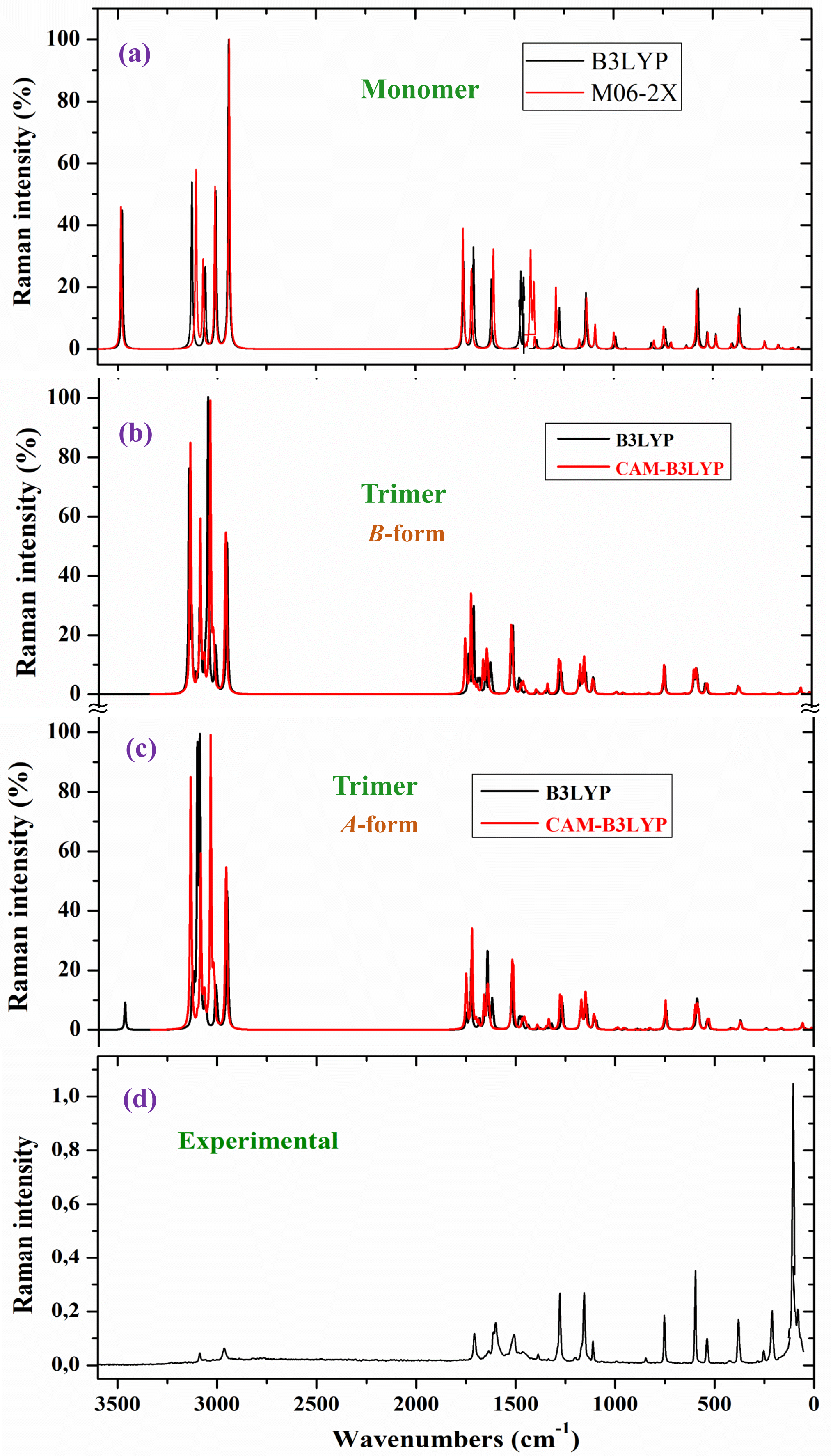

Figure S1. Comparison of the theoretical scaled Raman spectra in the 3600-0 cm^{-1} range by the PSE procedure with the corresponding experimental spectrum in the solid state. (a) In the isolated state (monomer form) by B3LYP and M06-2X methods with the 6-31G(d,p) basis set. (b-c) In the trimer form by B3LYP and CAM-B3LYP methods with the 6-311++G(3df,pd) basis set. (d) Experimental Raman spectrum.

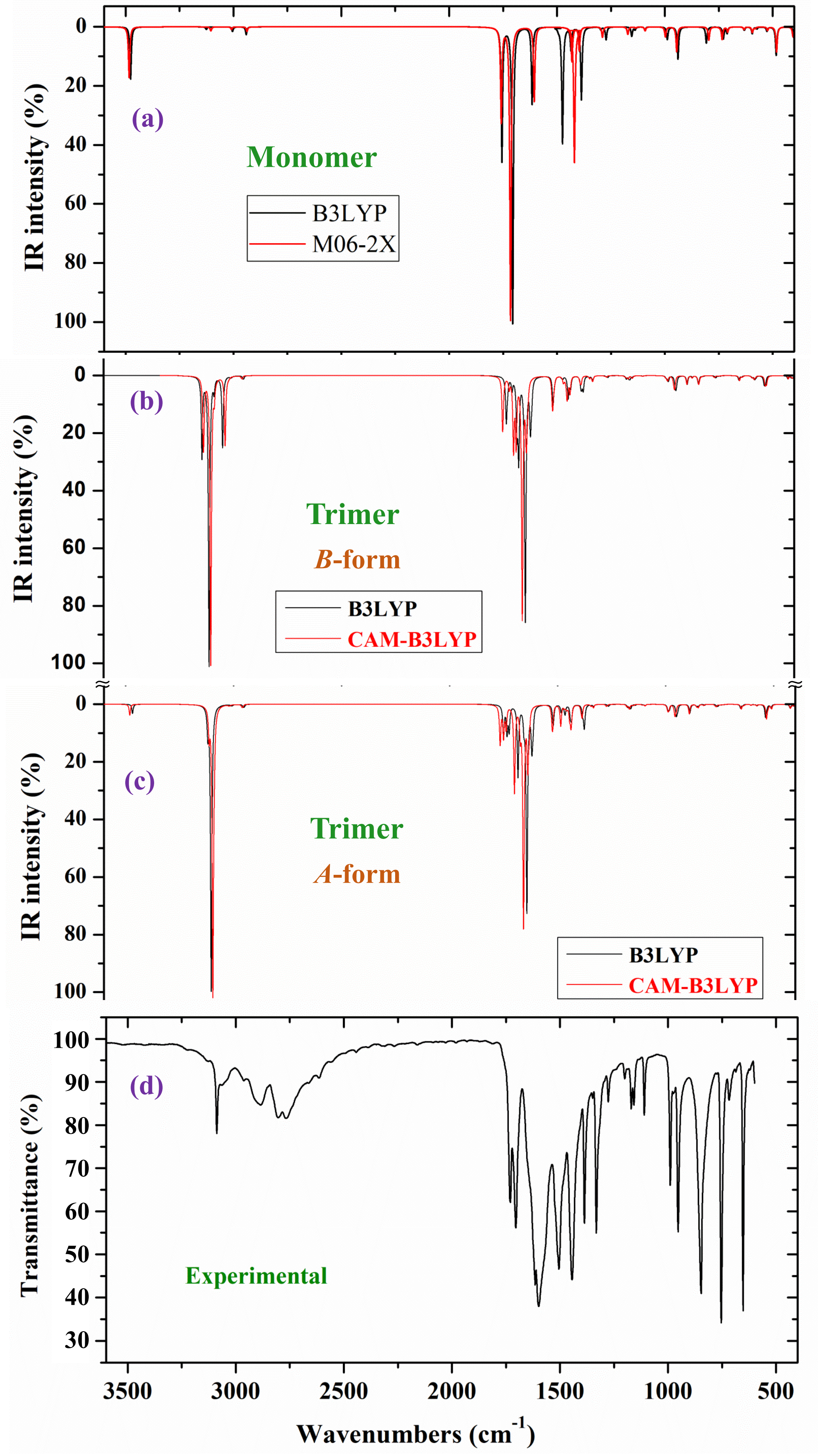

Figure S2. Comparison of the theoretical scaled IR spectra in the 3600-400 cm^{-1} range by the PSE procedure with the corresponding experimental one. (a) In the isolated state (monomer form) by B3LYP and M06-2X methods with the 6-31G(d,p) basis set. (b-c) In the trimer form by B3LYP and CAM-B3LYP methods with the 6-311++G(3df,pd) basis set. (d) Experimental spectrum in KBr pellets.

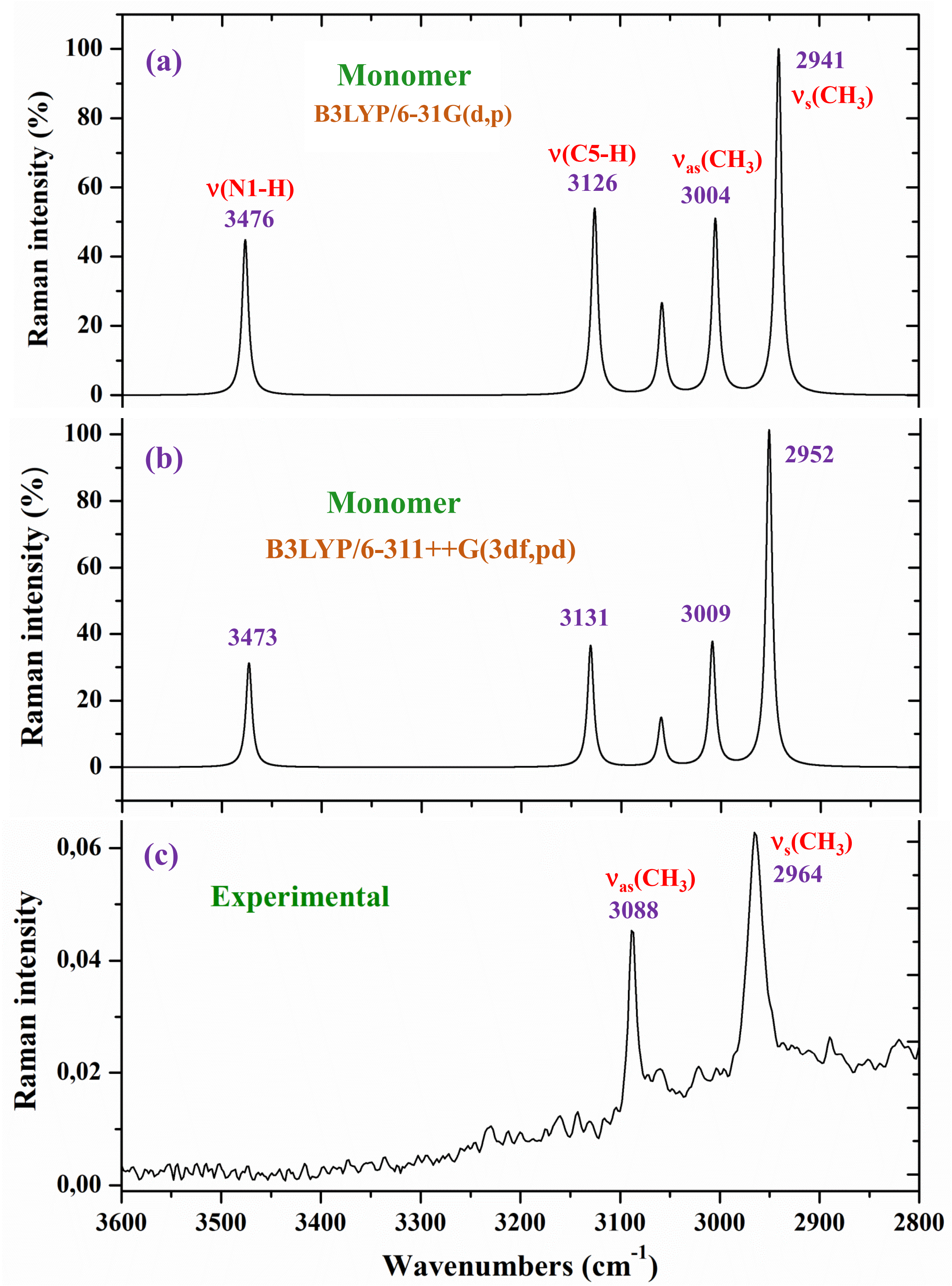

Figure S3. Comparison of the theoretical scaled Raman spectra in the 3600-2800 cm^{-1} range by the PSE procedure with the corresponding experimental one. (a) In the isolated state at the B3LYP/6-31G(d,p) level. (b) In the isolated state at the B3LYP/6-311++G(3df,pd) level. (c) Experimental spectrum in the solid state.

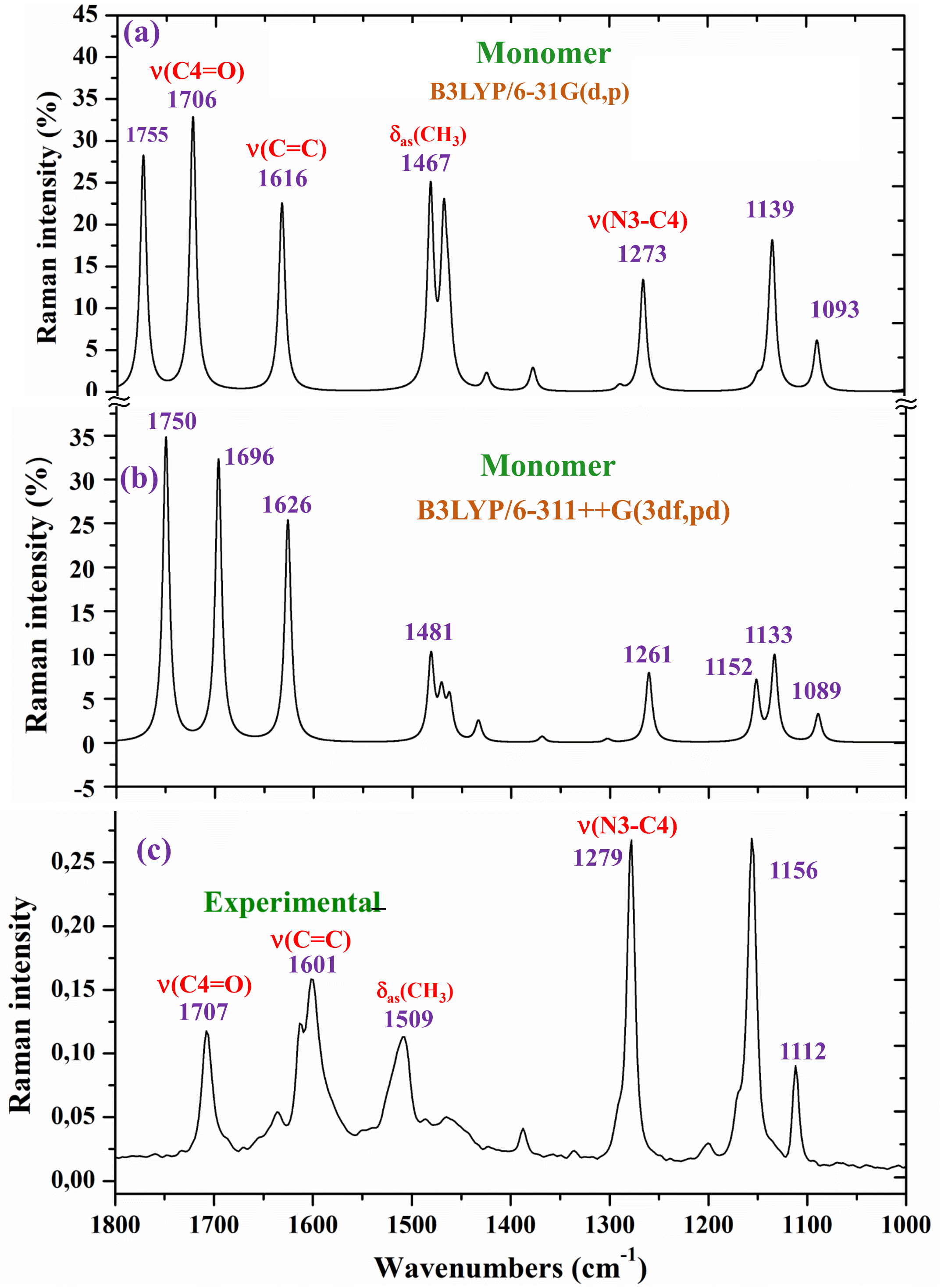

Figure S4. Comparison of the theoretical scaled Raman spectra in the 1800-1000 cm^{-1} range by the PSE procedure with the corresponding experimental one. (a) In the isolated state at the B3LYP/6-31G(d,p) level. (b) In the isolated state at the B3LYP/6-311++G(3df,pd) level. (c) Experimental spectrum in the solid state.

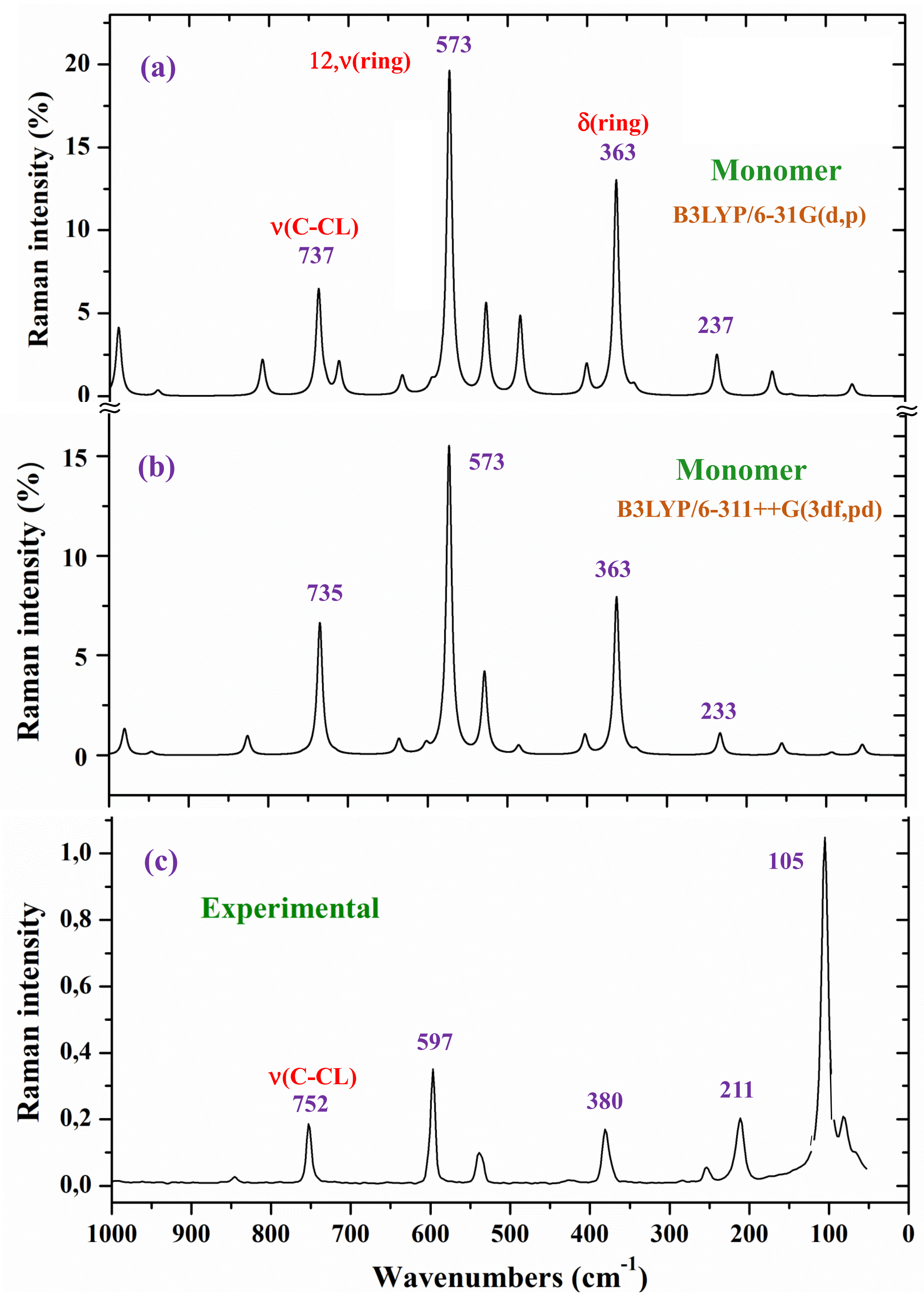

Figure S5. Comparison of the theoretical scaled Raman spectra in the 1800-1000 cm^{-1} range by the PSE procedure with the corresponding experimental one. (a) In the isolated state at the B3LYP/6-31G(d,p) level. (b) In the isolated state at the B3LYP/6-311++G(3df,pd) level. (c) Experimental spectrum in the solid state.

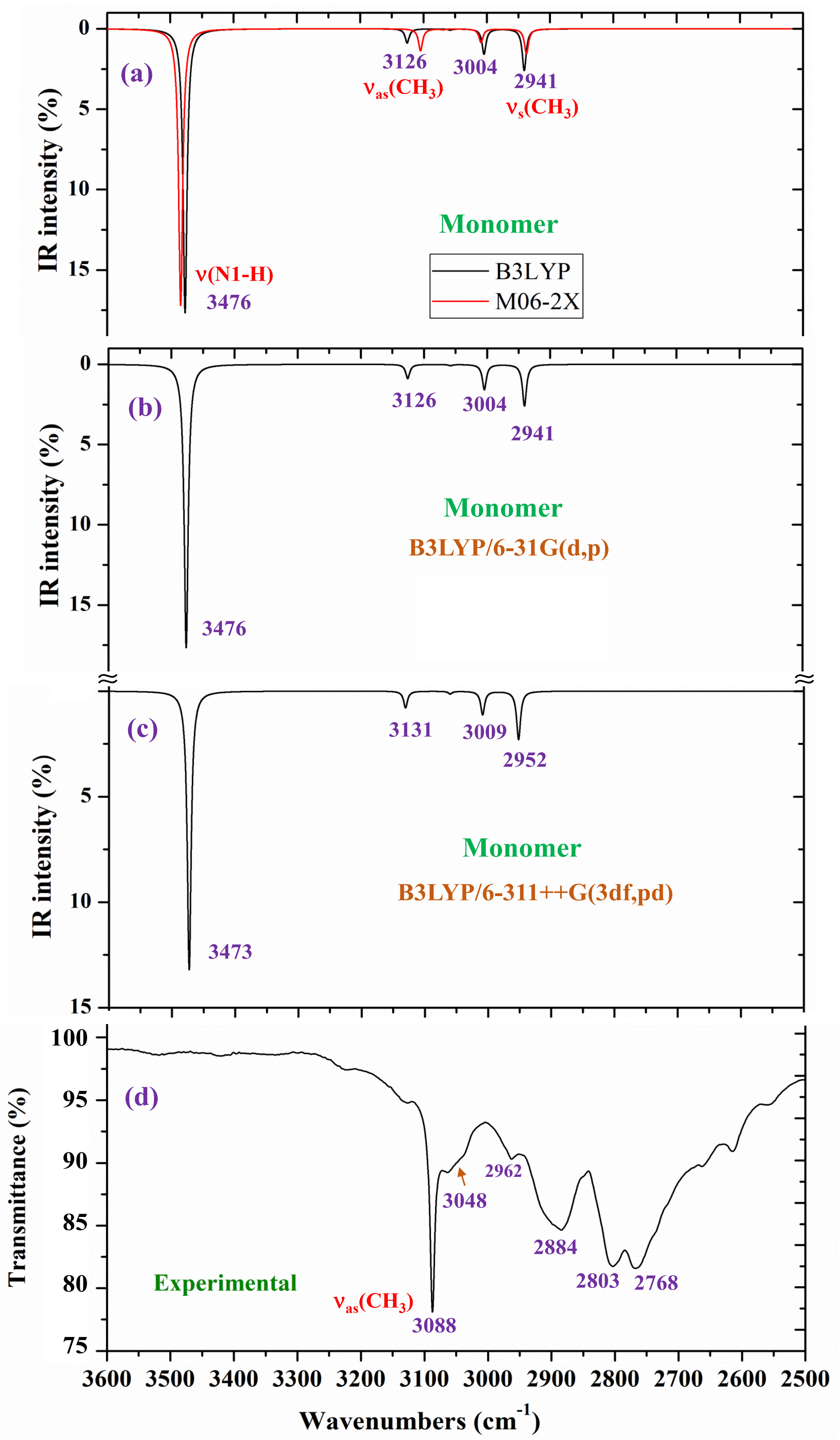

Figure S6. Comparison of the theoretical scaled IR spectra in the 3600-2500 cm^{-1} range by the PSE procedure with the corresponding experimental one. (a) In the isolated state (monomer form) by B3LYP and M06-2X methods with the 6-31G(d,p) basis set. (b) In the isolated state at the B3LYP/6-31G(d,p) level. (c) In the isolated state at the B3LYP/6-311++G(3df,pd) level. (d) Experimental spectrum in KBr pellets.

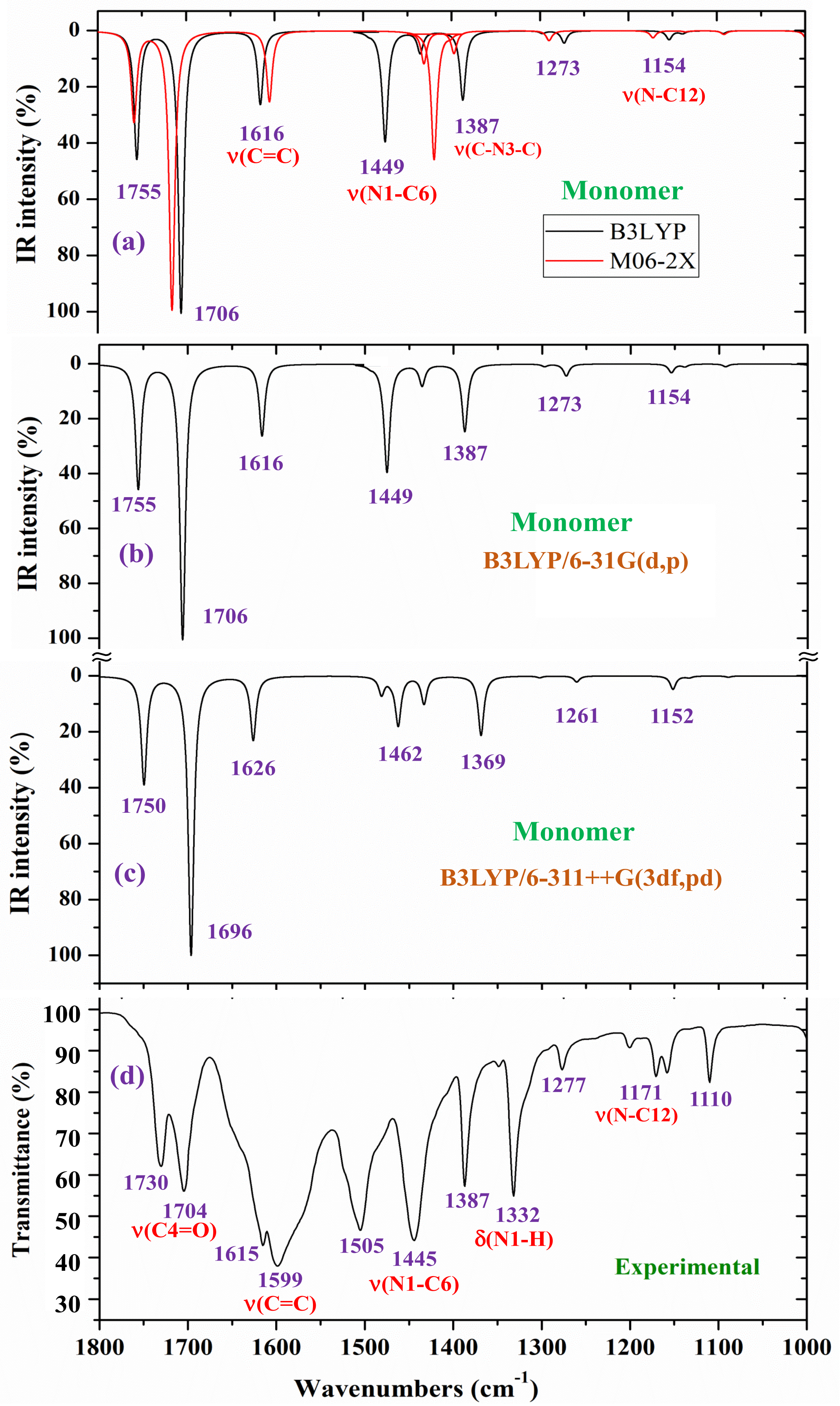

Figure S7. Comparison of the theoretical scaled IR spectra in the 1800-1000 cm^{-1} range by the PSE procedure with the corresponding experimental one. (a) In the isolated state (monomer form) by B3LYP and M06-2X methods with the 6-31G(d,p) basis set. (b) In the isolated state at the B3LYP/6-31G(d,p) level. (c) In the isolated state at the B3LYP/6-311++G(3df,pd) level. (d) Experimental spectrum in KBr pellets.

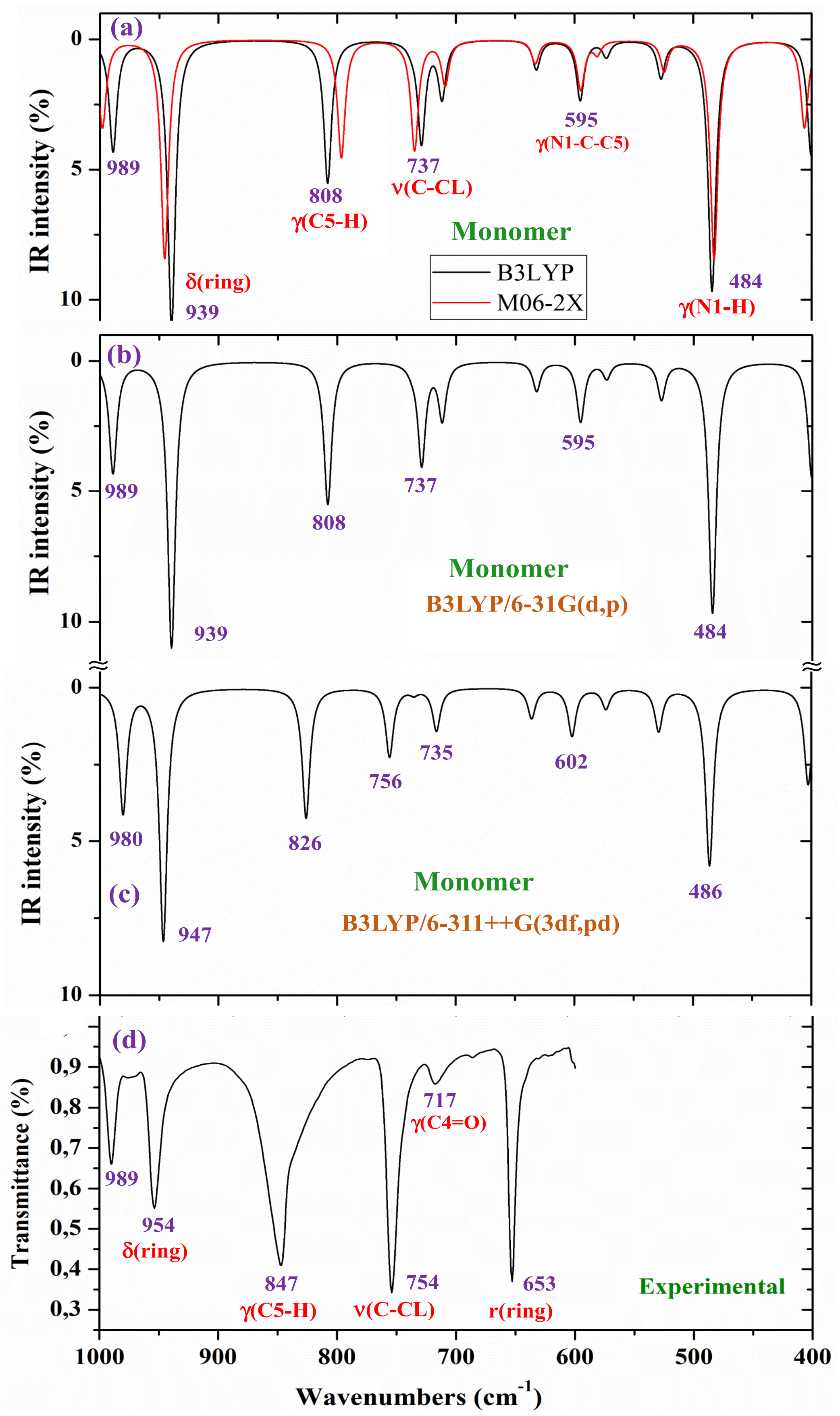

Figure S8. Comparison of the theoretical scaled IR spectra in the 1000-400 cm^{-1} range by the PSE procedure with the corresponding experimental one. (a) In the isolated state (monomer form) by B3LYP and M06-2X methods with the 6-31G(d,p) basis set. (b) In the isolated state at the B3LYP/6-31G(d,p) level. (c) In the isolated state at the B3LYP/6-311++G(3df,pd) level. (d) Experimental spectrum in KBr pellets.

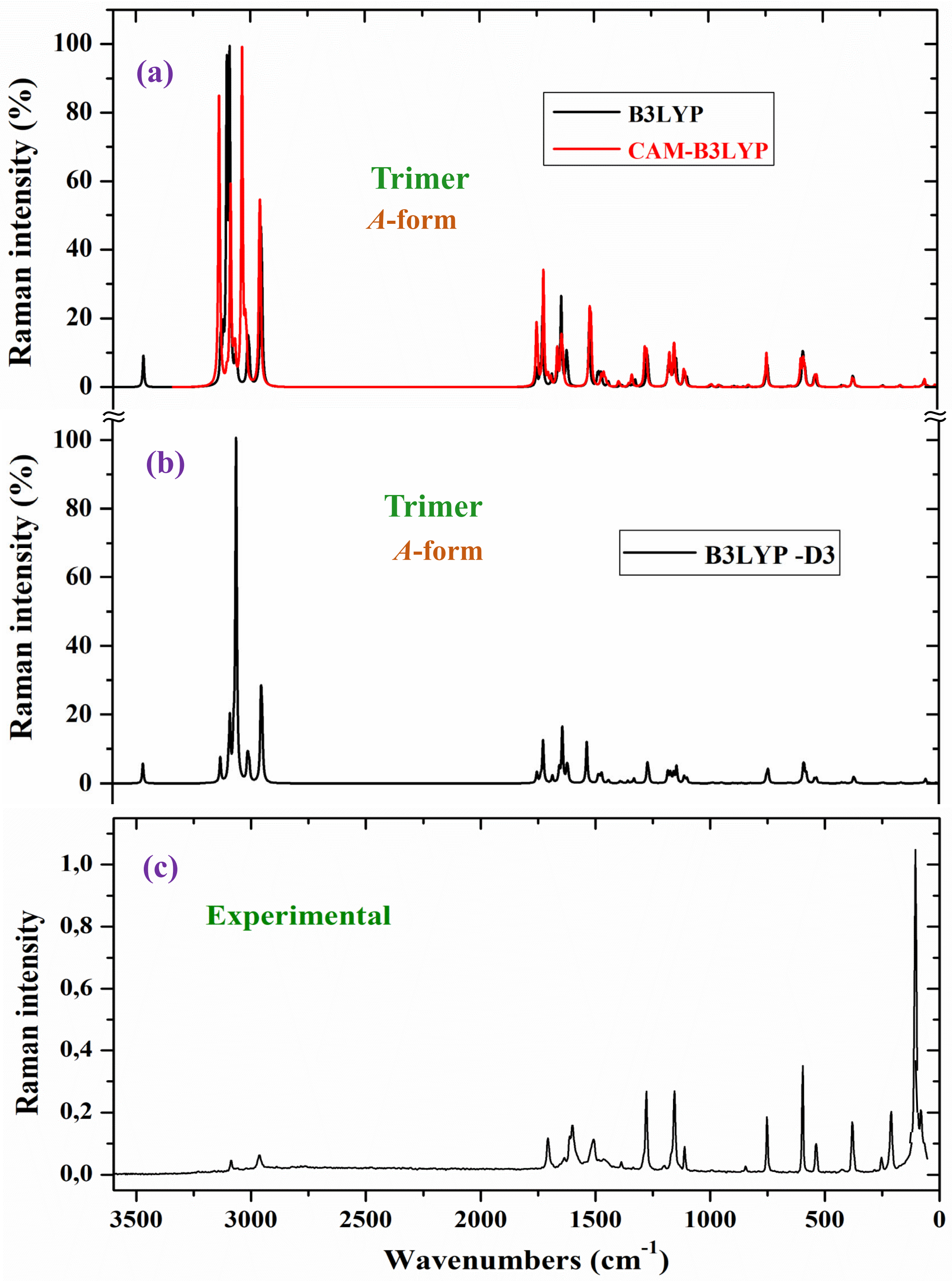

Figure S9. Comparison of the theoretical scaled Raman spectra in the 3600-0 cm^{-1} range by the PSE procedure with the corresponding experimental spectrum in the solid state: (a) In the *A*-trimer form by B3LYP and CAM-B3LYP methods with the 6-311++G(3df,pd) basis set. (b) In the *A*-trimer form at the B3LYP-D3/6-311++G(3df,pd) level. (c) Experimental Raman spectrum.

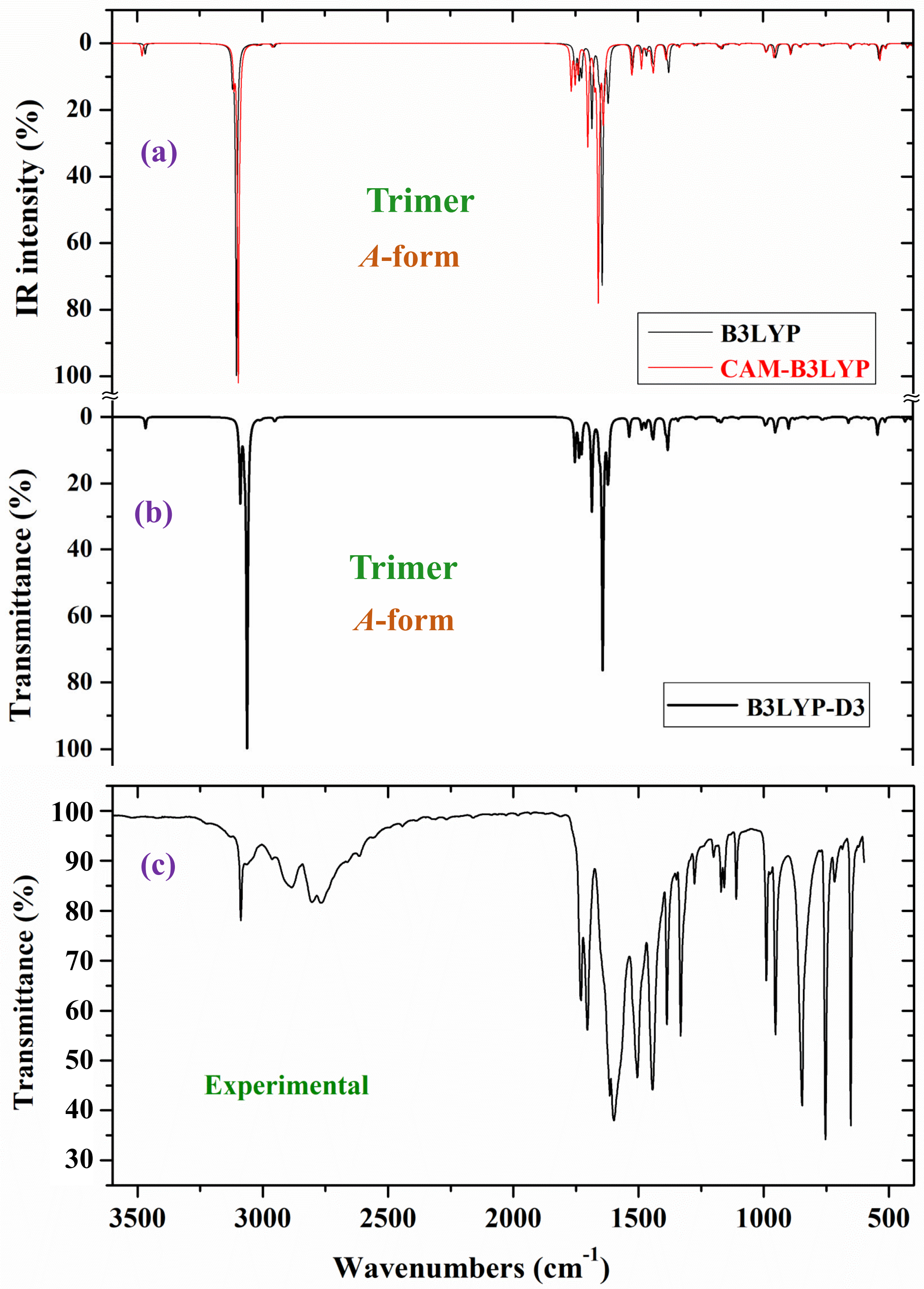

Figure S10. Comparison of the theoretical scaled IR spectra in the 3600-400 cm^{-1} range by the PSE procedure with the corresponding experimental spectrum in KBr pellets: (a) In the *A*-trimer form by B3LYP and CAM-B3LYP methods with the 6-311++G(3df,pd) basis set. (b) In the *A*-trimer form at the B3LYP-D3/6-311++G(3df,pd) level. (c) Experimental IR spectrum.

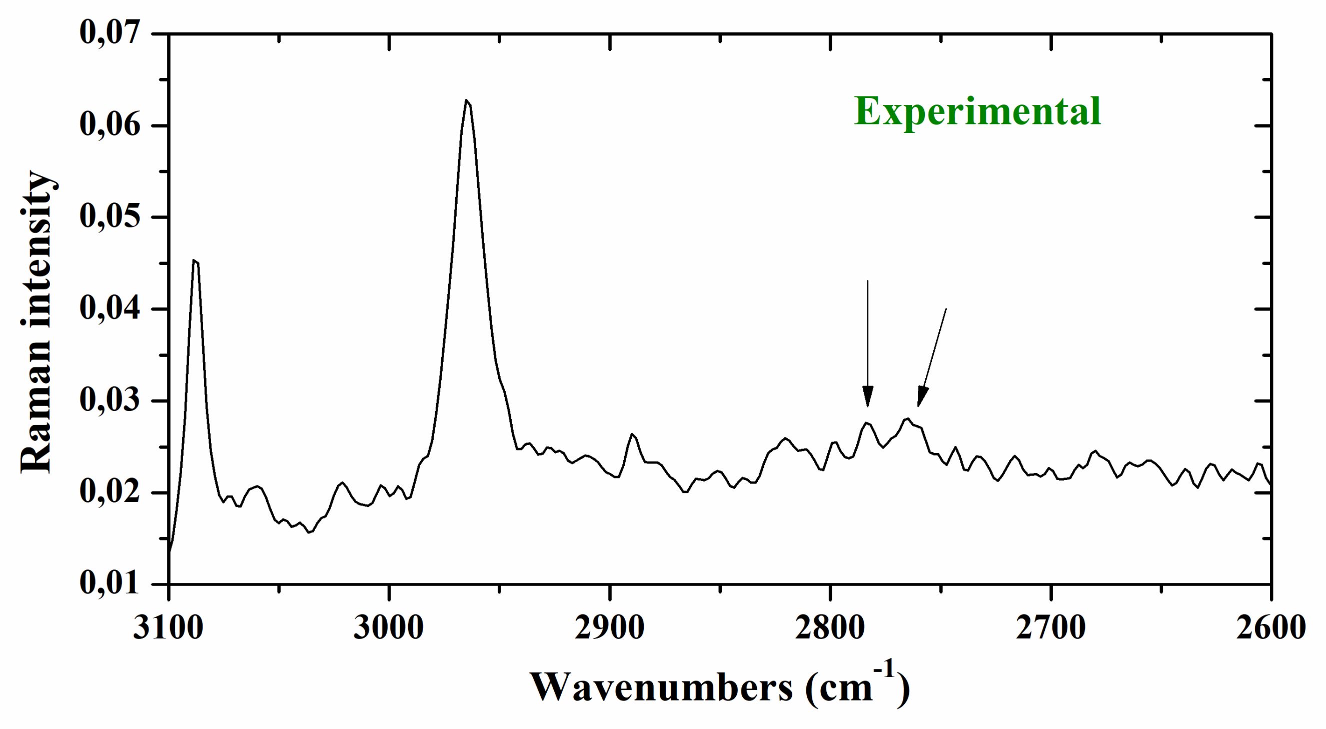

Figure S11. Experimental Raman spectrum in the 3100-2600 cm^{-1} range of M6CU.

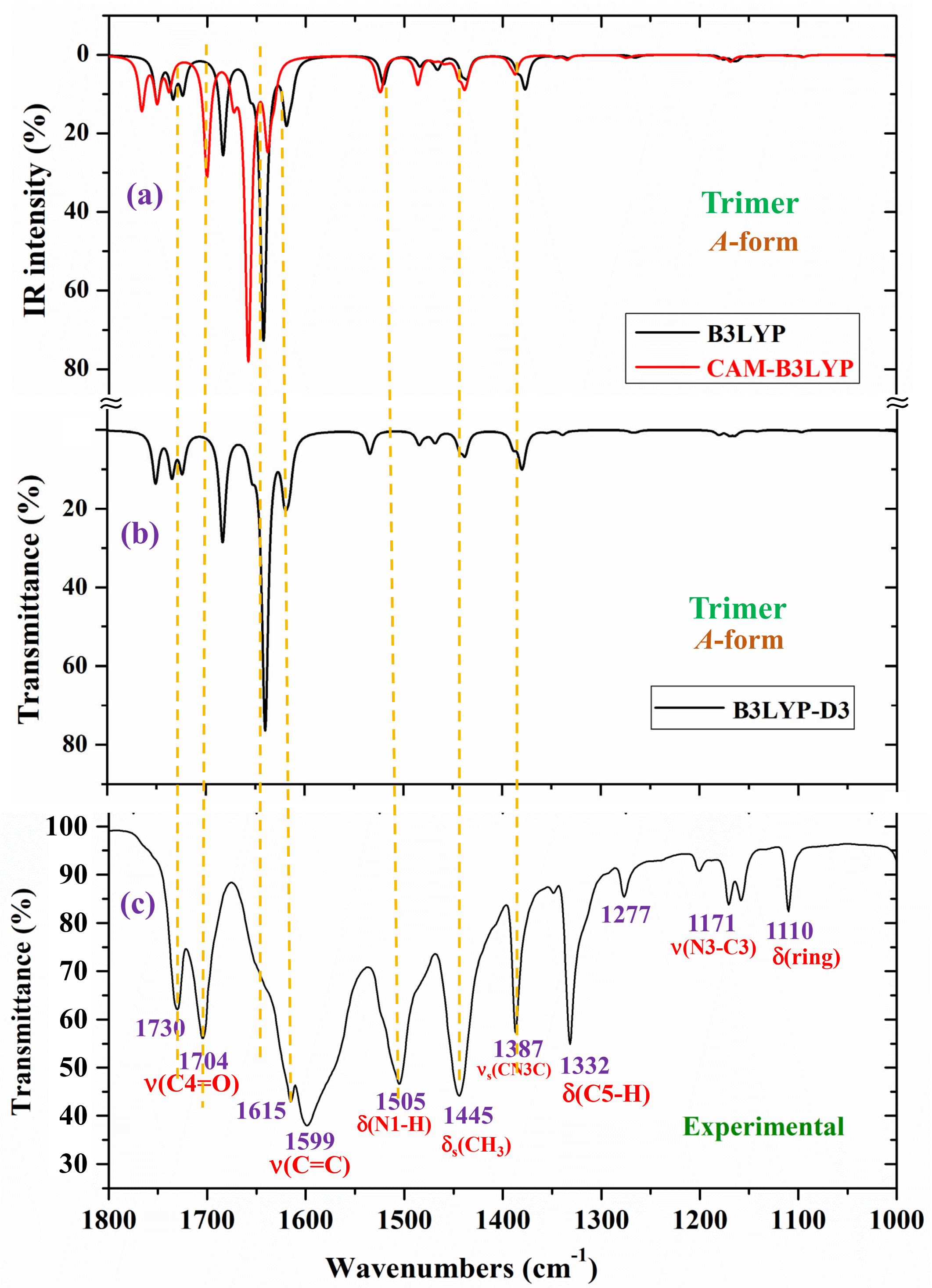

Figure S12. Comparison of the theoretical scaled IR spectra in the 1800-1000 cm^{-1} range by the PSE procedure with the corresponding experimental spectrum in KBr pellets: (a) In the *A*-trimer form by B3LYP and CAM-B3LYP methods with the 6-311++G(3df,pd) basis set. (b) In the *A*-trimer form at the B3LYP-D3/6-311++G(3df,pd) level. (c) Experimental IR spectrum.

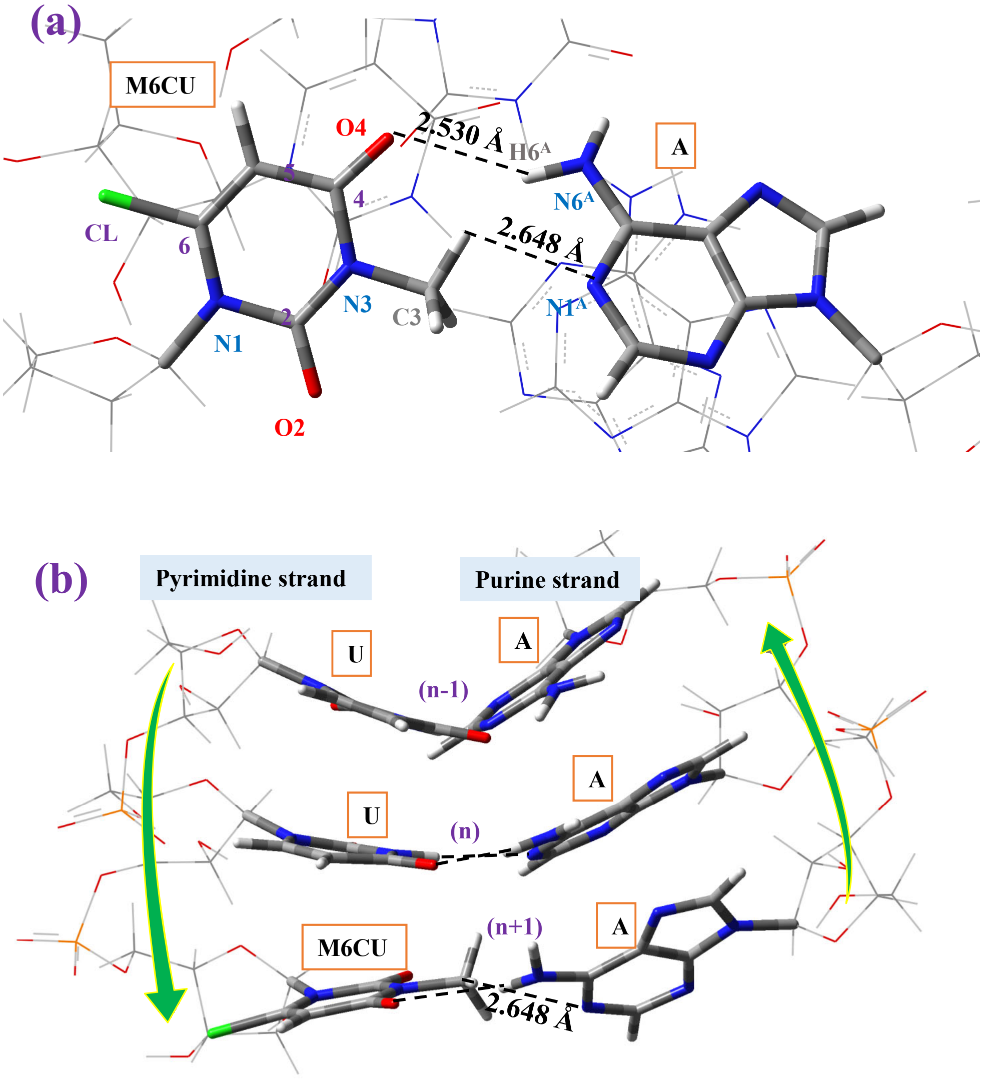

Figure S13. Two views of the M6CU effect inserted into the optimized double strand 5′-UUU-3′ microhelix at the M06-2X/6-31G(d,p) level in the A-type. The values of the intermolecular H-bonds are included. (a) With M6CU values in plane n+1. (b) With the deformation in all planes.

Table S1. Geometrical parameters, bond lengths (in A) and angles (in degrees) of M6CU at different theoretical methods and basis set.

Parameters	M6CU					uracil	6-CU	3MU		M5CU
	Monomer							monomer		
	B3LYP		M06-2X	MP2		MP2				
	(a)	(b)	(a)	(a)	(b)	(a)	(a)	(a)	(b)	(a)
Bond lengths										
N1-C2	1.399	1.394	1.393	1.395	1.389	1.390	1.395	1.388	1.383	1.386
C2-N3	1.388	1,381	1.383	1.388	1.379	1.384	1.382	1.389	1.382	1.392
N3-C4	1.418	1.418	1.409	1.413	1.411	1.408	1.408	1.417	1.411	1.412
C4-C5	1.459	1.454	1.461	1.454	1.451	1.457	1.456	1.455	1.451	1.466
C5=C6	1.347	1.341	1.341	1.349	1.347	1.351	1.351	1.350	1.346	1.352
N1-C6	1.370	1.364	1.368	1.370	1.363	1.376	1.374	1.372	1.366	1.372
N1-H	1.010	1.009	1.010	1.009	1.012	1.008	1.001	1.008	1.010	1.008
C2=O	1.218	1.211	1.211	1.225	1.216	1.224	1.222	1.227	1.217	1.226
N3-C3	1.467	1.465	1.461	1.463	1.460	-	-	1.463	1.459	1.465
C4=O	1.221	1.214	1.213	1.230	1.219	1.227	1.222	1.230	1.220	1.226
C5-H	1.080	1.077	1.079	1.077	1.079	1.077	1.077	1.077	1.079	1.719[b]
C6-Cl	1.739	1.724	1.726	1.722	1.708	-	1.722	-	-	-
Bond angles										
N-C2-N	114.3	115.0	114.3	113.7	114.7	112.4	112.5	114.2	114.6	114.0
C-N3-C4	125.2	124.8	125.4	125.8	125.5	128.9	128.6	126.0	125.7	126.6
N-C4-C	115.7	115.4	115.7	115.5	115.1	113.2	113.7	114.4	114.6	113.6
C4-C5=C	119.2	119.7	118.9	119.3	119.6	119.9	119.4	120.5	120.3	120.9
N1-C6=C5	121.8	121.7	122.1	121.5	121.7	121.8	122.2	120.9	121.0	120.5
C2-N1-C6	123.8	123.4	124.1	124.1	123.3	123.9	123.5	124.0	123.8	124.3
C2-N1-H	115.0	115.6	115.1	115.0	115.7	114.9	115.6	114.5	114.7	114.8
N1-C2=O	120.6	121.0	120.8	121.1	121.6	123.2	122.6	122.2	122.1	122.5
C2-N3-C3	118.2	116.2	118.3	118.1	115.6	115.3	-	115.4	115.4	115.1
N3-C4=O	120.0	120.9	120.0	120.0	121.0	120.6	120.6	121.2	121.0	121.9
C5=C6-Cl	123.4	123.3	123.2	123.4	123.3	122.8[a]	123.0	123.3[a]	123.0[a]	-
C6-N1-H	121.2	121.1	121.3	120.9	120.9	121.2	120.8	121.5	121.5	120.9

(a) With the 6-31G(d,p) basis set. (b) With the 6-311++G(3df,pd) basis set. [a] With H6. [b] CL.

Table S2. NBO total atomic charges on the atoms of M6CU with different DFT methods and basis set.

Atom	M6CU					uracil	6-CU	3MU
	B3LYP		M06-2X	MP2		MP2		
	(a)	(b)	(a)	(a)	(b)	(a)		
N1	-0.650	-0.624	-0.677	-0.745	-0.714	-0.739	-0.752	-0.731
C2	0.836	0.836	0.866	1.025	1.025	1.014	1.019	1.017
N3	-0.497	-0.510	-0.526	-0.612	-0.625	-0.784	-0.784	-0.614
C4	0.661	0.670	0.688	0.824	0.836	0.820	0.824	0.825
C5	-0.400	-0.375	-0.413	-0.445	-0.421	-0.451	-0.452	-0.445
C6	0.193	0.210	0.192	0.274	0.301	0.119	0.273	0.120
H1	0.464	0.426	0.474	0.475	0.430	0.462	0.475	0.462
O2	-0.619	-0.627	-0.631	-0.731	-0.729	-0.730	-0.719	-0.737
C3	-0.486	-0.359	-0.493	0.416	-0.261	0.468[a]	-	-0.417
O4	-0.585	-0.604	-0.595	-0.693	-0.705	-0.694	-0.686	-0.705
H5	0.282	0.248	0.290	0.280	0.238	0.268	0.281	0.261
Cl	0.045	0.063	0.053	0.047	0.054	0.248[a]	0.049	-

(a) With the 6-31G(d,p) basis set. (b) With the 6-311++G(3df,pd) basis set. [a]With hydrogen atom.

Table S3. Theoretical computed total energies (A.U.), Gibbs energies (A.U.), rotational constants (GHz), entropies (J mol^{-1} K^{-1}), dipole moments (Debyes), and constant volume heat capacity (C_v, J mol^{-1} K^{-1}) in the monomer form of M6CU, uracil, 6-CU and 3MU molecules with different methods and basis set.

	M6CU					uracil	6-CU	3MU
	B3LYP		M06-2X	MP2				
Parameters	(a)	(b)	(a)	(a)	(b)	(a)	(a)	(a)
Total energy + ZPE	-913.62332[a]	-913.81614[a]	-913.41136[a]	-911.75926	-912.42209[b]	-413.57814	-872.60853	-452.72840
Gibbs energy	-913.65843[a]	-913.85129[a]	-913.44609[a]	-911.79444		-413.60874	-872.64140	-452.76146
Rotational constants: A	1.984	1.999	2.003	1.979	1.998	3.879	2.005	2.376
B	0.848	0.856	0.857	0.855	0.864	1.999	1.136	1.973
C	0.596	0.602	0.602	0.599	0.605	1.319	0.725	1.085
Entropy: Total	397.81	398.02	393.09	398.99		333.00	363.49	369.66
Translational	172.05	172.05	172.05	172.05		167.61	170.75	169.08
Rotational	126.15	126.02	126.02	126.11		116.48	123.94	119.41
Vibrational	99.62	99.91	95.02	100.83		48.91	68.80	81.17
Dipole moment	2.278	2.506	2.284	2.937	3.088	4.146	3.554	4.472
Heat capacity, C_v	136.98	136.73	135.39	137.19		98.41	114.68	369.66

(a) With the 6-31G(d,p) basis set. (b) With the 6-311++G(3df,pd) basis set.

Table S4. Global chemical descriptors (eV) calculated in the monomer and trimer forms of M6CU at different theoretical levels, and in uracil, 6-CU and 3MU molecules with the MP2 method.

Descriptor	Monomer					uracil	6-CU	3MU	
	B3LYP		M06-2X	MP2		MP2			
	(a)	(b)	(a)	(a)	(b)	(a)	(a)	(a)	(b)
HOMO	−7.02	−7.35	−8.46	−9.99	−10.10	−9.88	−10.12	−9.74	-9.92
LUMO	−1.33	−1.74	−0.24	2.45	1.01	2.69	2.34	2.80	0.67
IP	7.02	7.35	8.46	9.99	10.10	9.88	10.12	9.74	9,92
EA	1.33	1.74	0.24	−2.45	−1.01	−2.69	−2.34	−2.80	-0.67
E_g	5.69	5.61	8.22	12.44	11.10	12.57	12.46	12.54	10.50
χ	4.18	4.54	4.35	3.77	4.54	3.59	3.89	3.47	4.62
η	2.84	2.80	4.11	6.22	5.55	6.29	6.23	6.27	5.30
S	0.35	0.36	0.24	0.16	0.18	0.16	0.16	0.16	0.19

(a) With the 6-31G(d,p) basis set. (b) With the 6-311++G(3df,pd) basis set. 1 Hartree = 27.2114 eV

Table S5. Calculated harmonic wavenumbers (ν^{cal}, cm^{-1}) in the isolated state (monomer form), relative infrared intensities (A, %), relative Raman intensities (S, %), Raman depolarization rations for plane (P) and unpolarized (U) incident light and force constants (f, mDyne/Å) obtained in M6CU with two different DFT methods and the 6-31G(d,p) basis set, together with the scaled values (ν^{scal}, cm^{-1}) and its characterization according to notation of ref. [46].

Calculated						Scaled		Experimental		Ring n°	Characterization[a] (PED %)
B3LYP						B3LYP	M062X	IR	Raman		
ν^{cal}	A	S	P	U	f	ν^{scal}	ν^{scal}	ν^{exp}	ν^{exp}		
							--				
3650	18	52	0.23	0.37	8.47	3476	3484			30	ν(N1-H) (99%)
3278	1	57	0.27	0.43	6.93	3126	3104		3088 vw	28	ν(C5-H) (100%)
3206	0	27	0.73	0.85	6.69	3058	3069	3088 m			ν_{as}(C-H) in CH_3 (100%)
3149	2	51	0.75	0.86	6.46	3004	3009	3048 br	3021 vvw		ν_{as}(C-H) in CH_3 (100%)
3082	3	100	0.04	0.07	5.79	2941	2938	2962vw	2964 vw		ν_s(C-H) in CH_3 (100%)
1825	45	21	0.12	0.22	18.78	1755	1759	1730 s	1707 w	26	ν(C2=O) (62%) + ν(C4=O) (16%) + δ(N1-H) (10%)+ δ_s(C-H) in CH_3 (10%)
	--	--									
1772	100	24	0.26	0.42	20.94	1706	1716	1704 s	1612 w	25	ν(C4=O) (61%) + ν(C2=O) (20%) + δ(C5-H) (13%)
1677	26	16	0.10	0.18	13.86	1616	1605	1615, 1599 vs	1601 m	24	ν(C=C) (42%) + δ(N1-H) (15%) +δ(C5-H) (12%)+ ν(C4=O) (10%)
1519	1	16	0.65	0.79	1.49	1467	1420	1505 vs	1509 m		δ_{as}(C-H) in CH_3 (93%)
1505	1	13	0.75	0.86	1.39	1454	1417		1464 vvw		δ_{as}(C-H) in CH_3 (92%)
1500	39	5	0.56	0.72	3.70	1449	1401	1445 vs		21	ν(N1-C6) (44%) + δ(N1-H) (29%) + ν(ring) (15%) + δ_s(C-H) in CH_3 (12%)
1459	8	2	0.70	0.82	1.74	1435	1431				δ_s(C-H) in CH_3 (85%)
1410	24	2	0.73	0.84	3.80	1387	1397	1387 s	1387 w		ν(C2-N-C4) (70%) + ν(N-C3) (20%) + δ_s(C-H) in CH_3 (10%)
1318	1	0	0.32	0.48	1.67	1297	1290	1332 s	1336 vvw	23	δ(N1-H) (40%) + δ(C5-H) (38%) + δ(ring) (15%)
1294	4	9	0.49	0.66	2.74	1273	1283	1277 vw	1279 vs	19	ν(N3-C4) (32%) + δ_{as}(C-H) in CH_3 (31%) + δ(N1-H) (13%) + δ(C5-H) (13%)
	--	--									
1173	3	1	0.55	0.71	4.23	1154	1172	1171 w	1171 sh	18	ν(N3-C12) (40%) + ν(C2-N1) (30%) + δ(C5-H) (24%)
1160	0	2	0.75	0.86	1.03	1142	1135	1158 w	1156 vs		γ_{as}(C-H) in CH_3 (91%)
1157	1	10	0.28	0.43	1.93	1139	1129	1110 w	1112 s	17	ν(ring) (83%) + δ(C5-H) (10%)
1110	1	4	0.38	0.56	1.89	1093	1092		1093 vvw	16	δ(ring) (47%)+ δ(N1-H) (23%) + δ(C5-H) (21%)
1004	4	2	0.57	0.72	1.20	989	998	989 m	991 vvw		r(C-H) in CH_3 (40%) + 16, δ(ring) (37%)
954	11	0	0.62	0.77	5.09	939	946	954 m		14	ν(ring) (91%)
	--	--									
820	5	2	0.75	0.86	0.63	808	797	847 s	846 w	13	γ(C5-H) (55%) + γ(C4-C5) (27%) + γ(C4=O) (10%)
748	0	4	0.11	0.21	1.63	737	747	754 vs	752 s		ν(C-CL) (35%) + ν(ring) (25%) + γ(C-H) in CH_3 (20%)
740	4	0	0.75	0.86	3.09	729	735		720 vvw	11	γ(C2=O) (59%) + γ(N1-C-N-C4) (22%) + 10, γ(C4=O) (21%)
722	2	1	0.75	0.86	0.89	712	709	717 vw		10	γ(C4=O) (38%) + γ(C5-H) (36%) + γ(ring) (15%)
641	1	1	0.68	0.81	1.18	632	633	653 vs	655 vvw	6	r(ring) (85%) + r(C-H) in CH_3 (12%)
603	2	0	0.75	0.86	0.88	595	595		597 vs		γ(N1-C-C5) (41%) + 8, γ(N1-H) (30%) + γ(ring) (27%)
581	1	11	0.13	0.23	2.53	573	581			12	ν(ring) (65%) + ν(C-CL) (11%)
534	1	3	0.50	0.67	1.46	527	524		539 s	7	δ_{as}(ring) (83%)
490	10	2	0.75	0.86	0.19	484	482			8	γ(N1-H) (64%) + δ(ring) (23%)

[a]Characterization obtained with B3LYP. PED less than 10% was excluded. Abbreviations: ν, stretching; δ, in-plane bending; γ, out-of-plane bending; r, rocking.

Table S6. Calculated harmonic wavenumbers (ν^{cal}, cm^{-1}) in low modes of the isolated state (monomer form), relative infrared intensities (A, %), Raman activities (S, %), Raman depolarization rations for plane (P) and unpolarized (U) incident light and force constants (f, mDyne/Å) obtained in M6CU with two different DFT methods and the 6-31G(d,p) and 6-311++G(3df,pd) basis set, together with the scaled values (ν^{scal}, cm^{-1}) and its characterization according to notation of ref. [46].

Calculated														Scaled			Experimental	Ring n°	Characterization[a] (PED %)
B3LYP											M062X			B3LYP		M062X	Raman		
(a)						(b)					(a)			(a)	(b)				
ν^{cal}	A	S	P	U	f	ν^{cal}	A	S	P	U	ν^{cal}	A	S	ν^{scal}	ν^{scal}	ν^{scal}	ν^{exp}		
405	31	1	0.22	0.37	0.80	409	3	1	0.12	0,22	414	29	1	401	403	406	380 s	3	δ(OCNCO) (92%)
367	6	9	0.40	0.57	1.18	369	1	4	0.28	0,44	375	6	7	363	363	368		5	δ(ring) (51%) + δ(C-CL) (45%)
344	9	0	0.59	0.74	0.24	344	1	0	0.68	0,81	360	9	1	341	338	354			γ(C-H) in CH_3 (52%) + δ(ring) (45%)
262	3	0	0.75	0.86	0.19	263	0	0	0.75	0,86	261	4	0	260	257	258	254 w	1	puckering on N3 (50%) + γ(N-C3) (40%)
238	2	2	0.75	0.85	0.35	239	0	1	0.75	0,86	240	2	2	237	233	238	211 s		r(ring) (85%) + r(C-H) in CH_3 (12%)
167	1	1	0.75	0.86	0.10	162	0	0	0.75	0,86	170	1	1	167	156	170		2	γ(ring) mainly in C5, N1 (92%)
143	2	0	0.75	0.86	0.11	144	0	0	0.75	0,86	146	1	0	144	138	147			γ(ring) mainly in O2, N1 (70%)
100	2	0	0.75	0.86	0.02	99	0	0	0.75	0,86	103	0	0	101	93	105	105 vs	1	γ(N-C3) (70%) + γ(ring) (15%) + γ(C-H) in CH_3 (10%)
65	0	0	0.75	0.86	0.00	6	0	0	0.75	0,86	92	2	0	67	55	95	82 s		γ(C-H) in CH_3 (97%)

(a) With the 6-31G(d,p) basis set. (b) With the 6-311++G(3df,pd) basis set. [a]Characterization obtained with B3LYP. PED less than 10% was excluded.
Abbreviations: ν, stretching; δ, in-plane bending; γ, out-of-plane bending; r, rocking.

Table S7. Scaled wavenumbers (ν^{scal}, cm^{-1}) in the 4000-500 cm^{-1} range determined with the PSE procedure at the CAM-B3LYP/6-311++G(3df,pd) levels in the solid state (trimer form) of M6CU in *A*- and *B*-forms, together with the relative IR intensities (A, %), and Raman relative intensities (S, %). Comparison with the experimental wavenumbers (ν^{exp}) in the solid state with the IR and Raman intensities. The main characterization with the ring mode number and the %PED in parenthesis.

CAM-B3LYP, *A*-form			CAM-B3LYP, *B*-form			experimental		Ring n^{o}	Characterization^{a} (PED %)
ν^{scal}	A	S		A	S	IR	Raman		
3480, 3134, ***3113***	10	9	***3137***, 3134	22	80	3088 m	3088 vw	30	ν(N1-H) (100%)
**3096**, *3089*, 3088	100	100	**3103**, *3087*, 3074	100	60			28	ν(C5-H) (75%) + ν(N1-H) (25%)
3074, 3072, 3065	0	3	3068, 3066, ***3036***	24	100	3048 br	3021 vvw		ν_{as}(C-H) in CH_3 (100%)
3024, 3020, 3016	0	6	3024, 3021, 3016	0	8				ν_{as}(C-H) in CH_3 (100%)
2961, 2960, **2956**	1	16	2961, 2958, ***2956***	1	24	2962vw	2964 vw		ν_{s}(C-H) in CH_3 (100%)
**1767**, 1751, *1739*	13	10	**1751**, 1747, *1721*	18	25	1730 s	1707 w	26	ν(C2=O) (57%) + ν(C4=O) (29%) + δ(N1-H) (10%)
1700, 1673, ***1658***	77	12	1701, 1689, ***1660***	83	8	1704 s	1636 vw	25	ν(C4=O) (55%) + ν(C2=O) (20%) + δ(C5-H) (16%)
1639, *1638*, 1632	15	6	1644, ***1641***, 1635	16	6	1615, 1599 vs	1612,1601 m	24	ν(C=C) (50%) + δ(N1-H) (20%) +δ(C5-H) (12%)+ ν(C4=O) (11%)
1526, ***1523***, 1486	7	8	1522, ***1519***, 1513	9	12	1505 vs	1509 m	23	δ(N1-H) (48%) + ν(N1-C6) (29%) + ν(C2=O) (13%)
1470, 1468, 1461	1	1	**1471**, 1467	2	1				δ_{as}(C-H) in CH_3 (87%) + δ(N1-H) (10%)
1457, 1456	0	1	1459, 1457	0	1		1464 vvw		δ_{as}(C-H) in CH_3 (100%)
1445, **1439**, *1435*	7	1	**1453**, *1449*, 1439	7	1	1445 vs			δ_{s}(C-H) in CH_3 (57%) + ν_{as}(C-N3-C) (28%)
1392, **1387**	2	0	*1394*, 1391, **1387**	2	1	1387 s	1387 w	21	ν(C-N3-C) (34%) + δ_{s}(C-H) in CH_3 (30%)+ ν(N-C3) (18%) + δ(N1-H) (17%)
1346, ***1335***, 1313	1	1	1349, 1336, 1333	1	1	1332 s	1336 vvw	19	δ(C5-H) (40%) + δ(N1-H) (29%) + ν(C-N1-C) (21%)
1276, 1275, 1270	0	5	*1278*, 1270	0	7	1277 vw	1279 vs		ν(N3-C4) (51%) + γ_{s}(C-H) in CH_3 (23%) + δ(N1-H) (11%) + δ(C5-H) (10%)
1181, 1170, *1168*	1	2	1186, **1178**, *1171*	1	4	1171 w	1171 sh	18	ν(N3C3) (28%)+δ(C5-H)(27%)+ν_{as}(NC6C) (17%)+δ(N1H)+ν(C4C5)(10%)
1163, 1154, 1148	0	4	1169, 1152, *1148*	1	5	1158 w	1156 vs	17	ν(N1-C2) (38%) + ν(C4-C5) (26%) + ν(N3-C3) (21%)
1133	0	0	1133	0	0				γ_{as}(C-H) in CH_3 (99%)
1110, 1104, **1096**	1	2	*1108*, 1103, 1101	0	3	1110 w	1112 s	16	δ(ring) (54%) + δ(C5-H) (22%) + δ(N1-H) (20%)
							1093 vvw		
993, **990**, 985	2	0	996, 989, 985	1	0	989 m	991 vvw		r(C-H) in CH_3 (60%) + 16, δ(ring) (32%)
**958**, 956, 952	3	0	958, **955**, 952	3	0	954 m		14	ν_{as}(ring) mainly in N1-C6-C (70%) + ν(C-CL) (25%)
891, 885, 856	3	0	**898**, 873, 844	3	0			13	γ(C5-H) (58%) + γ(N1-H) (37%)
**849**, 825	1	0	**841**, 829, 823	1	0	847 s	846 w	8	γ(N1-H) (68%) + γ(C5-H) (27%)
772, 768, **765**	1	0	773, 770, 768	0	0	754 vs	752 s	11	γ(ring) mainly in C2=O (96%)
752, *747*, 744	0	2	755, 747, *746*	0	4			12	ν(ring) (65%)+ ν(C-CL) (18%) + γ(C-H) in CH_3 (16%)
733, 721, 699	0	0	721, 700, 698	0	0	717 vw	720 vvw	10	γ(C4=O) (38%) + γ(C2=O) (24%) + γ(C5-H) (23%) + γ(N1-H) (15%)
**650**, 641, 639	2	0	**654**, 643, 640	2	0	653 vs	655 vvw	6	r(ring) (82%) + r(C-H) in CH_3 (11%)
607, 605, 603	0	0	607, 603, 602	0	0			4	γ(N-C5-C) (75%) + γ(ring) (19%)
591, 587, **580**	1	3	597, 586, 580	1	4		597 vs	5	ν(C-CL) (49%) + δ_{as}(ring) (46%)
534, 529, 527	5	1	***535***, 529, 527	3	2		539 s	7	δ(ring) (99%)

Table S8. Scaled wavenumbers (ν^{scal}, cm^{-1}) in the far-IR region (< 500 cm^{-1}) determined with the PSE procedure at the B3LYP/6-311++G(3df,pd) and CAM-B3LYP/6-311++G(3df,pd) levels in the trimer form of *A*- and *B*-forms in M6CU, together with the relative IR intensities (A, %), and Raman relative intensities (S, %). Comparison with the experimental wavenumbers (ν^{exp}) in the solid state with the IR and Raman intensities. The main characterization with the ring mode number and the %PED in parenthesis.

CAM-B3LYP, *A*-form			CAM-B3LYP, *B*-form			experimental	Ring n°	Assignment
ν^{scal}	A	S		A	S	Raman		
510, 423, 416, 404	1	0	428, 411, 404	1	0		3	δ(OCNCO) mainly in C4=O (82%) + δ(C-H) in CH_3 (14%)
369, 367, 364	0	1	372, 368, 364	0	1	380 s	5	δ(C-CL) (80%) + δ(ring) (15%)
**350**, 343, 340	1	0	359, 351, 343	1	0			δ(C4=O) (40%) + δ(N3-C3) (32%) + δ(C2=O) (15%)
261, 257, 254	0	0	264, 258, 254	0	0		1	τ(ring) on C2-N3-C4 axis (86%)
243, 237, 235	0	0	247, 239, 236	0	0	254 w		δ(ring) (51%) + δ(C-CL) (45%)
177, 165, 160	0	0	179, 167, 162	0	0	211 s	2	Puckering N1 (100%)
152, 147, 142	0	0	156, 151, 147	0	0			Puckering on O2, O4 (94%)
100, 96	0	0	117, 98	0	0	105 vs	1	puckering on N3 (73%) + γ(CH_3) (25%)
94, 92, 71	0	0	95, 93, 74	0	0			r(ring) (85%) + r(C-H) in CH_3 (12%)
67, 59, *55*	0	1	72, 67, 63	0	1	82 s		Γ(CH_3) (100%)
53, 43, 39	0	0	60,*56*, 42	0	0			H-bond shearing
31	0	0	39	0	0			Cogwheel
30, 12	0	0	32, 15	0	0			Butterfly
9, 8	0	0	11	0	0			Torsion (twist)
